# 14. Discogenic Low Back Pain

**DOI:** 10.1111/papr.70062

**Published:** 2025-07-27

**Authors:** Wouter K. M. van Os, Ricardo Alvarez‐Jimenez, Steven P. Cohen, Milan P. Stojanovic, Ricardo Ruiz‐Lopez, Jan Van Zundert, Jan Willem Kallewaard

**Affiliations:** ^1^ Department of Anesthesiology, Pain and Palliative Medicine Amsterdam University Medical Center Amsterdam the Netherlands; ^2^ Anesthesiology, Neurology, Physical Medicine & Rehabilitation and Psychiatry and Behavioral Sciences Northwestern University Feinberg School of Medicine Chicago Illinois USA; ^3^ Anesthesiology and Physical Medicine & Rehabilitation, Walter Reed National Military Medical Center Uniformed Services University of the Health Sciences Bethesda Maryland USA; ^4^ Department of Anesthesiology, Critical Care and Pain Medicine Service VA Boston Healthcare System Boston Massachusetts USA; ^5^ VA Bedford Healthcare System Bedford Massachusetts USA; ^6^ Harvard Medical School Boston Massachusetts USA; ^7^ Spine and Pain Surgery Clinica Vertebra Barcelona Spain; ^8^ Department of Anesthesiology, Intensive Care, Emergency Medicine and Multidisciplinary Pain Center Ziekenhuis Oost‐Limburg Genk Belgium; ^9^ Department of Anesthesiology, Pain Medicine and Neurology Maastricht University Medical Center Maastricht the Netherlands; ^10^ Department of Anesthesiology and Pain Medicine Rijnstate Ziekenhuis Velp the Netherlands

**Keywords:** chronic discogenic low back pain, discography, evidence‐based medicine, interventional therapy, intradiscal therapy

## Abstract

**Introduction:**

Discogenic low back pain can be severely disabling, clinically challenging to diagnose, and expensive to treat. Disc degeneration is characterized by disc dehydration, which diminishes the ability of the disc to distribute pressure, making it more susceptible to damage, and leading to annular tears, fissures, and a higher incidence of herniation. Furthermore, the abnormal annular in‐growth of nerves and inflammation of the disc increase the number and sensitivity of nociceptors, leading to chronic discogenic low back pain (CDLBP). The purpose of this article was to review the current literature.

**Methods:**

In this narrative review, the literature on the diagnosis and treatment of discogenic low back pain was summarized.

**Results:**

Symptoms and findings during physical examination may guide the diagnostic process but are not specific or sensitive regarding CDLBP. Magnetic resonance imaging (MRI) can rule out other pathology and provides a basis for the decision about whether to perform pressure‐controlled provocative discography, the current diagnostic standard. Conservative care includes pain education programs, structured exercise therapies, psychological interventions, and pharmacological treatment. Various minimally invasive interventional treatment strategies for refractory CDLBP exist, of which biacuplasty or cooled radiofrequency can be used as therapeutic options. Promising new treatments include intradiscal injection of mesenchymal stem cells and platelet‐rich plasma, radiofrequency ablation of the sinuvertebral and basivertebral nerves, dorsal root ganglion stimulation, and spinal cord stimulation. Future research regarding the safety and efficacy of these treatments should include large randomized controlled trials with strict inclusion criteria and longer follow‐up periods. A primary focus should be on increasing the evidence base for diagnosing discogenic low back pain.

## 1 Introduction

This narrative review on discogenic low back pain is an update of the article published in the series “Evidence‐based Interventional Pain Medicine According to Clinical Diagnoses” [1].

Chronic low back pain is a major cause of disability, affecting approximately 619 million people or roughly 8% of the world population [2]. Among those suffering from chronic low back pain, approximately 40% of cases are believed to be related to the intervertebral discs, often described as discogenic pain [2, 3]. However, in clinical practice, it is rare to encounter a patient whose chronic low back pain is secondary solely to a single pathology without contribution from other conditions. Discogenic lumbar pain shares clinical signs and symptoms that are difficult to differentiate from other sources of lumbar mechanical or even radicular pain, making diagnosis of an internally disrupted disc as the primary source of pain challenging. This review provides a concise summary of diagnostic and treatment recommendations for chronic discogenic low back pain (CDLBP), with emphasis on new recommendations based on recent publications.

### 1.1 Anatomy of the Intervertebral Disc

**FIGURE 1:**
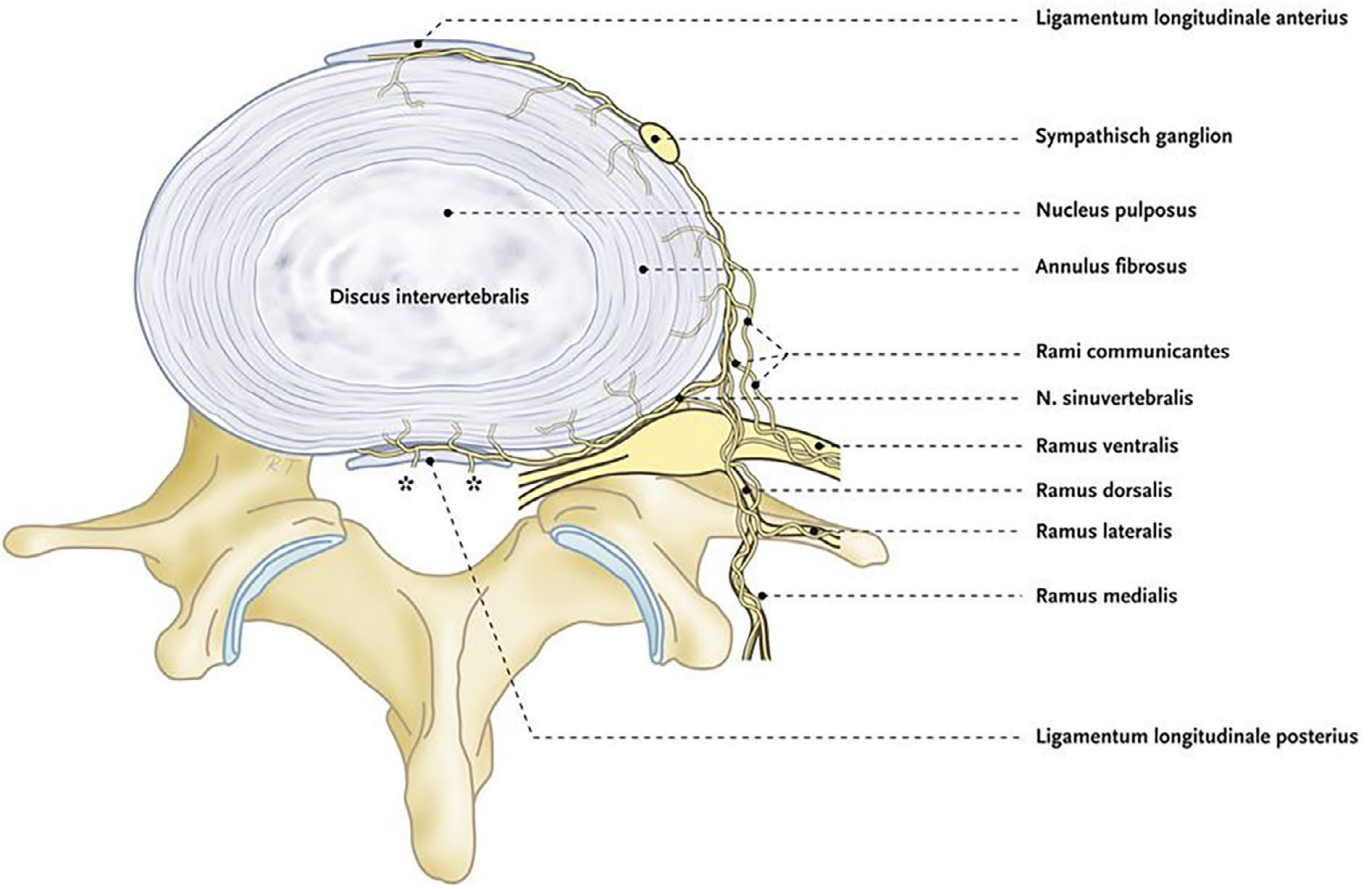
Schematic drawing of the lumbosacral innervation. Connections to the dural nerve plexus. Illustration Rogier Trompert Medical Art. http://www.medical‐art.eu.

The intervertebral disc (IVD) is composed of the nucleus pulposus (NP) and the annulus fibrosus (AF), with the anatomically discrete vertebral endplates (VEP) separating discs from their adjacent vertebral bodies (Figure 1). The corpora vertebrae lie above and below the disc. The body's axial load is distributed mainly onto the IVD, with a smaller burden being borne by the two facet joints, the anterior and posterior ligaments, and core stabilizing muscles, especially during dynamic stress [4]. As the body ages, discs shrink, the facet joints undergo hypertrophy, and ligaments buckle over; the axial load shifts posteriorly. The healthy disc is avascular, and its nutrition depends on diffusion via the AF and the vertebral end plate endings, making the disc an immunologically privileged structure.

### 1.2 Innervation

The NP and the innermost part of the AF are not innervated. However, sensory mechanoreceptors in the outermost third of the AF of the IVD contain afferent fibers that traverse via sympathetic pathways. Their cell bodies are primarily located in the ganglia spinalia (dorsal root ganglia, DRGs) between C8 and L2, that is, at the levels at which sympathetic nerve fibers leave the spinal cord.

Before they enter the DRG, the afferent fibers from the ventral part of the IVD form a network with fibers from the ligamentum longitudinale anterius (LLA), come together in the sympathetic trunk and travel along the rami communicantes, whereas afferent fibers from the dorsal part of the IVD form a network with fibers from the ligamentum longitudinale posterius (LLP), parts of the vertebral body and the ventral dura, and travel along the sinuvertebral nerves and posterior rami communicantes (Figure 1) [5].

Within the posterior and anterior longitudinal ligaments, many transverse connections give the plexus sinuvertebralis a denser configuration, giving branches in multiple directions: horizontal, cranial and caudal at every vertebral body level. The complex network of anastomoses surrounding the IVD contributes to patients experiencing pain on the opposite side, or even at a different level than the site of pathology [6, 7].

Some studies have reported that blocking (or stimulating) the L2 spinal nerve could assist in the diagnosis of lumbar discogenic pain and possibly provide therapeutic benefit [8, 9, 10]. Although one randomized controlled trial (RCT) reporting benefit after ramus communicans denervation in patients with single‐level discogenic pain suggested clinically relevant nociceptive sympathetic innervation [11], a negative double‐blinded, sham‐controlled RCT that found no benefit called into question this assumption [11, 12].

## 2 Methodology

This narrative review is based on the article “Discogenic Low Back Pain” published in 2010 [1]. In 2015, an independent company, Kleijnen Systematic Reviews (KSR) performed a systematic review of the literature for the period 2009–2015 based on existing systematic reviews (SRs) and RCTs. For the current article, an updated search was conducted for the period 2015–2022 using the terms “discogenic” and “pain” in combination with specific interventional pain management techniques such as “methylene,” “steroid,” “biacuplasty,” or “thermal.” Additionally, authors selected relevant missing articles based on reference lists and precision literature reviews.

## 3 Pathophysiology and Etiology of Discogenic Low Back Pain

Discogenic low back pain (DLBP) is a complex condition that results from degeneration or damage to the IVD. These discs are responsible for the majority of the axial load, acting as the spine's shock absorbers, cushioning the vertebrae during movement, and facilitating segmental motion. The development of discogenic LBP may occur secondary to a combination of factors including acute physical trauma (e.g., a torsional event) and chronic stressors that include prolonged sitting or improper lifting techniques. These external pressures can exacerbate the natural aging process leading to significant structural changes [13]. The complexity of the local environment combining traumatic and chemical insults to evoke inflammation are difficult to reproduce in experimental setting and therefore difficult to study as a model of discogenic low back pain. Nearly all experimental animal study models of degenerative disc disease mimic discogenic pain by inflicting injury to the disc by a puncture, surgical removal of the NP, leakage of the NP after removal of a facet joint, or injection of a variety of inflammatory substances (e.g., inflammatory cytokines, complete Freund's adjuvant, phosphate buffered saline) [14]. Therefore, it is difficult to draw conclusions on the “unaffected/atraumatic” degenerative human disc and its structural changes.

As discs degenerate, they lose their water content, which reduces their height and shock‐absorbing capabilities. This dehydration affects the disc's ability to evenly distribute pressure, making it more susceptible to damage [8]. Additionally, the AF, the disc's outer layer, may develop fissures that allow the inner NP to protrude, potentially causing herniation [13]. As the number of functional annular lamellae, normally between 15 and 25, is reduced, the load borne by the remaining lamellae increases, eventually exceeding the nociceptive threshold. This process may be further complicated by the release of pro‐inflammatory cytokines such as tumor necrosis factor‐α (TNF‐α) and interleukin‐1β (IL‐1 β), which stimulate the production of vascular endothelial growth factor (VEGF) and accelerate the in‐growth of blood vessels, a process known as neovascularization [8, 15]. The release of IL‐1 β (and consequently neurotrophic factors and nerve growth factor) and diminished aggrecan may also play a role in nerve in‐growth, a process known as neoinnervation [16, 17].

In pathological conditions (i.e., patients with a history of lower back pain, degenerative low back changes or related comorbidities), the innervation of the IVD is no longer limited to the outermost third of the AF but encompasses more abundant nerve endings and increased morphological complexity [9]. In patients with known IVD pathology, nerve in‐growth into the NP has been described [9].

The abnormal in‐growth of blood vessels and nerve fibers into regions of the disc that are typically devoid of such structures has several consequences. Nerve in‐growth results in a greater volume of nerve endings to be sensitized deeper into the disc, which can result in a form of “chemical sensitization”. The increased expression of VEGF can also lead to nociceptive sensitization as well as the stimulation of osteophyte formation. Changes in the disc's collagen composition also play a role, affecting its structural integrity and function [18, 19]. Risk factors exacerbating disc degeneration include genetics, male sex, obesity, smoking, and repetitive physical activities that increase mechanical stress [13, 20, 21].

## 4 Diagnosis

### 4.1 History

CDLBP is often described as a midline nociceptive pain [22] exacerbated by sitting [23], flexion and axial rotation (twisting) especially under high spinal loads [24], as well as coughing and sneezing. It is more likely that facetogenic, sacroiliac (SI) joint or myofascial pain is midline or symmetrical but is similarly devoid of radicular signs (motor/sensory/reflex changes) [25, 26].

Referred pain above the knee is common, and pain may occasionally extend below the knee albeit in a non‐dermatomal distribution [27, 28, 29]. Discogenic pain originating from the L3‐L4 level typically radiates to the front of the thigh, L4‐L5 to the lateral and sometimes posterior thigh, while L5‐S1 extends to the back of the thigh [29].

An acute exacerbation of chronic low back pain might be discogenic in origin (i.e., from an acute annular tear) [30]. But because symptoms generally lack both sensitivity and specificity, they are often unhelpful in rendering a diagnosis. However, the presence of “centralization”—thephenomenon whereby pain that radiates to the extremities (such as the legs in the case of low back pain) moves back toward the center of the spine (lower back) with certain movements or positions—increases the probability of discogenic origin [31]. The presence of Hancock criteria or red flags such as unexplained weight loss, fever, or neurological deficits necessitates immediate medical evaluation to rule out serious underlying conditions [32].

### 4.2 Physical Examination

Most of the characteristics found during physical examination are not specific for CDLBP, making it difficult to distinguish from other causes of chronic low back pain. Some suggestive findings include pain provoked by forward flexion in either a straightened or sitting position and midline > paraspinal tenderness (i.e., provoking pain when applying pressure over the spinous process, or Federung test) [30, 33, 34]. Other studied tests are the bone vibration test (BVT) and the centralization phenomenon. In the BVT, a blunt vibratory object (a 128 Hz tuning fork or electric vibrator) transmits vibration signals via the spinous processes of the vertebrae in the painful area. The BVT is positive if pain is provoked when stimulating a specific spinous process. In 1994, Yrjämä and Vanharanta [35] conducted a small study in 57 patients where its diagnostic value was studied against provocative discography as the reference standard, finding a sensitivity and specificity of 71% and 63%. When patients with failed back surgery and herniated discs were excluded, the sensitivity and specificity increased to 96% and 72%, respectively. Combined with imaging techniques such as magnetic resonance imaging (MRI), BVT might be useful as a quick, safe and inexpensive screening tool [36, 37, 38].

The centralization phenomenon (as described by McKenzie) is the occurrence of recognizable pain in the midline upon lateral movement of the spine [39]. A recent systematic review performed by Han et al. [31] demonstrated that it can be useful in the identification of discogenic pain. Based on four studies containing 218 patients, the authors reported a sensitivity of 41.2 (33.2–49.6) and a specificity of 85.9 (75.6–93.0). However, the low sensitivity and lack of standardization limit its use.

Physical examination should also include SI joint pain provocation tests and neurological examination [31, 40]. Although disc pathology plays a role in both, signs on clinical examination are different from those found in radicular disorders. These include a negative straight leg raise, (crossed) Lasegue's sign, negative bowstring, negative femoral stretch tests, as well as normal electrodiagnostic testing [41]. These tests can help guide the differential diagnosis, but clinical diagnosis is best confirmed with imaging studies.

### 4.3 Imaging

Computed tomography (CT) and MRI are highly effective in demonstrating anatomical abnormalities and are crucial in the diagnostic process regarding the differential diagnosis of discogenic pain.

### 4.4 Single Photon Emission Computed Tomography

The (single photon emission) computed tomography (SPECT) has a role in diagnosing skeletal causes of chronic low back pain, such as spondylitis, facet joint arthritis, osteophyte formation, and fractures [42, 43]. Uptake in the facet joints on SPECT suggests these are the source of back pain [31]. SPECT seems a useful imaging technique in the diagnostic evaluation of patients with chronic spinal pain, especially when other imaging techniques are inconclusive [44]. Therefore, SPECT could be a helpful tool for diagnosing chronic discogenic pain.

### 4.5 MRI

**FIGURE 2:**
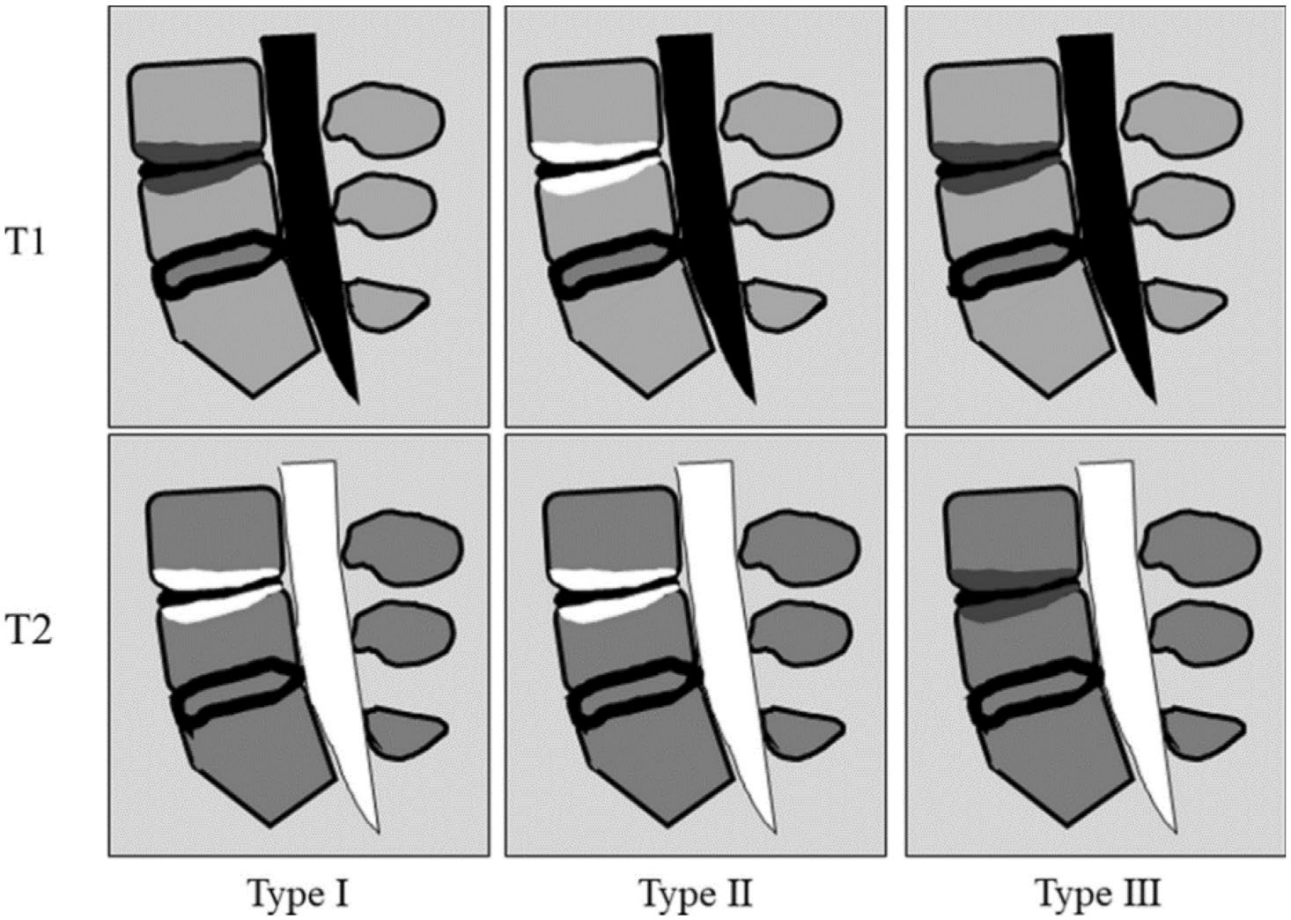
Sagittal magnetic resonance images showing types I, II, and III Modic changes. Type I: Low intensity on T1‐weighted images (T1WI), high on T2‐weighted images (T2WI). Type II: High intensity on both T1WI and T2WI. Type III: Low intensity on both T1WI and T2WI. Retrieved from Manabe et al. [48].

MRI is the most commonly used non‐invasive diagnostic tool for discogenic pain. Signs of disc degeneration (grades 1–5) on the Pfirmann scale, high intensity zones (areas of hypodensity in the posterior AF), annular fissures, and Modic (type 1: vertebral endplate subchondral bone edema, type 2: fatty degeneration as an indication for inflammation, and type 3: the hardening or thickening of the bone beneath the cartilage of the vertebral endplate; Figure 2) changes increase the likelihood of CDLBP [31, 45]. These findings alone exhibit area under the curves ranging from 0.563 to 0.809. However, combinations of various signs significantly enhance their diagnostic value to an area under the curve of 0.861 [46]. Nevertheless, MRI has limited diagnostic value given that its correlation with the clinical findings is poor [47]. However, it can provide a solid basis for deciding whether to perform discography. Moreover, other (mostly soft tissue) causes of pain including spinal stenosis or compression of radicular nerves by herniated discs can be demonstrated by MRI.

### 4.6 Discography

After symptoms and signs on physical examination are identified and other causes of the chronic low back pain are excluded, provocative discography can serve as a valuable tool to diagnose CDLBP [49, 50, 51, 52]. Provocative discography is an invasive diagnostic procedure in which contrast is injected into the NP under a controlled gradually increasing pressure. Provocative discography may identify which suspected disc is the pain generator by reproducing concordant pain (positive disc) on disc stimulation. In a negative (control) disc, no pain or discordant pain is produced.

It is hypothesized that the injected contrast agent increases pressure within the disc and irritates the nerve endings residing in radial fissures in the AF and, as a result, provokes pain [53]. The direction of contrast leakage can categorize discogenic pain into two types: annular disruption‐induced low back pain (IAD) and internal endplate disruption‐induced low back pain (IED) with leakage toward the outside of the disc through a radial annular tear or to the vertebral body through the radial endplate tear, respectively [54]. This determination could be relevant, as it may give direction toward different treatment options aiming for different parts of the disc.

### 4.7 Diagnostic Accuracy

To date, diagnostic accuracy is an issue of debate. Several studies have shown a high false‐positive rate of provocative discography in certain populations (as high as 50%–60% in one study), particularly in those with preexisting psychiatric morbidities, failed back surgeries, and other co‐existing pain conditions [55, 56, 57, 58]. However, a systematic review and meta‐analysis performed by Wolfer et al. showed a far lower false‐positive rate of 9.3% per patient and 6% per disc; when those with preexisting chronic pain (including back pain, which is the indication for discography) and psychiatric morbidities were excluded, the false‐positive rate declined to 5.6% per patient and 3.85% per disc [59]. Wolfer et al. also concluded that testing control discs at adjacent segments and keeping the injection pressure low can minimize the false‐positive rate and consequently prevent unnecessary (minimally) invasive treatments. False‐positive results caused by stimulating potentially painful adjacent discs are less likely to occur when performing provocative discography with a low‐speed flow [60, 61, 62]. Before starting discography, it should be considered that pain generated from the SI joint and the lumbar facet joint most often mimics disc‐related complaints. Therefore, to improve the index of suspicion of discogenic pain, other causes (SI and facet, or other causes of mechanical low back pain) have to be ruled out with a test block before starting an invasive procedure like discography [1].

### 4.8 Complications of Discal Puncture

The most important complication of discal puncture is discitis, which typically manifests within 1 week of the procedure with an incidence of 0.25% [63, 64, 65]. *Staphylococcus aureus* is the main pathogenic organism. Discitis is difficult to treat because of the avascular nature of the disc. Therefore, prevention by aseptic procedures and preoperative administration of intravenous antibiotics seems important, although the routine use of antibiotics is debatable [64, 66]. Other rare complications include nerve root injury, (retroperitoneal) hemorrhage, meningitis, acute disc herniation, allergic reaction, dural puncture with concordant headache, cauda equina syndrome, and vertebral body osteonecrosis [64]. The acceleration of disc degeneration and a higher incidence of subsequent disc herniation have been described in several pre‐clinical studies [67, 68, 69, 70, 71, 72] and some clinical studies [73, 74, 75]. The incidence of these complications and discitis depends in part on needle diameter, with a higher risk when using large‐bore needles (18G and 21G) [66, 76, 77, 78]. However, using too small of a needle may increase the difficulty of disc puncture and increase the risk of the needle bending or even breaking [77]. Therefore, one should consider the “double‐needle technique” in which a larger introducer and guide needle is inserted through the skin until the annulus is reached, after which a smaller needle (25G) is advanced through the first needle into the disc. In addition to reducing the risk of disc injury and persistent pain, the double‐needle technique may also reduce the infectious risk since the needle entering the disc is not directly entering the skin [66]. In addition to a higher rate of false‐positive blocks, the use of higher injection pressures (e.g., ≥ 100 psi) appears to be associated with a greater risk of disc injury and disc herniation than the use of lower injection pressures (e.g., ≤ 50 psi) [79, 80, 81]. Taking the risk of discitis into consideration, discography should only be performed in patients with debilitating pain being considered for a procedural intervention in whom a discogenic origin is suspected but unproven.

### 4.9 Criteria

Considering the invasiveness and false‐positive rate of provocative discography as described above, standards for indications, technique, and interpretation should be followed.

#### 4.9.1 Patient Selection

Provocative discography should only be performed as a diagnostic tool if the patient meets all the following conditions:

1. Debilitating low back pain that remains unrelieved or uncontrolled after at least 3 months of conservative treatment (see following chapter “Treatment options”) and is not accompanied by obvious radicular pain extending to the lower extremities.
2. In clinical evaluations, as well as X‐ray and MRI examinations, there is evidence of disc degeneration, but the source of pain is unclear. Pre‐procedure imaging should ideally be conducted no more than 1 year prior to the procedure.
3. (Diagnostic) intra‐articular or posterior sacral network local anesthetic blocks for the SI joint pain, and intra‐articular blocks or medial branch blocks for facet joint pain are ineffective in relieving pain since pain reduction of 50% or more was not achieved.
4. The pain is severe enough that the patient wants (minimally) invasive treatment, and provocative discography is being used to guide decision‐making.

Absolute contra‐indications include pregnancy, suspected local or systemic infectious diseases, previously fused intervertebral body or transverse fusion at the same level, spinal cord, or cauda equina compression at the same level. Relative contra‐indications include coagulation disorders, use of anti‐coagulants, cardiovascular instability, a history of allergy to contrast agents and renal failure.

### 4.10 Procedure

#### 4.10.1 Preparation

Provocative discography is performed under strict sterile conditions. Thirty minutes before the intervention, the patient is administered intravenous antibiotics (2 g cephazolin, iv). Many interventionalists also mix antibiotics within the intradiscally injected contrast agent at a concentration between 1 and 10 mg/mL (e.g., 3 mg/mL cephazolin). In the operation room, the patient lies in the prone position on an X‐ray permeable table. In systematic reviews, the use of prophylactic antibiotics has not been shown to definitively decrease the risk of infection [66, 82], and their use should depend on a careful risk–benefit evaluation.

The skin of the low back and the gluteal region is thoroughly disinfected. After the injection point has been marked, the patient, as well as the C‐arm, is covered with a sterile drape. Due to the limited rotation of the C‐arm, the drape must be located on the side of the patient where the needle will be inserted.

The levels to be examined are chosen based on a combination of patient history, physical examination, and additional examinations. The symptomatic level(s) and up to two adjacent levels are examined, serving as control levels. Typically, the least degenerated or more likely asymptomatic levels are studied first. The patient should be blinded to the disc level and unaware of the start of the disc stimulation. Patients should not be overly sedated for the procedure, but those on high doses of opioids should be given opioids, or take their baseline opioids, so their pain sensitivity is not exaggerated. The patient must be awake and able to reliably report during the disc stimulation.

The C‐arm is first positioned with the direction of the radiation beam parallel to the subchondral plate of the lower vertebra to create an optimal view.

For discs above L5‐S1, the C‐arm is rotated ipsilaterally to align the anterolateral edge of the superior articular process with the mid‐portion of the disc and so that the disc height is at its maximum. In patients with a lateral disc collapse or scoliosis, adjustment of the C‐arm may be necessary.

At the L5‐S1 level, the iliac crest (especially in males) may obstruct access to the disc in a co‐axial view. The fluoroscopy tube is rotated until the lateral edge of S1 superior articular process is positioned approximately 25% over the posterior to anterior distance of the vertebral body. To facilitate needle placement in the NP at this level, curved (or bent) needles are sometimes used.

#### 4.10.2 Needle Positioning

A new sterile needle should be used for each disc examined. Needles should not be re‐used to decrease the risk of discitis. After anesthetizing the skin and underlying tissue with local anesthetic, a single‐ or double‐needle technique can be used to approach the disc. In the double‐needle technique, a 20G needle is advanced over the medial edge of the superior articular process. A 25G hollow needle is then inserted through this needle and into the AF until it reaches the center of the NP.

Then, the needle can be inserted parallel to the direction of the radiation beam and brought into position (tunnel view). The target for puncturing the AF is the lateral‐middle side of the disc, just lateral to the edge of the superior articular process.

**FIGURE 3:**
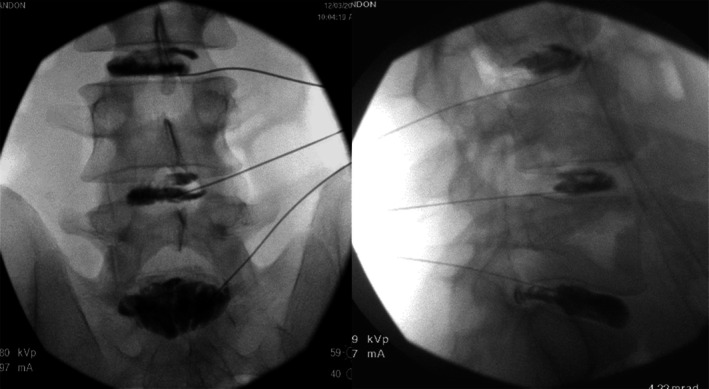
Discography at three levels: L3–4 and L4/5 normal discs and L5‐S1 with tear.

The small discography needle is carefully advanced to the end of the introducer. Beyond the superior articular process, the discography needle passes through the intervertebral foramen in the vicinity of the ventral ramus. In case of paresthesia, which is particularly common at L5‐S1, the needle must be repositioned. An increase in resistance may be appreciated as the needle passes through the annulus. The needle is pushed through the annulus to the center of the NP, confirmed in numerous views. Figure 3 shows the needle positioning during discography.

#### 4.10.3 Disc Stimulation

After verification of correct needle position, the stylet is removed, and the needle is connected to a contrast agent delivery system which can measure the intradiscal pressure (manometry). Check if the manometer reads zero pressure and zero volume. The contrast agent used can be iohexol, iodine, or gadolinium (in case of iodine allergy), though the risk of serious complications with inadvertent intrathecal administration has limited the use of the latter (contrast often diffuses epidurally during discography and there are connections in the form of arachnoid villi between the epidural and intrathecal space). At this point one can choose to see if there is a “placebo”‐response by telling the patient they might feel something or ask if they feel something different, while nothing is done.

The rate of infusion of the contrast agent should not exceed 0.05 mL/s [83, 84, 85]. This reflects a static flow rate that corresponds to the distension pressure in the IVD. If a higher flow is used, false‐positive discographies are more likely to occur due to the resultant pressure peaks. Pain is often provoked by pressure peaks due to VEP compression and distention of the adjacent facet joint.

Many experts agree that the disc expected to be most painful is stimulated last; the patient should not be aware of which disc is being stimulated. If the painful disc is stimulated first, it is possible that the provoked pain persists long enough to make adequate stimulation at other levels impossible. Once these conditions have been met, stimulation can begin.

During the injection of the contrast solution, the following parameters must be carefully monitored to assess the diagnostic criteria mentioned in Table 1:

- The opening pressure, or the pressure at which contrast is first observed to enter the disc;
- The provocation pressure, or the pressure greater than the opening pressure at which complaints of pain arise;
- The peak pressure or the final pressure at the end of the procedure;
- The type of endpoint reached, or what limits the final stimulation pressure (i.e., pain endpoint or an injection is halted because of pain; a pressure endpoint or increased resistance at a certain pressure; a volume endpoint, where the total volume is exceeded which may occur in a diffuse degenerated disc in which extravasation of contrast limits pressurization);
- The type of pain reproduction, often graded as no pain, non‐concordant pain, partially concordant pain, or fully concordant pain.

**TABLE 1:** Certainty of positive result for discogenic pain.

Certainty	At psi above opening pressure	Positive two adjacent discs
Absolute	< 15	None
Highly probable	< 15	One
Probable	< 50	None
Possible	< 50	One at psi > 50 and discordant

Ideally, the pressure, volume, and provocation details are recorded at 0.5 mL increments, with additional notations made for the aforementioned events. Furthermore, the morphology of the discs is graded according to the modified Dallas Classification, as described later in this chapter [86].

The procedure, per level, is continued until one of the following occurs [83]:

- Concordant pain is reproduced at a level exceeding its baseline level, and subsequently injected contrast confirms the response.
- The volume infused reaches 3.0 mL (up to 4 mL may be injected into a very degenerated disc when pressures remain < 15 pounds per square inch).
- The pressure rises to 50 pounds per square inch above opening pressure in discs with a grade 3 annular tear.
- If contrast agent leaks through the outer annulus or through the endplates, one may not be able to sufficiently pressurize the disc to assess disc sensitivity. In these cases, rapid manual injection may be acceptable but must be noted.

### 4.11 Postoperative Care

After discography, the patient is admitted to the recovery room. The patient is discharged home if the pain is managed and there are no signs of complications. The patient may experience worsening of pain symptoms in the first few postoperative days, which can be treated with analgesics. The patient should be instructed to contact the doctor immediately if she/he experiences an increase in symptoms, loss of neurological function, and/or fever.

### 4.12 Diagnostic Criteria

Regarding provocative discography, the International Association for the Study of Pain (IASP)/International Pain and Spine Intervention Society (IPSIS) describes the following criteria:

- The stimulation of the target disc is positive when it leads to concordant pain with an intensity exceeding its baseline pain score or being at least 70% of the maximum spontaneous pain.
- The certainty of this positive result is rated as described in Table 1.

Given that stringent selection may improve the outcomes of minimally invasive and surgical treatments, the goal must be to identify 1 or 2 discs with a high probability of being the primary source of pain. This way the risk of false‐positive results and unnecessary (minimally) invasive treatment is reduced. According to O'Neill & Kurgansky, when the threshold for pain provocation is above 50 psi, the probability of a false‐positive disc is almost 100%; at 25 psi, it is 50%; at 19 psi, it is 25%; and at 14 psi, it is reduced to 10% [83]. Subjects with concordant pain provoked between 0 and 10 psi and evidence of pathology on CT or plain discography are most likely to be true positives for discogenic pain.

**FIGURE 4:**
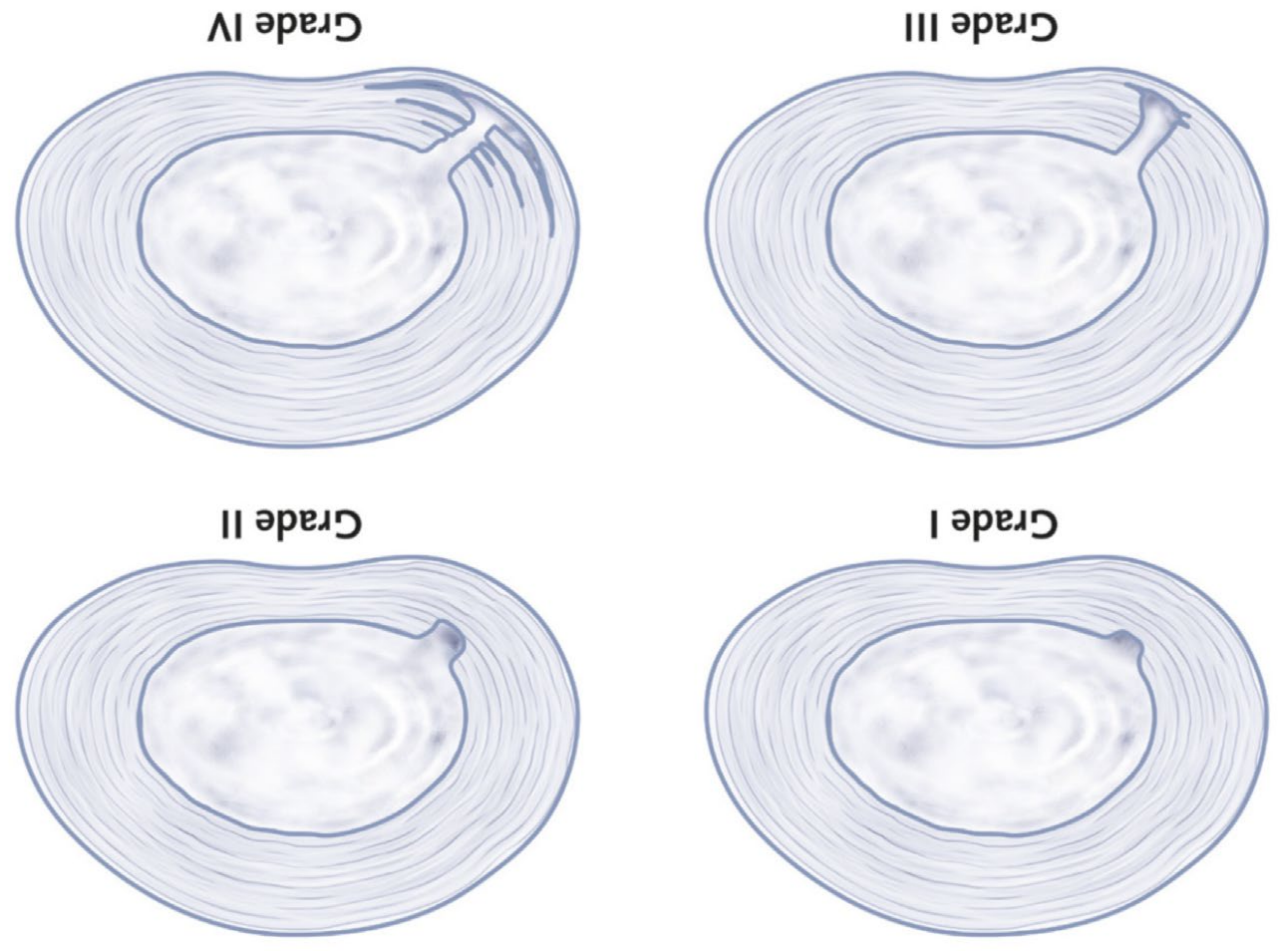
Gradation of the radial fissures visible on CT discography. Illustration: Rogier Trompert Medical Art. http://www.medical‐art.eu.

Provocative discography can be followed by a post‐discogram CT to identify the exact location and extent of annular disruption. Whether using the X‐ray with the C‐arm or CT, the discogram can be categorized using the modified Dallas Classification, describing the severity of radial annular tears (Figure 4) [86, 87]:

- Grade 0: the contrast remains entirely in the NP.
- Grade I: the contrast leaks into tears in the innermost 1/3 of the AF.
- Grade II: the contrast extends into tears in the middle 1/3 of the AF.
- Grade III: the contrast extends into tears in the outer 1/3 of the AF.
- Grade IV: full thickness (grade 3) radial tear with contrast also spreading concentrically/arc‐shaped around or in the innermost ring of the AF. The concentric diffusion must be > 30°.
- Grade V: the contrast leaks out of the disc. This type of tear may be associated with “chemical radiculopathy” where the disc content leaks toward and irritates the nerve root(s).

## 5 Treatment Options

Treatment options can roughly be divided into conservative treatment, minimally invasive treatment, and surgical treatment. This review focuses on minimally invasive treatments.

For managing CDLBP, one should consider its natural course. Peng et al. [88] prospectively followed 131 patients with CDLBP for 4 years. The improvement rates of both pain (7.6%) and disability (5.2%) were very low, and compared to baseline there was no significant change over the study course.

It is also important to consider the significant placebo effect of minimally invasive procedures in patients with chronic low back pain [89]. Therefore, conclusions should ideally be drawn based on sham‐ or placebo‐controlled clinical trials.

### 5.1 Conservative Treatment

Regarding conservative non‐pharmacological treatment of CDLBP, only a few studies have been published. A review by Adams et al. asserts that exercises, mobilization, and spinal loading and unloading may cause some degree of pain relief [25]. Traction therapy (delivered manually by a therapist or via gravity with the pelvis secured) is theorized to increase the nutrient flow to degenerated discs through cycles of distraction and relaxation, but its clinical effectiveness has not been demonstrated [90, 91]. No RCT was able to demonstrate a positive long‐term effect for the intervention, with only marginal differences observed between the groups. Case series have reported the highest degrees of improvement but are limited by the lack of a control group and high heterogeneity (variable time, number of sessions, weights used, and equipment).

In non‐specific chronic low back pain, various options for conservative treatment are available as indicated in multiple systematic reviews and meta‐analyses: exercise therapy [92] including motor control exercise [92, 93, 94], pilates [95, 96], McKenzie exercises [97, 98], and yoga [99, 100, 101], Virtual Reality [102], spinal manipulation therapy [103, 104] and psychological interventions (cognitive behavioral therapy, pain education programs, and mindfulness‐based stress reduction) [105, 106]. Multidisciplinary biopsychosocial rehabilitation—at least those containing a validated pain education program, structured exercise therapy, and psychological interventions as indicated—seems to be most favorable in non‐specific chronic low back pain [107, 108, 109, 110]. This multidisciplinary approach is in line with the definition of chronic pain by the International association of the Study of Pain (IASP): “a personal experience that is influenced to varying degrees by biological, psychological, and social factors”.

In conclusion, there are no known studies demonstrating the efficacy of conservative non‐pharmacological treatment for patients with CDLBP, so the interventions on non‐specific chronic low back pain mentioned above should be extrapolated to these patients.

#### 5.1.1 Pharmacological Management

Pharmacological management should be considered if the described conservative treatment options prove insufficient, or for pain exacerbations. Pain medications are generally recommended for limited periods of time to reduce the pain sufficiently to exercise again. There are no known studies demonstrating efficacy for any analgesic specifically in patients with CDLBP, so results of studies for non‐specific chronic back pain or other indications are extrapolated for specific back pain disorders. Nonsteroidal anti‐inflammatory drugs (NSAIDs) including cyclooxygenase‐2 inhibitors, are the main pharmacological agents used to treat chronic discogenic pain as they reduce inflammation and may be combined with other analgesic medications such as paracetamol/acetaminophen and “weak” opioid analgesics. Anti‐neuropathic medication, serotonin‐noradrenaline reuptake inhibitors (such as duloxetine), and tricyclic antidepressants can be considered as other pharmacological options [111, 112, 113]. If the pain is characterized by nociplastic mechanisms, the use of opioids is strongly discouraged as they may exacerbate central sensitization [114]. Corticosteroids and skeletal muscle relaxants such as benzodiazepines, dantrolene, tizanidine and baclofen are not recommended in chronic discogenic pain. There is no evidence to support the use of gabapentin or other membrane stabilizers and strong opioids, which carry the potential for significant side effects.

The use of oral antibiotics has been recommended in refractory cases. It is hypothesized that Modic type 1 changes occur as a result of inflammation secondary to a low‐grade infection of the disc [115]. The basis for this hypothesis rests in the fact that a high percentage of herniated and degenerated discs with type 1 Modic changes contain anaerobic bacteria on biopsy [116]. To test this hypothesis, Albert et al. conducted a double‐blind RCT in 126 patients with chronic low back pain and Modic type 1 changes at the level of a previous disc herniation and found that a 100‐day treatment with amoxicillin and clavulanic acid significantly improved pain and disability at 1‐year follow‐up [115]. Braten et al. reproduced this study with amoxicillin without clavulanic acid in patients with both Modic type 1 and 2 changes, finding similar results [117]. However, other investigations have failed to find efficacy for long‐term antibiotic use, with failures attributed to different indications, poor selection (i.e., discography was not used to select patients) different treatment regimens, and multifactorial mechanisms for disc degeneration. Given these issues and the side effects of long‐term administration, including the potential for antibiotic resistance, antibiotics should not be considered standard care but may be considered in certain circumstances.

### 5.2 Minimally Invasive Interventional Treatments

Various minimally invasive treatments have been used throughout the years to manage CDLBP such as intradiscal injections with multiple agents, various intradiscal thermal therapies and therapies targeting structures surrounding the disc, including spinal cord stimulation (SCS) and ramus communicans blocks. Despite the potential of minimally invasive treatments as effective alternatives to surgical intervention, their experimental status persists due to limited consensus and inconsistent evidence. However, interventions where the level of evidence is established (with at least prospective cohort trials) are outlined below.

#### 5.2.1 Intradiscal Injections

Intradiscal injection (IDI) is a minimally invasive technique with scarce or conflicting evidence that continues to be recommended in some guidelines despite the risks of disc puncture and the chondrotoxicity of some of the agents injected [118, 119].

#### 5.2.2 Intradiscal Corticosteroid Injections

The goal of intradiscal corticosteroid injections is to counteract the inflammatory effect that is considered responsible for discogenic pain.

##### 5.2.2.1 Evidence

Five double‐blind RCTs have been published on the use of intradiscal corticosteroids in chronic discogenic pain. In 1992, Simmons et al. published an RCT in which 25 patients with positive discography received either 80 mg methylprednisolone or 1.5 mL bupivacaine 0.5% intradiscally [120]. Neither group fared well, with no significant difference observed between groups, although the follow‐up period was only 2 weeks. In 2004, Khot et al. published a similar RCT comparing intradiscal methylprednisolone to steroid in 120 patients. Similar to Simmons et al. neither group did particularly well, though the only follow‐up time point was at 1 year, which is much longer than any expected benefit of steroids should last [121]. In 2011, Cao et al. conducted an RCT in 120 patients who had positive discography and endplate signal changes on MRI, showing that intradiscally administered steroids, with or without songmeile (a cervus and cucumis polypeptide) yielded clinically significant improvements in pain and function at 3 and 6 months post‐treatment compared to a control group, but no statistical significance was assessed [122]. In 2017, Nguyen et al. conducted an RCT in 120 patients with chronic low back pain and Modic type 1 endplate changes on MRI, without pre‐injection discographic confirmation [123]. During discography, patients received either 25 mg of prednisolone or no steroid. They found that the modest benefit in pain reduction (1.5 on the 0–10 Numeric Rating Scale (NRS)), but not secondary outcomes afforded by steroid, lasted only 1 month, though the only other follow‐up was at 12 months. In 2021, Tavares et al. showed the same short‐term efficacy of 50 mg prednisolone intradiscally compared with lidocaine in 39 patients with chronic low back pain and Modic type 1 changes on MRI, also without discographic confirmation of DP: 2.8 on the 0–10 Visual Analog Scale (VAS) at 1 month, but not at 3 and 6 months follow‐up [124]. Overall, the current literature shows conflicting results on effectiveness and provides no clarity on safety. Hence, the use of intradiscal steroids remains experimental.

#### 5.2.3 Methylene Blue Injection

Methylene blue is a low‐molecular weight, partially liposoluble dye that may provide pain relief through three pathways: anti‐inflammatory effects (i.e., by inhibition of free radical generation, deactivation of xanthine oxidase, and inhibition of the production of nitric oxide) [125, 126], sodium channel blockade and inhibition of neuronal excitability, and through denervation of nociceptive nerve endings [127]. It is therefore hypothesized that methylene blue injection (MBI) reduces CDLBP. MBI consists of 1 mL methylene blue (10 mg/mL), with or followed by 0.5 to 1 mL lidocaine (20 or 40 mg/mL) to alleviate procedure‐related pain [128, 129, 130, 131, 132, 133].

##### 5.2.3.1 Evidence

In 2021, Deng et al. [134] conducted a meta‐analysis of three RCTs, concluding that MBI exerts beneficial effects on pain reduction (mean difference (MD) 0.71 at 3 months and 13.92 at 6 months) and functional improvement (MD 3.66 at 3 months, but not at 6 months) in patients with CDLBP. However, it is important to note that one of the included RCTs employed an active control group (ozone ablation). Moreover, the two sham‐controlled RCTs yielded conflicting results: while one trial [131] reported a statistically significant and clinically meaningful reduction in pain and disability persisting up to 24 months compared to the sham group (approximately a 4.5 point reduction on the 0–10 NRS and a 33‐point reduction in the Oswestry Disability Index (ODI) on a 0–100 scale), a similar study [130] found no significant differences up to 6 months of follow‐up.

Expanding the scope to prospective, non‐controlled trials, a meta‐analysis by Guo et al. [135] in 2019 (*n* = 5 studies, 121 patients) demonstrated significant reductions in pain scores of 3.61, 2.95, and 3.19 on the VAS and significant ODI score reductions of 24.64, 23.21, and 29.51 at 3, 6, and 12 months, respectively, compared to baseline.

##### 5.2.3.2 Complications

There is theoretically a risk of methylene blue toxicity if the methylene blue leaks epidurally through annular tears and affects surrounding neural structures [127, 136]. Although clinical studies have thus far not reported this complication, extravasation into the epidural space is common [130, 131, 135]. Moreover, a recent in vitro study showed that MBI can induce apoptosis of the AF cells (in a concentration‐dependent matter) [137]. Given the time frame for induced disc degeneration and small numbers of patients, studies with short follow‐up periods would probably not be able to detect this.

##### 5.2.3.3 Considerations

The present research contains a low level of evidence that intradiscal methylene blue injection could be considered in a carefully selected patient group with refractory CDLBP. However, due to the conflicting results and potential for accelerated degeneration, we do not currently recommend this outside of a study setting.

#### 5.2.4 Ozone Discolysis and Ethanol Gel‐Based Chemonucleolysis

Ozone discolysis, involving the intradiscal injection of an ozone‐oxygen mixture, is hypothesized to reduce disc volume and enhance the endogenous anti‐inflammatory response. However, its primary application is in patients with spinal disc herniation [138, 139, 140, 141, 142]. The evidence supporting its use in patients with CDLBP is limited to small, low‐quality, and uncontrolled studies [143, 144, 145].

Ethanol gel‐based chemonucleolysis is proposed to reduce intradiscal pressure by inducing NP shrinkage and to facilitate denervation/denaturation of nerve endings within the disc. The available evidence is largely derived from a single prospective cohort study [146] involving 18 carefully selected patients, which reported significant pain reduction (5‐point decrease on the VAS), functional improvement (14‐point increase on the a 0–24 Roland‐Morris Disability Questionnaire scale), and enhanced quality of life (34‐point increase on the 0–100 EQ‐5D scale) at 12 months. Subsequently, Latka et al. [147] conducted another uncontrolled prospective study in a larger but less well‐defined patient cohort.

Due to the limited evidence regarding both efficacy and safety, ozone discolysis and ethanol gel‐based chemonucleolysis are rarely utilized in clinical practice.

#### 5.2.5 Regenerative Medicine: Mesenchymal Stem Cells and Platelet Rich Plasma

Recently, the intradiscal injection of regenerative biologicals—mesenchymal stem cells (MSC) and platelet‐rich plasma (PRP)—have been increasingly utilized. Whereas the current treatment options all focus on symptomatic pain relief, these therapies have the potential to not only reduce pain, but also to slow down degenerative processes and restore the structure of the disc (i.e., have the potential for disease modification) [148]. In these treatments, a set volume of MSC or PRP is injected directly into the degenerated disc(s). PRP is derived from centrifuged autologous whole blood and contains platelets, fibrin, and growth factors. After in vitro activation, the platelets have the ability to release platelet‐derived growth factors (PDGFs), including PDGF AA, BB and AB, resulting in a high concentration of growth factors [149, 150, 151, 152, 153]. This milieu is then thought to activate the remaining functional cells within the disc to proliferate and differentiate. The denouement of this sequence of events is supposed to enhance revascularization and downregulate matrix‐degrading enzymes, thereby restoring the extracellular matrix; to upregulate anti‐inflammatory and downregulate pro‐inflammatory mediators; and ultimately to preserve or restore the function and structure of degenerated discs [151, 154, 155, 156, 157, 158, 159, 160, 161, 162, 163].

MSC are undifferentiated multipotent cells that are able to self‐renew tissue in the disc by differentiating into mature NP cells (osteoblasts, adipocytes, and chondroblasts), secreting growth factors and cytokines to chemotactically activate the remaining functional cells, secreting anti‐inflammatory mediators to reduce inflammation and catabolism, and promoting the synthesis of proteoglycans and (type II) collagen to restore the extracellular matrix [164, 165, 166, 167, 168, 169, 170, 171, 172, 173, 174, 175].

MSC are derived from either bone marrow (iliac crest), adipose tissue, peripheral blood or umbilical cord and can be in vitro amplified, conditioned, pre‐differentiated and modified using different biological stimuli or scaffolds to acquire an NP cell phenotype before being injected into the damaged disc [174, 176, 177, 178, 179, 180, 181, 182, 183].

##### 5.2.5.1 Evidence

Multiple systematic reviews have been published regarding the effectiveness of injections of MSC and PRP for CDLBP [155, 184, 185, 186, 187, 188, 189, 190]. They generally conclude that there is a (very) low quality of evidence that MSC and PRP injections reduce pain (respectively and approximately 3.7 and 4 points on the VAS) and disability (respectively and approximately 26 and 13 point decrease in ODI) after 12 months. The (very) low‐quality evidence designation was based on one negative and one marginally positive RCTs [191, 192] and multiple observational studies [193, 194, 195, 196, 197] for MSC and two (negative) RCTs [152, 198] and multiple observational studies for PRP [199, 200, 201, 202, 203], all with heterogenous interventions, different ways of deriving and preparing the MSC and PRP, wide variations in reporting and outcome measures, poor patient selection (provocation discography was seldom used), and a high risk of bias. Most did not quantify or qualify (i.e., viability) of the products injected. There was negative evidence for the restoration of disc function and structure for both treatments. The short follow‐up times may also not be sufficient to generate conclusions about efficacy. The activation of residual functional cells can theoretically result in a reversal of disease progression, but this may take months or even years to realize [204, 205].

##### 5.2.5.2 Complications

Besides the generic complications of intradiscal injections (see sections *Diagnosis*, *Complications of discal puncture*), specific risks of biological treatments are aberrant growths such as hyperplastic gliosis, osteophyte formation, and malignancy, as well as an altered immune milieu resulting in infection/inflammation and immune rejection of autologous/allogenic biologicals [206, 207].

Whereas none of the patients in the reviewed studies developed such complications during their limited follow‐up, government regulators are concerned enough that they have mandated surveillance for these events, as there is still insufficient data to accurately assess safety.

##### 5.2.5.3 Considerations

Regarding the lack of high‐quality evidence and insufficient complication data, PRP and MSC should currently not be considered standard care in the treatment of chronic discogenic pain. Before PRP or MSC treatments can be recommended for clinical practice, future high‐quality clinical trials with longer follow‐up periods and more complete complication data should demonstrate efficacy and safety. In addition to methodological quality, studies should seek to determine the optimal source for stem cells, the optimal injectate composition (the density of fibrin networks and the presence of leukocytes in PRP/purity of PRP) dose, and refine injectate preparation (the phenotypic changes and the expression of cytokines and related genes). The ideal window (i.e., timing) for therapy is another unknown. Despite these limitations, the expected development of biological technology may result in a significant future role for cell‐based therapy in the treatment of CDLBP.

##### 5.2.5.4 Hypertonic Dextrose (Prolotherapy)

Prolotherapy has been widely introduced for various indications and is hypothesized to promote soft tissue regeneration. However, the evidence supporting its use in patients with CDLBP remains scarce and of low quality. In 2004, Derby et al. [208] prospectively followed 35 patients treated with a solution primarily composed of dextrose 50%, mixed with chondroitin sulfate, glucosamine hydrochloride, dimethyl sulfoxide (DMSO), bupivacaine, and a non‐ionic contrast agent. At an average follow‐up of 7.7 months, patients reported a mean pain reduction of 2.2 points. In 2006, Miller et al. [209] conducted a prospective study on 76 patients who received biweekly injections of a dextrose solution (25% dextrose and 0.125% bupivacaine) for up to five sessions. Among these patients, 33 (43.4%) demonstrated a sustained treatment response, with an average pain reduction of 71% at 18 months. To date, no RCTs have been conducted, and intradiscal hypertonic dextrose (or other forms of prolotherapy) should not be used outside of a study setting.

##### 5.2.5.5 Hydrogel‐Based Biomaterials

Hydrogels are biomaterial products that are intended to provide both physical stability to the NP and an ideal environment for MSC to exert their effects. They are hypothesized to become more viscous in response to increased temperature or acidity, reduce the likelihood of an adverse immune reaction against MSC, restore biomechanical properties, decrease inflammatory conditions, and reduce the risks of osteophyte and tumor formation by MSC [148, 210, 211, 212, 213]. Various hydrogels have been manufactured using natural or synthetic materials (e.g., alginate, gelatin, fibrin, collagen, chitosan, hyaluronic acid, silk, polyamides, and polyethylene glycol). Multiple pre‐clinical studies show promising results [206, 207, 210, 211, 212, 213], with 4 small clinical studies having been published thus far. In 2014, Yin et al. published a prospective uncontrolled study showing that the intradiscal injection of fibrin sealant improved pain scores and function at 6‐, 12‐, and 24‐month follow‐ups [214]. In 2017, Kumar et al. combined MSC with hyaluronic acid and demonstrated adequate safety, tolerability, and effectiveness at 12 months post‐treatment compared with conventional treatments [196]. In 2019, Ceylan et al. conducted a prospective cohort study in 29 patients with MRI‐diagnosed discogenic pain and found that GelStix resulted in a clinically relevant improvement in pain and function at 12 months [215]. In 2021, Amirdelfan et al. led a multicenter randomized study comparing 4 groups: (1) 18 million STRO‐3+ adult allogeneic mesenchymal precursor cells combined with hyaluronic acid; (2) 6 million mesenchymal precursor cells and hyaluronic acid; (3) a hyaluronic acid vehicle; (4) and a saline placebo injection. One hundred patients with a diagnosis of discogenic pain diagnosed by MRI, the exclusion of other sources of LBP with negative diagnostic injections, an absence of any full thickness annular tear on pre‐treatment disc injection, and, if needed, provocative discography, were included. The two treatment groups showed significant improvements in pain scores along with a lower need for post‐treatment interventions than the active and placebo‐control groups at various time points through 3‐year follow‐up [191]. Future research should clarify the effect of adding other biomaterials, such as hydrogel‐based products, to stem cells and stem cell precursors.

#### 5.2.6 Cytokine Antagonists

The inflammatory response plays an important role in fomenting extracellular matrix degradation and disc degeneration. The pro‐inflammatory cytokines tumor necrosis factor (TNF)‐α and interleukin (IL)‐1 and 6 are particularly highly expressed in degenerative discs and play an important role in the pathological process. Therefore, cytokine antagonists have been hypothesized to be a potential treatment target in slowing this cycle.

##### 5.2.6.1 Evidence

The clinical evidence regarding intradiscal cytokine antagonists in the treatment of CDLBP is scarce. A placebo‐controlled RCT by Cohen et al. [216] showed no evidence of efficacy to the TNF‐α antagonist etanercept, but this may have been because of the small study population size (*n* = 36) and the low dose of etanercept (escalating doses from 0.1 to 1.5 mg). A later RCT by Sainoh et al. [217] showed that the addition of 10 mg etanercept to 2 mL 0.5% bupivacaine reduced the pain scores and improved function scores at 4 weeks, though this effect diminished at 8 weeks. The same group showed in a similar set‐up that 40 mg of tocilizumab, an IL‐6 monoclonal antibody, showed a similar short‐term effect, separating from placebo for pain through 2 weeks and for function at 4 weeks post‐injection. At 8 weeks, the difference between groups was no longer significant [218].

##### 5.2.6.2 Complications

In the studies described above, only one patient (tociluzimab) had discitis. Theoretical complications such as immune‐related infections (leukopenia) and systemic inflammatory response syndrome (SIRS) were not observed at the low doses injected intradiscally.

##### 5.2.6.3 Considerations

Considering that only the study by Sainoh et al. demonstrated meaningful short‐term efficacy, the injection of intradiscal cytokine inhibitors should be limited for use in experimental settings [217, 218].

### 5.3 Intradiscal Thermal Procedures

Intradiscal procedures applying heat in an effort to ablate nociceptors have been collectively termed intradiscal thermal procedures. They apply electricity or radiofrequency (RF) energy to generate heat. Multiple procedural variants have been developed, with small differences between them. In the literature, many terms are used interchangeably for the same procedures. In this article, we describe the main ones: intradiscal electrothermal therapy (IDET), biacuplasty, Disctrode, pulsed radiofrequency (PRF), percutaneous intradiscal radiofrequency thermocoagulation (PIRFT), and nucleoplasty.

#### 5.3.1 Intradiscal Electrothermal Therapy

In 2000, Saal and Saal published the first use of IDET for patients with CDLBP [219]. IDET is a minimally invasive procedure in which heat (90°C) is applied to the AF through a percutaneous thermocoil, inserted and placed under radiographic examination at the anterolateral annular‐nuclear interface. It is a form of annuloplasty.

The working mechanism is not yet clarified. Some proposed hypotheses involve the denervation of the (upregulated) nociceptors in the AF resulting in less pain transmission, modification of the collagen fibers resulting in a reduction or sealing of annular fissures and stiffening of the segment(s), dehydration and stabilization of the disc, a decrease of vascularity of the AF, and possibly retraction of small contained herniations [219, 220, 221, 222, 223].

##### 5.3.1.1 Indication

Strict criteria have been proposed for treatment with IDET: more than 3 months (predominantly) low back pain, failing conservative treatment for at least 6 weeks (see conservative treatment section) with an MRI showing a maximum of 2 desiccated discs with at least 50% remaining disc height without large or uncontained herniations (< 3–4 mm protrusions) and positive provocation discography showing annular disruption [223, 224, 225]. The fact that no more than 2 discs are degenerative is important because the outcomes in cases of more extensive disc degeneration are significantly worse [225]. Absolute contra‐indications include spinal stenosis, spinal infections, uncorrected coagulopathy, and spinal instability (such as spondylolisthesis). Proposed relative contra‐indications are non‐specific inflammatory arthritis, obesity, previous lumbar spine surgery or IDET in the last 6 months [226, 227, 228].

##### 5.3.1.2 Technique

**FIGURE 5:**
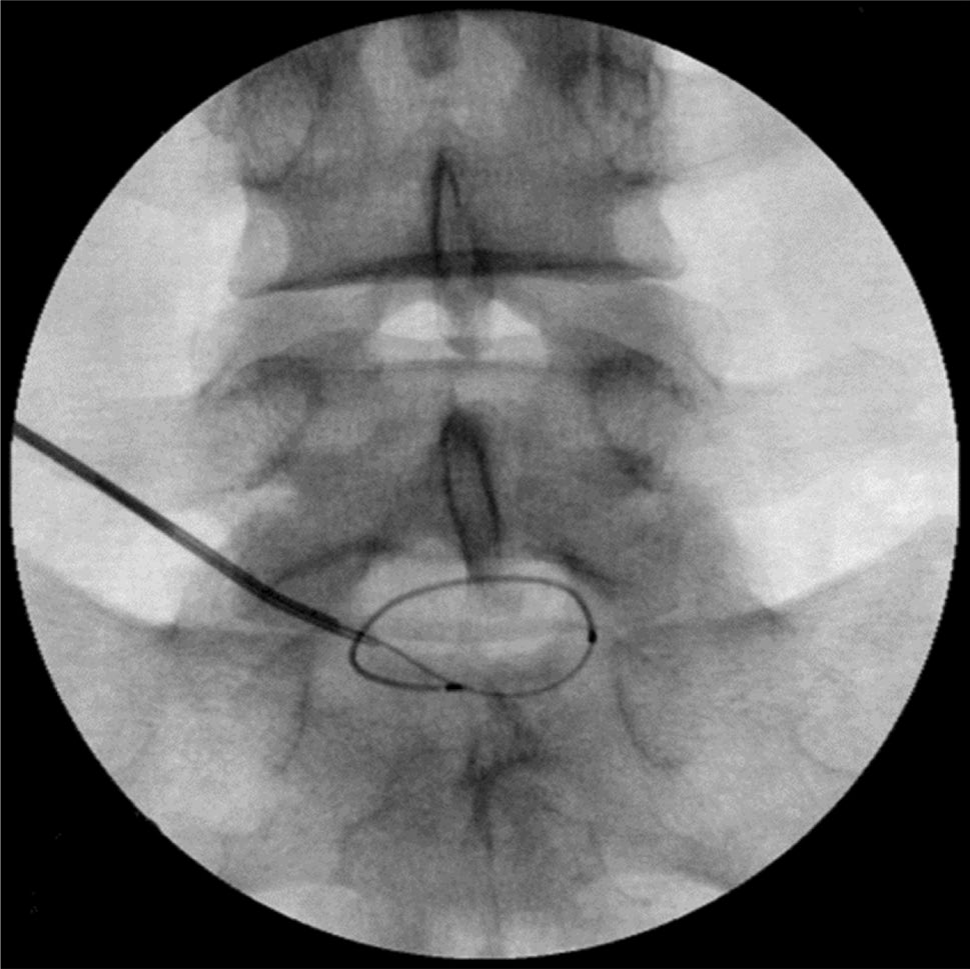
Intradiscal electrothermal therapy at level L5‐S1 in anterior–posterior view.

The procedure takes place under sterile OR conditions with the patient lying prone with the aid of radiographic examination using biplanar or C‐arm fluoroscopy. A hollow 17G introducer needle is inserted via the safe triangle located at the anterior border of the ascending facet and then posterolaterally into the disc, generally on the side with the least complaints. Thereafter, a 30‐cm long catheter with a 5 (1.5–6) cm long flexible tip [226, 229], which can be heated, is advanced through the needle. This tip is advanced through the NP and circumferentially around the inner annulus until it ideally covers the entire posterior section of the annulus (Figure 5). In patients with a large tear in the annulus, it may be impossible to maneuver the catheter into the correct position. After coil placement has been checked radiographically, the introducer needle is withdrawn and the catheter is fixed and connected to the IDET generator; the active tip is heated to 90°C to conform with the standardized radiofrequency ablation (RFA) protocol. This temperature is reached after approximately 14 (12.5–14) minutes and is maintained for 4 min at this level if the pain is bearable [226, 229]. Afterward, the needle and the catheter are removed after the temperature in the needle decreases.

If the patient reports (radicular) leg pain during the procedure, it is possible that a spinal nerve is being irritated and the heating process should be terminated immediately.

Prophylactic antibiotics (either intravenously or intradiscally) should be considered on a case‐to‐case basis in high‐risk individuals to prevent (spondylo)discitis [66, 82, 229].

##### 5.3.1.3 Postoperative

The patient can be discharged after an adequate recovery period. After the procedure, the patient must follow a strict 12‐week‐long rehabilitation protocol, including physical therapy. In the first month, the patient should only walk or lie down and do stretches of the hamstring, gastrocnemius, and soleus. During the first 2–3 months, patients extend the intensity and length of their activities and progressively perform increasingly rigorous stabilization floor exercises but still avoid lifting, bending, and prolonged sitting. After 5–6 months, patients are able to return to sports such as skiing, running, or tennis [219, 229].

##### 5.3.1.4 Evidence

Two moderate‐to‐high‐quality older RCTs have been published so far. In recent years, no new RCTs have been published. Pauza et al. screened 1360 patients with low back pain. After applying strict inclusion criteria, 37 patients underwent IDET and 27 patients sham therapy, in which the IDET catheter was also inserted but without applying the RF current. After 6‐month follow‐up, the improvement in pain and function scores was significantly higher, although the clinical relevance of the difference is debatable (1.3 point difference in VAS pain reduction and 7‐point difference in ODI score reduction). The proportion of patients in the treated group who improved by at least 50% in pain scores was only 40% versus 33% in the control group, for a number needed to treat of 5 [230]. Interestingly, stratified analysis suggested that patients with poor physical function and high disability were more likely to benefit from IDET, though the study population was relatively less disabled. Limitations are the small sample size, baseline differences in important variables, and a high lost‐to‐follow‐up rate. In 2005, Freeman et al. [231] published an RCT in 57 patients with greater disability than in the study by Pauza et al. [230]. After 6 months, no significant differences between IDET (*n* = 38) and sham therapy (*n* = 19) were observed for any outcome measure [231]. The main limitations are the small sample size and the absence of a placebo response suggesting ineffective blinding.

In 2006, two meta‐analyses were published showing contradictory results. Appleby et al. [232] analyzing seventeen prospective and retrospective cohort studies found a significant mean improvement in pain of 2.9 on the 0–10 VAS and 7 points on the ODI and asserted there was sufficient evidence of “relative effectiveness”. Freeman et al. [233] included eleven prospective cohort studies and reported a significant mean improvement in pain of 3.4 on the VAS and 5.2 points on the ODI, but concluded (while also considering the two described RCTs and other retrospective studies) that the current published evidence does not provide clear evidence of benefit. Furthermore, a more recent systematic review by Helm et al. [234] concluded that more recent observational trials are not of sufficient quality and that based on the described RCTs, the evidence for the use of IDET is level III (fair).

Andersson et al. [227] performed a systematic review comparing IDET with spinal fusion in 2006. They concluded that pain, spinal function, and quality of life improved comparably between the 2 procedures, but spinal fusion resulted in more complications. Since no comparative studies have been published, this systematic review was based only on cohort studies investigating one of the interventions, with much different selection criteria and outcome endpoints. As such, the quality of this evidence is very low.

##### 5.3.1.5 Complications

IDET technically requires large‐bore needles (in contrast to the needles used in discography) with a potential greater risk of complications (see “Complications of discal puncture” in chapter Diagnosis).

However, IDET procedure‐related complications rarely arise and include [226, 227, 232, 233, 234, 235]:

- Neural damage such as radiculopathy and cauda equina syndrome
- Accelerated disc degeneration
- Endplate deformation
- Infection, that is: (spondylo)discitis and epidural abscess
- Dural puncture route may result in post‐procedural headache
- Vertebral osteonecrosis
- Breakage of the catheter within the disc
- Disc herniation

##### 5.3.1.6 Considerations

In conclusion, one weakly positive RCT, one negative RCT, multiple positive and two negative non‐randomized prospective studies have been published to date, all over 15 years ago. Based on the limited database, the evidence of IDET is low to moderate, and we cannot recommend the routine use of IDET. One confounder with the various IDET studies is that the selection criteria are inconsistent, making generalization difficult. Larger, high‐quality studies with internationally defined inclusion criteria are needed to arrive at definitive judgments about the clinical effectiveness of the IDET procedure.

#### 5.3.2 Biacuplasty or Cooled Radiofrequency

Biacuplasty was first described in 2006 and is a form of intradiscal radiofrequency thermocoagulation (IDRT). The principle is simple: electricity is transmitted between two RF probes, creating a thermal lesion through the posterior annulus of the IVD. The thermistor temperature is raised gradually up to 50°C or 55°C [226, 235]. Biacuplasty is also referred to as cooled RF because the probes themselves are internally cooled. The proposed mechanism is the same as in IDET: the ablation of nociceptive nerve endings. In biacuplasty, there is minimal disruption to the native tissue architecture (due to the lower temperature) with a larger area that receives the energy and thus the biomechanics of the spine are likely unchanged [234]. Compared to IDET, there is also a lower technical failure rate as in many cases the IDET catheter cannot be circumferentially positioned around the posterior annulus. Thus, the relative ease of electrode placement into the posterior annulus eliminates the need to thread a long heating catheter (e.g., IDET).

##### 5.3.2.1 Technique

**FIGURE 6:**
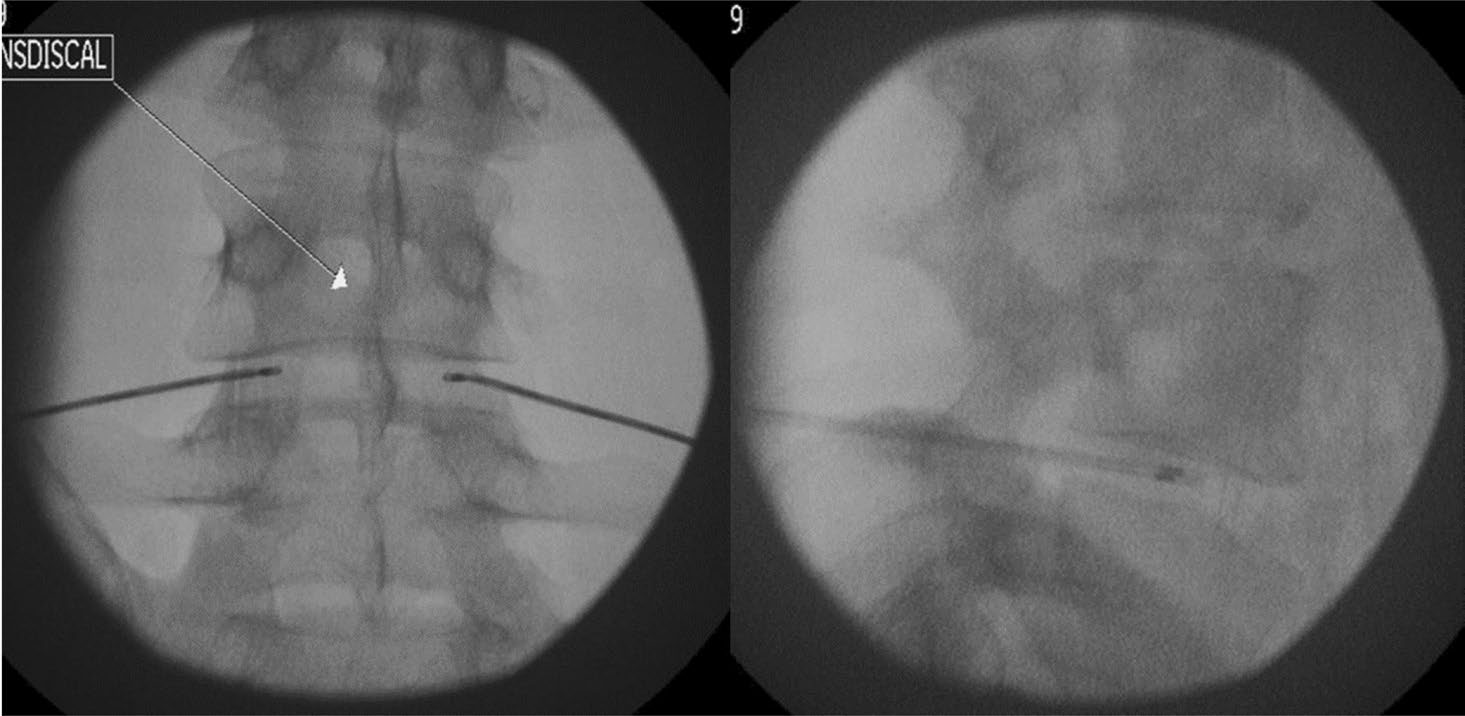
Biacuplasty at level L4‐L5 in anterior–posterior and lateral view.

The procedure is performed in a prone position, with the patient awake. Two TransDiscal 17G electrodes are placed bilaterally through introducers into the posterior annulus, using a posterolateral, oblique approach under fluoroscopic guidance (Figure 6). The temperature increases gradually over a period of 7–8 min to 50°C, maintaining this temperature for 7 min. An alternative method uses 18G electrodes, increasing the temperature over 11 min to 55°C and maintaining it for 7 min. It should be noted that although the temperature is set to 50°C on the RF generator, similar to IDET, the tissue temperature is higher, reaching 65°C due to ionic heating. After the needle is removed, the patient is observed for 45 min and then discharged [236].

##### 5.3.2.2 Evidence

In 2013, Kapural et al. [237] published a high‐quality RCT in 59 patients. At 6‐month follow‐up, physical function, pain, and disability were significantly improved in the biacuplasty group compared to the sham group. At 6 months, pain reduction and improvement in disability in the biacuplasty group exceeded that in the sham group significantly (respectively 2.19 vs. 0.6 points on the 10 point NRS and 7.43 vs. −0.24 points in ODI scores). This RCT excluded obese (BMI > 30) patients and smokers. Since obesity and smoking are known risk factors for IVD degeneration, this subset of patients may have been missed, though obesity appears to be a risk factor for complications after intradiscal heating [228, 238, 239, 240, 241]. In 2015, Kapural et al. [242] reported follow‐up data on the original 22 patients in the biacuplasty group through 12 months. In this cohort, the improvement in pain and physical function remained significant: NRS reduction of 2.73, ODI reduction of 7.93 and SF‐36 improvement of 21.82 points, compared to baseline.

Another RCT compared biacuplasty and conventional medical management (CMM) to CMM‐alone in 63 subjects [243]. At 6 months, VAS pain reduction in the biacuplasty + CMM group exceeded that in the CMM group significantly (2.4 vs. 0.56 points on the VAS), and the proportion of patients who experienced at least a 2‐point or 30% decrease in their VAS pain score was substantially greater (50% vs. 18%). Forty‐two percent of subjects in the biacuplasty + CMM group reported ≥ 50% relief of symptoms compared to only 7% in the CMM‐alone group. Functional outcomes also favored the biacuplasty + CMM group, including a mean difference in ODI scores of 8 points and a mean difference in SF‐36 of 12 points at 6 months.

Similar to Kapural et al. [242] Desai et al. [244] also reported a 12‐month follow‐up data on 22 of the original 29 patients in the intervention group. In this cohort, they found that the reduction in pain compared to baseline was 2.4 at 6 months and remained nearly unchanged at 2.2 at 12 months. Furthermore, a higher proportion of patients (55%) experienced at least a 2‐point or 30% decrease in VAS pain scores, while the percentage of patients achieving at least 50% improvement remained steady at 41%. Significant improvements were also noted in terms of function and disability when compared to baseline measurements: ODI reduction of 12 and SF‐36 improvement of 14 points compared to baseline. However, due to attrition in the CMM‐only group, the study lacked the power to compare outcomes between the two groups.

In both follow‐up studies, the control group was offered biacuplasty after unblinding. A significant majority, 89% and 80%, respectively, opted for crossover and demonstrated clinically meaningful improvements at the final follow‐up similar to patients initially randomized to biacuplasty.

Elawady et al. [245] conducted another RCT comparing biacuplasty with conservative management in 44 patients with CDLBP, diagnosed by clinical findings and MRI. At follow‐ups up to 6 months, the mean differences in NRS and ODI were significantly lower in the biacuplasty group, with clinically relevant reduction in mean NRS (5 vs. 2.4 points) and ODI scores (22.5 vs. 10.1 points) at 6 months.

##### 5.3.2.3 Complications

The safety profile of the procedure and the absence of perioperative and postoperative complications have been reported [234, 242, 246]. The published literature regarding biacuplasty has not reported any complications, and thus the procedure should be considered low risk for adverse events. However, the generic complications for disc‐heating procedures (e.g., the potential for subsequent disc injury, neurological complications from cannula placement and heating, see “IDET”) can occur, and the paucity of complication data regarding this procedure warrants caution.

##### 5.3.2.4 Considerations

Based upon evidence from three high‐quality RCTs, biacuplasty seems to provide a clinically relevant reduction in CDLBP and commensurate functional improvement. However, more RCTs with longer follow‐up periods are needed to confirm these findings as well as the safety. Based on dated literature, biacuplasty may be considered as a treatment option for patients with discogenic back pain refractory to other treatments.

#### 5.3.3 Disctrode

Another described RF technique is Disctrode. It functions by utilizing a wire placed across the posterior annulus to generate a unipolar RF lesion. In a sense, it is a unipolar variant of biacuplasty. Disctrode does not solve the technical challenges involved in passing a wire across a diseased annulus. The evidence supporting Disctrode is very limited. One case–control study reported added benefit over stand‐alone conservative treatment in patients who either refused the treatment or could not get the procedure paid for by insurance, but did not include a sham control [247]. Kvarstein et al. published an RCT comparing Disctrode with a sham treatment [248]. Although the study had only 20 patients, there was no benefit observed for Disctrode, with increased pain reported in the Disctrode group. Another prospective matched‐control trial found that Disctrode was ineffective compared to IDET [225]. A systematic review including these studies concluded that there was no (or limited evidence) supporting the Disctrode and that it should not be considered standard of care [234]. Although they found no published cases of complications, the potential complications are similar to those of other disc‐heating devices.

#### 5.3.4 Percutaneous Intradiscal Radiofrequency Thermocoagulation

Percutaneous intradiscal radiofrequency thermocoagulation (PIRFT) uses RF energy to heat the disc in the center/nucleus and is hypothesized to coagulate collagen fibers, repair annular fissures, and ablate nociceptive nerve endings.

##### 5.3.4.1 Technique

Under fluoroscopic guidance, a 20‐G 15 cm cannula with a 10 mm active tip is directed into the NP and after negative motor (2 Hz) and sensory (50 Hz) stimulation, an alternating current (250–500 kHz) is produced by an RF generator. This causes ionic movement and heat generation in the area close to the active tip. The temperature of the active tip is continuously measured and when it rises to 70°C–90°C, it is maintained from 90 to 360 s [249, 250, 251, 252].

##### 5.3.4.2 Evidence

Clinical effectiveness of the PIRFT procedure has been reported in two RCTs. In a randomized, double‐blind, sham‐controlled trial by Barendse et al. that included 28 patients, no differences were reported for pain, global perceived effect, and ODI between the two groups 8 weeks after treatment [250]. It should be noted that this study was underpowered, and a diagnosis of discogenic pain was made by ruling out facetogenic pain followed by analgesic discography. In an RCT by Ercelen et al. 39 patients selected by provocative discography were randomized to either PIRFT for 120 s or 360 s. Similar to the study by Barendse et al. the authors found no significant differences in pain reduction and functionality between groups. Although improvement in both groups was noted at 1 month, at 6 months there was no meaningful benefit in either group compared to baseline [252].

Sun et al. performed a retrospective analysis comparing PIRFT with nucleoplasty (later described in this article) in 85 patients with CDLBP confirmed by provocative discography. They reported better pain reduction and functional improvement in the nucleoplasty group compared to the PIRFT group at 6‐ and 12‐month follow‐ups. In the PIRFT group, they reported 3.00, 2.50 and 2.30 points pain reduction on the VAS and at, respectively, 3, 6, and 12 months [251].

##### 5.3.4.3 Complications

Sun et al. reported an increased intensity of low back pain in three patients and new and persistent numbness in the leg and foot in two other patients [251]. No complications or side effects were mentioned in other studies, but it must be noted that the numbers of patients in these studies were small [249, 250, 252].

##### 5.3.4.4 Considerations

As discussed in the pathophysiology section, discogenic pain primarily originates from nociceptors located predominantly in the annulus's outermost layer. Thus, targeting the center of the nucleus with heat may not effectively reach and neutralize the intended nociceptors. Moreover, the current literature lacks comprehensive data on potential complications or side effects associated with PIRFT, suggesting a cautious approach to its clinical application. Although a study by Zhang et al. [253] suggests the possibility of improved outcomes with bipolar over monopolar PIRFT, the evidence is still lacking. More research on bipolar PIRFT is essential to better ascertain its efficacy and safety.

#### 5.3.5 Pulsed Radiofrequency of the Nucleus Pulposus

Pulsed radiofrequency (PRF) creates an electric field by brief electrical stimulations followed by longer resting phases. This electrical field results in a temperature increase up to 42°C, without damaging neural or tissue structures. In CDLBP, PRF of the NP is hypothesized to provide benefit by: (1) creating an electric field which reaches and halts conduction in nerve endings residing in the AF because of the low impedance of the NP; (2) creating heat that reaches the AF due to the thermal isolating properties of the VE; (3) enhancing descending modulatory pathways and favorably altering gene expression. The heat and electric field created modify nociceptors, reduce the conductivity of nerve endings, increase and decrease the production of anti‐ and pro‐inflammatory cytokines, respectively, thus reducing inflammation and pain [254, 255, 256]. Furthermore, PRF may affect the composition of the NP itself and thus reduce the triggering of nerve endings in the AF [254].

##### 5.3.5.1 Technique

In CDLBP, PRF is applied intradiscally. In the center of the NP, PRF is applied for 15–20 min (pulse width 5‐20 ms, 2–5 Hz, 60 V) through a 20‐G electrode with a large active tip.

##### 5.3.5.2 Evidence

The evidence regarding intradiscal PRF is of (very) low quality. Until 2014, three prospective single‐arm studies showed promising results at 6 or 12 months follow‐up, but no placebo‐controlled RCTs followed [255, 256, 257]. Fukui et al. retrospectively compared intradiscal PRF with IDET and found a small, but not statistically significant difference favoring IDET [255]. Papadopoulos et al. conducted an RCT to evaluate the additional value of PRF directly after intradiscal treatment with jellified ethanol and showed that the combined treatment resulted in significantly greater pain reduction at 6 and 12 months follow‐ups [258].

##### 5.3.5.3 Complications

No significant adverse effects were reported in these studies.

##### 5.3.5.4 Consideration

Regarding this evidence, intradiscal PRF should not be performed as a standard treatment in chronic discogenic lumbar pain, with randomized placebo‐controlled trials needed to elucidate its effectiveness.

#### 5.3.6 Nucleoplasty

This percutaneous minimally invasive technique is known by several names, including radiofrequency coblation, plasma discectomy, or plasma disc decompression. The procedure employs a process called “coblation,” wherein a bipolar RF probe generates a high‐energy plasma field. This field has the capability to disrupt molecular bonds, allowing for the evaporation of tissue at relatively low temperatures. Consequently, intradiscal pressure is reduced while maintaining the integrity of surrounding tissues. However, it is important to note that the effectiveness of this plasma field is contingent upon a conductive environment; thus, the procedure may not be suitable for dehydrated discs that have lost more than 50% of their height, often referred to as “black discs” on MRI scans. Additionally, contra‐indications for this treatment include conditions such as spinal stenosis, spinal fractures, or tumors.

##### 5.3.6.1 Technique

After positioning the patient prone, a 16‐ or 17‐G needle is positioned in the nucleus fluoroscopically guided via posterolateral approach. The probe is moved back and forth and rotated intradiscally, generating a temperature of 40°C–70°C. In this way, six or more tunnels are made in the nucleus and approximately 1 cm of the NP is removed. As the needle is withdrawn, coagulation takes place thermally treating channels and adjacent nerve fibers.

##### 5.3.6.2 Evidence

It has been suggested that nucleoplasty and plasma disc decompression techniques are best suited for patients with radicular pain secondary to contained disc herniations with protrusion sizes up to one‐third the sagittal diameter of the spinal canal, or ≤ 5 mm [259, 260]. However, for CDLBP, the evidence is limited. Four prospective observational studies have reported favorable clinical outcomes [261, 262, 263, 264]. The mean reduction in VAS was approximately 3 until a follow‐up of 12 months [262, 263]. There was 50% or more pain relief in 53%–74% of the patients until a follow‐up of 12 months [261, 262, 264]. The mean reduction in ODI score was 25 and 21, and the mean increment in the SF‐36 score was 14 and 12, at, respectively, 6 and 12 months [263]. One study reported results on 16 patients with radicular symptoms secondary to a contained disc and radiculopathy; in 7 with sitting intolerance and a positive discogram, IDET was also performed at the same visit. In these 16 patients, only 1 (who underwent only nucleoplasty) experienced at least 50% pain relief at 6‐month follow‐up [265]. Because of the limited evidence for effectiveness, placebo‐controlled trials are still needed.

##### 5.3.6.3 Complications

This treatment has been utilized on a large scale, and the complication rate appears to be low and acceptable [259, 260]. The most significant side effect is temporary soreness at the point of needle insertion [266].

##### 5.3.6.4 Consideration

Although some cohort trials report favorable outcomes, randomized placebo‐controlled trials are lacking. Therefore, this intervention should only be performed in the context of research.

### 5.4 Extradiscal Invasive Treatment Options

#### 5.4.1 Ramus Communicans Block

Except for the posterior disc, the sensory innervation of the disc is through rami communicantes and sympathetic trunk. A therapeutic approach might therefore involve RFA of the rami communicantes of the affected and contiguous disc(s). RFA causes denervation due to coagulation necrosis of neural tissue, thereby interrupting neural pathways.

#### 5.4.2 Diagnostic Block

The C‐arm is positioned such that the direction of the radiation beam in the transverse plane is approximately 20° oblique with the facet joints projected away, and the vertebral column clearly visible. For the angle in the sagittal plane, the C‐arm is rotated laterally on its axis. As a result, the transverse process changes location relative to the vertebral body. The direction of the radiation beam must be such that the axis of the transverse process lies slightly above the middle of the vertebral body.

Usually, an SMK‐C15 cannula is used for this procedure. The injection point is marked just caudally to the transverse process and somewhat medially to the lateral edge of the vertebral body. After local anesthetization of the skin, the needle is advanced in a co‐axial view until contact is made with the vertebral body. On the lateral projection, the point of the needle lies somewhat ventral to the posterior side of the vertebral body.

Half a milliliter of contrast agent is injected. In an anteroposterior projection, this usually results in a very compact shadow; on the lateral projection, the contrast agent spreads anteriorly over the vertebral body. In case of intravascular uptake, a small change in position is necessary. Finally, 1 mL of a local anesthetic is injected.

#### 5.4.3 Radiofrequency Ablation

**FIGURE 7:**
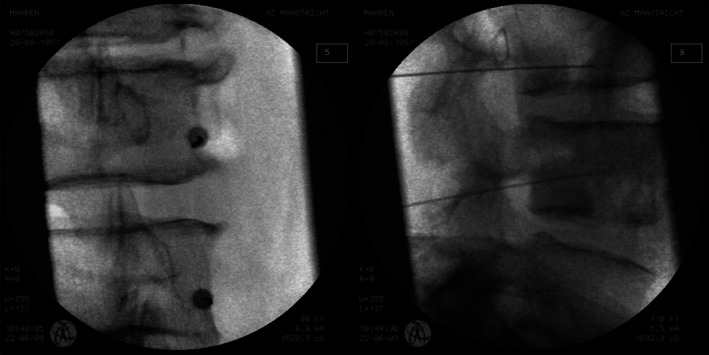
Radiofrequency ablation of the rami communicantes at levels L3–L4 and L4–L5 in lateral view.

An SMK‐C15 cannula with a 5 mm active point is used. The insertion of the needle for RFA should be the same or similar as that described for the diagnostic block (Figure 7). When the needle is correctly positioned, stimulation at 50 Hz should cause sensations in the back at a voltage of ≤ 0.5 V. Thereafter, 2 Hz stimulation is applied. Contractions of the leg muscles should not occur at below twice the value of the sensory threshold, or < 1.5 V. If these conditions are not met, then the needle is adjusted slightly until concordant stimulation without distal leg contractions has been achieved. An RF treatment is made for 120 s at 80°C.

##### 5.4.3.1 The L5 Level

This level deserves special mention since the anatomical relationships can require a modified approach. This can stem from a high iliac crest or a broad transverse process. In these cases, the L5 segmental nerve exits the intervertebral foramen more horizontally than the other lumbar nerves do. While adjusting the C‐arm axially, it is best to project the transverse process as high as possible. By doing so, a safe needle position can often be found for this level. Nevertheless, the intervention at this level may not be possible in all cases.

##### 5.4.3.2 Evidence

In 2004, Oh and Shim published a randomized sham (with lidocaine)‐controlled trial in 49 patients with CDLBP confirmed by provocative discography and limited to a single level. These patients had failed IDET and experienced ≥ 50% pain relief after a diagnostic ramus communicans block [11]. At 4‐month follow‐up, the patients treated with RF (60 s at 65°C) experienced greater reduction in VAS pain scores (3.3 vs. 0.7) and improvement in function (15.2 vs. 2.4 on the 36‐Item Short‐Form Health Survey) compared to the control group.

In 2016, Tilburg et al. conducted a non‐inferiority randomized sham‐controlled, double‐blind trial using RF during 60 s at 80°C and found no significant difference between groups at 3‐month follow‐up [12]. They included 60 patients with chronic discogenic pain, diagnosed by medical history, physical examination, a CT or MRI as indicated, and a positive diagnostic block. However, provocative discography was not used, which is an important methodological pitfall.

##### 5.4.3.3 Complications

The possible complications include mild to severe neuritis, numbness, and transient dysesthesia and weakness in the affected limb caused by thermal damage of the nerve root. Only the RCT by Oh and Shim mentioned possible complications, describing one patient with transient complaints of mild lower limb dysesthesia and weakness [11].

##### 5.4.3.4 Consideration

In conclusion, the current evidence is based on one positive but methodologically limited RCT and one negative RCT that did not use diagnostic provocative discography, both with limited follow‐up periods. More high‐quality RCTs with longer follow‐up are needed to better determine whether ablation of the ramus communicans can provide meaningful benefit and provide more information on complications. Therefore, the use of RF of the ramus communicans should be reserved for research studies.

#### 5.4.4 Radiofrequency Ablation of the Sinuvertebral and Basivertebral Nerves

RFA of the sinuvertebral nerve (SVN) and RFA of the basivertebral nerve (BVN) are relatively new techniques to denervate the disc. The BVN is a paired nerve arising from the SVN, which enters the vertebral body posteriorly and branches out into it, innervating it, including the VEPs.

##### 5.4.4.1 Technique

RFA of the BVN is performed in the prone position under general anesthesia or conscious sedation. With fluoroscopic or CT guidance in a true anterior–posterior view, an introducer trocar is placed transpedicularly, and with the help of a mallet, reaches and breaches the posterior aspect of the vertebral body, through which a curved bipolar RFA electrode is placed. RFA is performed at 85°C for 15 min. Sayed et al. [267] and Khalil et al. [268] described the procedure in detail for further reference.

RFA of the SVN is described by Kim et al. [269] with endoscopic visualization using a transforaminal or interlaminar approach. However, no clear RFA method was described.

##### 5.4.4.2 Evidence

RFA of the BVN have been investigated in patients with chronic vertebrogenic low back pain (CVLBP), but scarcely in patients with CDLBP. CVLBP is not clearly defined, but recent literature seems to agree that it is caused by endplate injury, resulting in chronic inflammation, high bone turnover and fatty infiltrates. The symptoms consist of predominantly (para)midline low back and buttock pain without lower leg symptoms, and Modic changes types 1 and 2 [270]. It is difficult to discriminate CVLBP and CDLBP, and it is not yet clear whether positive provocative discography rules out CVLBP [271]. Thus, it is plausible that these seemingly distinct pathologies overlap with similar nociceptive pathways.

Therefore, interventions described for CVLBP might be effective in patients with CDLBP. Several (systematic) reviews [272, 273] describe moderate‐quality evidence (multiple RCTs with multiple years follow‐up) that RFA of the BVN reduces pain and disability and improves function significantly in patients with CVLBP.

In 2021, Kim et al. published a prospective case series describing 30 patients with CDLBP (confirmed by provocative discography) and spinal stenosis or disc protrusion who underwent RFA of the SVN and BVN with a uniportal—either transforaminal or interlaminar—endoscopic approach [269]. In 14 patients who also had a disc protrusion, nucleoplasty was performed as well. A statistically and clinically significant reduction in pain was observed after 3 months and at the end of a mean follow‐up time of 14.9 months (6–31 months), with NRS reductions of 5.4 and 5.7, respectively. At these follow‐up points, ODI scores also reduced from 68.3 at baseline to, respectively, 22.3 and 21.4 points.

##### 5.4.4.3 Complications

RFA of the BVN appears to have a good safety profile, as adverse events are rare and mostly self‐limiting. In 2022, Conger et al. published a single‐arm systematic review and meta‐analysis that included a total of 414 participants with CVLBP who underwent RFA of the BVN [272]. The most commonly reported adverse events were lumbar/buttock pain (1.0%), leg pain or leg paresthesia due to radiculopathy (3.4%) and dysesthesia (0.7%), which were usually temporary. Rare complications included retroperitoneal hemorrhage (*n* = 1), psoas hematoma (*n* = 1) and iliopsoas hematoma (*n* = 1, treated with embolization).

In the current literature, little is known about the complication rate regarding RFA of the SVN. In the study by Kim et al. only 1 complication in 30 patients was reported—a foot drop—which was self‐limiting. Whether this was caused by the RFA of the BVN or the SVN was unclear [269].

##### 5.4.4.4 Considerations

RFA of the BVN appears to be an effective treatment for patients with CVLBP. However, given the paucity of evidence for efficacy, RFA of the SVN or the BVN should not be offered to patients with CDLBP. Conducting non‐industry‐funded RCTs focusing solely on these therapies (without additional therapy) and in patients with CDLBP is a necessary first step. Another avenue of interest is determining whether RFA of the SVN and/or the BVN is more effective in specific subgroups of patients with CDLBP, for example those with severe Modic changes or fissures posteriorly or close to the endplates.

#### 5.4.5 Spinal Cord Stimulation

In SCS, implanted leads in the epidural space conduct an electrical field transmitting and stimulating the dorsal column of the spinal cord. It is hypothesized that activation of mechanoreceptors in the dorsal column inhibits the transmission of nociceptive pain signals in second order neurons in the dorsal horn (also known as the “gate‐control mechanism”). Furthermore, the increased release of serotonin, norepinephrine, and acetylcholine in the dorsal horn, the activation of spinal gamma‐aminobutyric acid (GABAergic) pathways, the depression of glial cells, and the modification of supraspinal pathways, have been proposed as additional mechanisms [274, 275]. Over the years, different modes of SCS have been developed. Two of the new variants of SCS include “subthreshold” or “high‐frequency”, defined as tonic stimulation at 1–10 kHz, and “burst”, defined as intermittent bursts of stimulation [276, 277, 278].

##### 5.4.5.1 Evidence

SCS has been proven to be a safe and effective treatment for certain chronic pain indications, including non‐operated low back pain [279, 280, 281, 282, 283]. For specific CDLBP, only a few cohort studies have been conducted. In 2012, Vallejo et al. prospectively followed ten patients with CDLBP (confirmed by provocative CT discography and refractory to other treatments) treated with a 10‐day SCS trial [284]. Nine patients had a positive trial (at least 50% pain reduction on the NRS) and received a permanent SCS. After 12 months with one patient lost to follow‐up, a 62% pain reduction was observed (NRS pain score declined from 7.7 to 2.9). The disability scores, however, were not significantly improved. The mode of SCS was not specified in the published article. In 2023, Mons et al. prospectively followed 17 patients with severe CDLBP (proven with provocative discography and refractory to conservative and minimally invasive treatments) treated with a 10‐to‐14‐day SCS trial. Fifteen patients had a positive trial (at least 50% reduction on the NRS) and received a permanent SCS [285]. After 12 months, disability and pain remained significantly reduced (ODI score declined from 41.2 to 25.8 and NRS pain score declined from 7.2 to 4.3). Patient satisfaction and quality of life did not improve significantly.

##### 5.4.5.2 Complications

General complications of SCS for CDLBP are the same as SCS complications for different indications. In 2023, Rauck et al. published the results of a global registry that included 1881 patients with an implanted SCS device for multiple indications, prospectively followed for 3 years [286]. A total of 1289 received a permanent SCS implant after a positive trial. During follow‐up, 7.6% of the permanent devices were explanted, of which 2.5% indicated inadequate pain relief as the cause for removal. 6.2% underwent revision due to technical problems such as device migration or high impedance. Among the 1881 consenting patients, 38 (2%) patients had an infection (one of which was bacterial meningitis), four patients (0.2%) had an epidural hematoma, and 25 patients (1.3%) complained of pain. Rarer adverse events (< 0.3%) included migraine, (pulmonary and intracranial) hypotension, muscular weakness, and bradycardia.

##### 5.4.5.3 Consideration

Only two small prospective cohort trials performing SCS in patients with CDLBP have been published. Furthermore, pre‐clinical and clinical studies both suggest a higher likelihood of success for neuropathic than non‐neuropathic pain states [287, 288]. Therefore, SCS should not be considered as standard therapy for CDLBP. Nonetheless, given the promising results, the safety, and reversibility of this therapy, we encourage future large‐scale RCTs and cost‐effectiveness analyses.

#### 5.4.6 Dorsal Root Ganglion Stimulation

Dorsal root ganglion stimulation (DRG‐S) has been proposed as an alternative to SCS. It uses a similar stimulation strategy, but the leads are placed near the affected DRGs in the intervertebral foramen. DRG‐S is a safe and effective treatment for different neuropathic pain syndromes, including failed back surgery syndrome (FBSS) and FBSS with a discogenic component [289].

The mechanism of action is poorly understood but several mechanisms may be involved. These include the modulation of nociceptive signals at the bifurcation of sensory neuron axons, potentially covering multiple dermatomes by the convergence of peripheral nerves and orthodromic propagation of signals into the dorsal horn; Aδ‐fiber stimulation that causes down regulation of pain neurotransmitters and increased gate‐control in the dorsal horn [10, 290].

It has been hypothesized that the L2 level serves as an important relay station in the conduction of painful stimuli from the lumbar spinal cord and therefore is a therapeutic target. Pain signaling from the lower lumbar discs follows the sympathetic trunk and converges via the caudal‐most white ramus communicans nerve to the dorsal horn [291, 292, 293, 294].

Due to the proximity of DRG to the lead, lower frequencies and amplitudes, and thus smaller electric fields, are sufficient for analgesia than with SCS. It is possible that frequencies as low as 1 Hz are effective, but the optimal programs are still unknown [10].

##### 5.4.6.1 Evidence

For several indications, DRG‐S has been proven to be an effective therapy [293, 295, 296, 297, 298, 299, 300]. In 2020, Kallewaard et al. [292] conducted an observational study in which twenty patients with CDLBP confirmed by provocative discography and a negative medial branch block without a history of back surgery underwent a 14‐day trial of bilateral DRG‐S at L2. One patient had such extensive adhesions it was impossible to place the lead. Fifteen patients had at least 50% pain reduction and received a permanent stimulator. Significant pain reduction was reported at follow‐up at 2 weeks (78.7%), 3 months (64.8%), 6 months (63.4%), and 12 months (68.3%) and commensurate improvements in disability (ODI scores decreased from 42.09 to 20.1) and quality of life (EQ‐5D index increased from 0.61 to 0.84) at 12 months. In 2024, Mons et al. compared the results of their 2023 SCS study with those of the 2023 study by Kallewaard and colleagues. They concluded that DRG‐S of L2 resulted in significantly greater pain relief and disability reduction at 12 months than burst SCS. Obviously, this conclusion is limited by the small study populations and indirect comparison (i.e., different selection criteria).

##### 5.4.6.2 Complications

Rates of device‐related adverse events with DRG‐S appear to be similar to SCS, with the most frequently reported being lead fractures (5.9%), lead migration (5.9%), infections (1.1%–5.1%) and dural puncture (4.3%). Rarer complications include pain, CSF leaks and motor weakness [289, 301]. In the study by Huygen et al. 6.3% of the DRG‐S devices were explanted, mostly due to infection (3.2%) or lack of pain relief (2.0%) [289].

##### 5.4.6.3 Considerations

Only two small prospective cohort studies performing DRG‐S in patients with CDLBP have been published. Therefore, it should not (yet) be offered as standard therapy for CDLBP. However, given the promising results, we encourage future, large‐scale RCTs and cost‐effectiveness analyses. Additionally, the comparison with SCS should be replicated in a large RCT that utilizes state‐of‐the‐art technology.

## 6 Summary of the Evidence

The published evidence of interventional pain management of CDLBP is summarized in Table 2.

**TABLE 2:** Evidence of interventional pain management of CDLBP.

Technique	Level of evidence	Best available evidence + effect (size)^a^	Recommendation
Biacuplasty	Moderate	3 RCTs [237, 243, 245]: reduced pain (1.6, 1.8 and 2.6) and ODI scores (8 and 12) at 6 months. The effects in the intervention cohorts sustained at 12 months (not sham‐controlled) [242, 244]	Can be performed for refractory cases in selected centers
Spinal cord stimulation	Moderate	No RCTs Prospective cohort [284]: positive outcome in 9/10 patients, reduced pain scores (4.8), but no improvement in disability at 12 months. Prospective cohort [285]: positive outcome in 15/17 patients, reduced pain scores (2.9) and ODI (15.4), but no improved patient satisfaction or quality of life at 12 months	Could be considered only in severe cases when all other treatments have failed
IDET	Low	RCT [231]: sham‐controlled trial negative at 6 months RCT [230]: sham‐controlled trial reduced pain (1.3) and ODI scores (7) after 6 months with a number needed to treat of 5 2 single‐arm MA [232, 233]: reduced pain scores (2.9–3.4) and ODI (5.2–7.0) at 6–34 months	Can be considered for refractory cases with debilitating pain, but ideally in a study setting
DRG stimulation (L2)	Very low	No RCTs Prospective cohort [292]: 68.3% of patients had significant pain reduction, reduced ODI (21.99) and increased EQ‐5D index (0.23) at 12 months	Should not be considered as standard treatment but may be considered as rescue therapy for refractory cases or as part of a clinical study
Intradiscal corticosteroid injection	Low	2 RCTs [120, 121]: negative at 2 weeks and 12 months RCT [122]: reduced pain scores and ODI at 3 and 6 months RCT [123]: reduced pain scores (1.5 points), but not functional outcomes at 1 month, negative at 12 months RCT [124] (control group: lidocaine): reduced pain scores (2.8 points) at 1 month, but not at 3 or 6 months	Should not be used outside of a study setting
Intradiscal methylene blue injection	Low	RCT [130]: negative at 6 months RCT [131]: reduced pain and ODI scores (approx. 4.5 and 33, respectively) at 6, 12 and 24 months 1 single‐arm MA [135]: reduced pain and ODI scores (3.6 and 25, respectively, at 3 months, 3.0 and 23 at 6 months, 3.2 and 30 at 12 months)	Should not be used outside of a study setting
PIRFT	Low	RCT [250]: negative at 8 weeks	Should not be used outside of a study setting
Mesenchymal stem cells injection	Very low	RCT [192]: negative at 3, 6 and 12 months RCT [191]: improvement in VAS and ODI until 36 months Single‐arm MA [186]: reduced pain scores (3.7) and ODI (26) at 12 months	Should not be used outside of a study setting
Platelet rich plasma injection	Very low	RCT [198]: negative at 2 months RCT [152]: reduced “best pain scores” (0.7), but no improved functional outcomes at 2 months 4 single‐arm MA [184, 185, 186, 188]: reduced pain scores (approx. 4)	Should not be used outside of a study setting
Cytokine antagonists	Very low	2 RCTs [216, 217]: negative at 1–2 months	Should not be used outside of a study setting
Disctrode	Very low	RCT [248]: negative at 6 and 12 months	Should not be used outside of a study setting
Pulsed RF of the disc	Very low	No RCTs Prospective cohort [257]: reduced pain scores (2) and ODI (10.6) at 12 months Prospective cohort [255]: reduced pain scores (4.7) at 6 months Prospective cohort [256]: 38% of patients had significant pain reduction at 3 months	Should not be used outside of a study setting
Nucleoplasty	Very low	No RCTs 4 prospective cohorts [261, 262, 263, 264]: 53%–74% of patients had significant pain reduction, reduced pain scores (3) and ODI (21), increased SF‐36 (12) at 12 months	Primary indication is radicular pain from a contained herniation. Should not be used outside of a study setting
RFA of the ramus communicans	Very low	RCT [12]: negative at 1 and 3 months RCT [11] (control group: lidocaine): reduced pain scores (2.6) and improved SF‐36 scores (12.8) at 4 months	Should not be used outside of a study setting
RFA of the SVN/BVN	Very Low	No RCTs Prospective cohort [269]: reduced pain scores (5.7) and ODI (52.7) at 6–31 months	Should not be used outside of a study setting

Abbreviations: BVN, basivertebral nerve; CDLBP, chronic discogenic low back pain; COMI, Core Outcome Measures Index (0–10); DRG, dorsal root ganglion; IDET, intradiscal electrothermal therapy; MA, meta‐analysis; ODI, Oswestry Disability Index (0–100); PIRFT, percutaneous intradiscal radiofrequency thermocoagulation; RCT, randomized controlled trial; RFA, radiofrequency ablation; RMDQ, Roland Morris Disability Questionnaire (0–24); SCS, spinal cord stimulation; SF‐36, 36‐Item Short‐Form Health Survey; SVN, sinuvertebral nerve; VAS, visual analog scale.

^a^ Effect sizes are presented as mean (change) differences between groups in the case of RCTs or MA or as mean differences from baseline in the case of prospective trials or single‐arm meta‐analyses.

**FIGURE 8:**
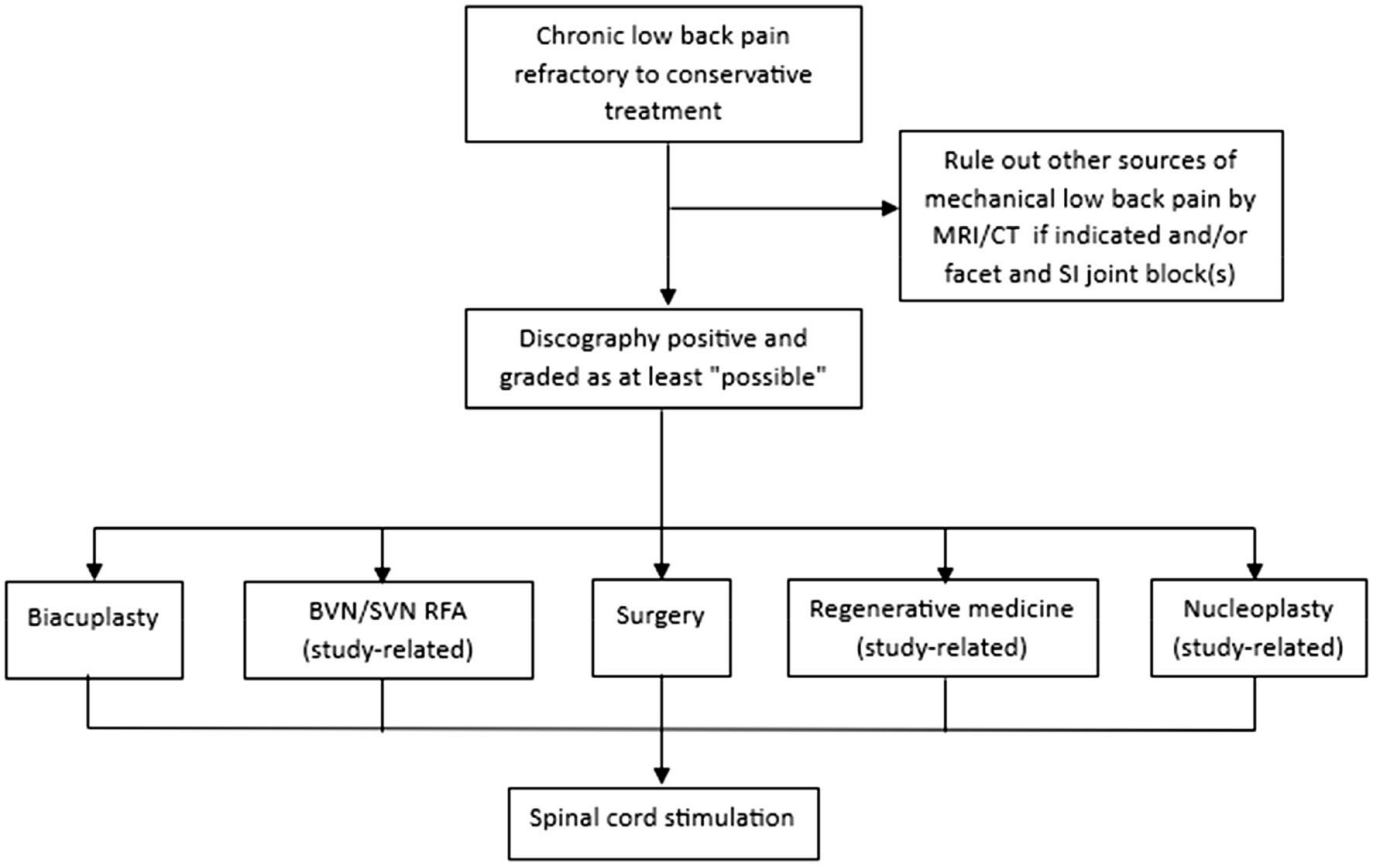
Algorithmic approach for the treatment of chronic discogenic low back pain. BVN, basivertebral nerve; CT, computed tomography; MRI, magnetic resonance imaging; RFA, radiofrequency ablation; SI, sacroiliac; SVN, sinuvertebral nerve.

A practice algorithm for the management of CDLBP is illustrated in Figure 8.

## Author Contributions

Wouter K. M. van Os performed the literature search, performed the literature selection and wrote the manuscript together with Ricardo Alvarez‐Jimenez. Steven P. Cohen, Milan P. Stojanovic, and Jan Van Zundert edited and revised the manuscript, and provided additional references and comments. Ricardo Ruiz‐Lopez provided additional comments and references to the manuscript. Jan Willem Kallewaard edited and revised the manuscript, he provided additional references and comments, and he has full responsibility for the end product.

## Ethics Statement

The authors have nothing to report.

## Consent

The authors have nothing to report.

## Conflicts of Interest

Dr. Jan Van Zundert and dr. Ricardo Ruiz‐Lopez are Editorial Board member of Pain Practice and a co‐author of this article. To minimize bias, they were excluded from all editorial decision‐making related to the acceptance of this article for publication.

## Data Availability

The authors have nothing to report.

## References

[1] J. W. Kallewaard , M. A. Terheggen , G. J. Groen , et al., “15. Discogenic Low Back Pain,” Pain Practice 10 (2010): 560–579. 20825564 10.1111/j.1533-2500.2010.00408.x

[2] GBD 2021 Low Back Pain Collaborators , “Global, Regional, and National Burden of Low Back Pain, 1990–2020, Its Attributable Risk Factors, and Projections to 2050: A Systematic Analysis of the Global Burden of Disease Study 2021,” Lancet Rheumatology 5 (2023): e316–e329. 37273833 10.1016/S2665-9913(23)00098-X PMC10234592

[3] M. J. DePalma , J. M. Ketchum , and T. Saullo , “What Is the Source of Chronic Low Back Pain and Does Age Play a Role?,” Pain Medicine 12 (2011): 224–233. 21266006 10.1111/j.1526-4637.2010.01045.x

[4] N. Inoue , A. A. E. Orías , and K. Segami , “Biomechanics of the Lumbar Facet Joint,” Spine Surgery and Related Research 4 (2020): 1–7. 32039290 10.22603/ssrr.2019-0017 PMC7002062

[5] M. A. Edgar , “The Nerve Supply of the Lumbar Intervertebral Disc,” Journal of Bone and Joint Surgery (British Volume) 89 (2007): 1135–1139. 17905946 10.1302/0301-620X.89B9.18939

[6] G. J. Groen , B. Baljet , and J. Drukker , “The Innervation of the Spinal Dura Mater: Anatomy and Clinical Implications,” Acta Neurochirurgica 92 (1988): 39–46. 3407473 10.1007/BF01401971

[7] G. J. Groen , B. Baljet , and J. Drukker , “Nerves and Nerve Plexuses of the Human Vertebral Column,” American Journal of Anatomy 188 (1990): 282–296. 2371968 10.1002/aja.1001880307

[8] H. Brisby , “Pathology and Possible Mechanisms of Nervous System Response to Disc Degeneration,” Journal of Bone and Joint Surgery (American Volume) 88 Suppl 2 (2006): 68–71. 16595447 10.2106/JBJS.E.01282

[9] A. M. R. Groh , D. E. Fournier , M. C. Battié , and C. A. Séguin , “Innervation of the Human Intervertebral Disc: A Scoping Review,” Pain Medicine 22 (2021): 1281–1304. 33595648 10.1093/pm/pnab070 PMC8185559

[10] K. B. Chapman , D. Sayed , T. Lamer , et al., “Best Practices for Dorsal Root Ganglion Stimulation for Chronic Pain: Guidelines From the American Society of Pain and Neuroscience,” Journal of Pain Research 16 (2023): 839–879. 36942306 10.2147/JPR.S364370 PMC10024474

[11] W. S. Oh and J. C. Shim , “A Randomized Controlled Trial of Radiofrequency Denervation of the Ramus Communicans Nerve for Chronic Discogenic Low Back Pain,” Clinical Journal of Pain 20 (2004): 55–60. 14668658 10.1097/00002508-200401000-00011

[12] C. W. van Tilburg , D. L. Stronks , J. G. Groeneweg , and F. J. Huygen , “Randomized Sham‐Controlled, Double‐Blind, Multicenter Clinical Trial on the Effect of Percutaneous Radiofrequency at the Ramus Communicans for Lumbar Disc Pain,” European Journal of Pain 21 (2017): 520–529. 27734550 10.1002/ejp.945 PMC5324589

[13] M. A. Adams and P. J. Roughley , “What Is Intervertebral Disc Degeneration, and What Causes It?,” Spine 31 (2006): 2151–2161. 16915105 10.1097/01.brs.0000231761.73859.2c

[14] C. Shi , S. Qiu , S. M. Riester , et al., “Animal Models for Studying the Etiology and Treatment of Low Back Pain,” Journal of Orthopaedic Research 36 (2018): 1305–1312. 28921656 10.1002/jor.23741 PMC6287742

[15] S. P. Cohen , T. M. Larkin , S. A. Barna , W. E. Palmer , A. C. Hecht , and M. P. Stojanovic , “Lumbar Discography: A Comprehensive Review of Outcome Studies, Diagnostic Accuracy, and Principles,” Regional Anesthesia and Pain Medicine 30 (2005): 163–183. 15765459 10.1016/j.rapm.2004.10.006

[16] W. E. Johnson , B. Caterson , S. M. Eisenstein , D. L. Hynds , D. M. Snow , and S. Roberts , “Human Intervertebral Disc Aggrecan Inhibits Nerve Growth In Vitro,” Arthritis & Rheumatism 46 (2002): 2658–2664. 12384924 10.1002/art.10585

[17] J. M. Lee , J. Y. Song , M. Baek , et al., “Interleukin‐1β Induces Angiogenesis and Innervation in Human Intervertebral Disc Degeneration,” Journal of Orthopaedic Research 29 (2011): 265–269. 20690185 10.1002/jor.21210

[18] A. J. Freemont , T. E. Peacock , P. Goupille , J. A. Hoyland , J. O'Brien , and M. I. Jayson , “Nerve Ingrowth Into Diseased Intervertebral Disc in Chronic Back Pain,” Lancet 350 (1997): 178–181. 9250186 10.1016/s0140-6736(97)02135-1

[19] S. S. Sivan , E. Wachtel , and P. Roughley , “Structure, Function, Aging and Turnover of Aggrecan in the Intervertebral Disc,” Biochimica et Biophysica Acta 1840 (2014): 3181–3189. 25065289 10.1016/j.bbagen.2014.07.013

[20] J. P. Urban and S. Roberts , “Degeneration of the Intervertebral Disc,” Arthritis Research & Therapy 5 (2003): 120–130. 12723977 10.1186/ar629 PMC165040

[21] C. Weiler , M. Lopez‐Ramos , H. M. Mayer , et al., “Histological Analysis of Surgical Lumbar Intervertebral Disc Tissue Provides Evidence for an Association Between Disc Degeneration and Increased Body Mass Index,” BMC Research Notes 4 (2011): 497. 22087871 10.1186/1756-0500-4-497 PMC3226673

[22] M. J. Depalma , J. M. Ketchum , B. S. Trussell , T. R. Saullo , and C. W. Slipman , “Does the Location of Low Back Pain Predict Its Source?,” PM & R: The Journal of Injury, Function, and Rehabilitation 3 (2011): 33–39. 10.1016/j.pmrj.2010.09.006 21257131

[23] S. Young , C. Aprill , and M. Laslett , “Correlation of Clinical Examination Characteristics With Three Sources of Chronic Low Back Pain,” Spine Journal 3 (2003): 460–465. 10.1016/s1529-9430(03)00151-7 14609690

[24] M. A. Adams , B. J. Freeman , H. P. Morrison , I. W. Nelson , and P. Dolan , “Mechanical Initiation of Intervertebral Disc Degeneration,” Spine 25 (2000): 1625–1636. 10870137 10.1097/00007632-200007010-00005

[25] M. A. Adams , M. Stefanakis , and P. Dolan , “Healing of a Painful Intervertebral Disc Should Not Be Confused With Reversing Disc Degeneration: Implications for Physical Therapies for Discogenic Back Pain,” Clinical Biomechanics (Bristol, Avon) 25 (2010): 961–971. 20739107 10.1016/j.clinbiomech.2010.07.016

[26] Y. G. Zhang , T. M. Guo , X. Guo , and S. X. Wu , “Clinical Diagnosis for Discogenic Low Back Pain,” International Journal of Biological Sciences 5 (2009): 647–658. 19847321 10.7150/ijbs.5.647 PMC2764347

[27] C. W. O'Neill , M. E. Kurgansky , R. Derby , and D. P. Ryan , “Disc Stimulation and Patterns of Referred Pain,” Spine 27 (2002): 2776–2781. 12486346 10.1097/00007632-200212150-00007

[28] D. D. Ohnmeiss , H. Vanharanta , and J. Ekholm , “Relationship of Pain Drawings to Invasive Tests Assessing Intervertebral Disc Pathology,” European Spine Journal 8 (1999): 126–131. 10333151 10.1007/s005860050141 PMC3611154

[29] D. D. Ohnmeiss , H. Vanharanta , and J. Ekholm , “Relation Between Pain Location and Disc Pathology: A Study of Pain Drawings and CT/Discography,” Clinical Journal of Pain 15 (1999): 210–217. 10524474 10.1097/00002508-199909000-00008

[30] M. Laslett , C. N. Aprill , B. McDonald , and B. Oberg , “Clinical Predictors of Lumbar Provocation Discography: A Study of Clinical Predictors of Lumbar Provocation Discography,” European Spine Journal 15 (2006): 1473–1484. 16474943 10.1007/s00586-006-0062-7

[31] C. S. Han , M. J. Hancock , S. Sharma , et al., “Low Back Pain of Disc, Sacroiliac Joint, or Facet Joint Origin: A Diagnostic Accuracy Systematic Review,” EClinicalMedicine 59 (2023): 101960. 37096189 10.1016/j.eclinm.2023.101960 PMC10121397

[32] C. S. Han , M. J. Hancock , A. Downie , et al., “Red Flags to Screen for Vertebral Fracture in People Presenting With Low Back Pain,” Cochrane Database of Systematic Reviews 8 (2023): Cd014461. 37615643 10.1002/14651858.CD014461.pub2 PMC10448864

[33] M. DePalma , J. Ketchum , T. Saullo , and J. Schofferman , “Structural Etiology of Chronic Low Back Pain due to Motor Vehicle Collision,” Pain Medicine 12 (2011): 1622–1627. 21958329 10.1111/j.1526-4637.2011.01246.x

[34] E. Remotti , C. Nduaguba , P. A. Woolley , et al., “Review: Discogenic Back Pain: Update on Treatment,” Orthopedic Reviews 15 (2023): 84649. 37641793 10.52965/001c.84649 PMC10460631

[35] M. Yrjämä and H. Vanharanta , “Bony Vibration Stimulation: A New, Non‐Invasive Method for Examining Intradiscal Pain,” European Spine Journal 3 (1994): 233–235. 7866843 10.1007/BF02221600

[36] H. Vanharanta , D. D. Ohnmeiss , and C. N. Aprill , “Vibration Pain Provocation Can Improve the Specificity of MRI in the Diagnosis of Symptomatic Lumbar Disc Rupture,” Clinical Journal of Pain 14 (1998): 239–247. 9758074 10.1097/00002508-199809000-00011

[37] M. Yrjämä , O. Tervonen , M. Kurunlahti , and H. Vanharanta , “Bony Vibration Stimulation Test Combined With Magnetic Resonance Imaging. Can Discography Be Replaced?,” Spine 22 (1997): 808–813. 9106323 10.1097/00007632-199704010-00020

[38] M. Yrjämä , O. Tervonen , and H. Vanharanta , “Ultrasonic Imaging of Lumbar Discs Combined With Vibration Pain Provocation Compared With Discography in the Diagnosis of Internal Anular Fissures of the Lumbar Spine,” Spine 21 (1996): 571–575. 8852311 10.1097/00007632-199603010-00007

[39] R. McKenzie and S. May , “Discogenic Pain—Clinical Features,” in The Lumbar Spine: Mechanical Diagnosis and Therapy, vol. 1, ed. S. May (Spinal Publications, 2003), 89–90.

[40] T. Saueressig , P. J. Owen , F. Diemer , J. Zebisch , and D. L. Belavy , “Diagnostic Accuracy of Clusters of Pain Provocation Tests for Detecting Sacroiliac Joint Pain: Systematic Review With Meta‐Analysis,” Journal of Orthopaedic and Sports Physical Therapy 51 (2021): 422–431. 34210160 10.2519/jospt.2021.10469

[41] L. Peene , S. P. Cohen , J. W. Kallewaard , et al., “1. Lumbosacral Radicular Pain,” Pain Practice 24 (2024): 525–552. 37985718 10.1111/papr.13317

[42] T. D. Ross , S. Evans , D. P. Ahern , J. McDonnell , and J. S. Butler , “Discography or SPECT/CT: What Is the Best Diagnostic Tool for the Surgical Assessment of Degenerative Disk Disease?,” Clinical Spine Surgery 34 (2021): 355–358. 32649338 10.1097/BSD.0000000000001042

[43] A. T. Trout , S. E. Sharp , C. G. Anton , M. J. Gelfand , and C. T. Mehlman , “Spondylolysis and Beyond: Value of SPECT/CT in Evaluation of Low Back Pain in Children and Young Adults,” Radiographics 35 (2015): 819–834. 25969937 10.1148/rg.2015140092

[44] R. Ruiz‐Lopez and M. Squarcia , “Usefulness of SPECT‐TC in the Evaluation of Chronic Spinal Pain: A New Diagnostic Tool,” 2024.

[45] C. Fang , W. Zhang , L. Chen , and H. Li , “The Correlation Between the High‐Intensity Zone on a T2‐Weighted MRI and Positive Outcomes of Discography: A Meta‐Analysis,” Journal of Orthopaedic Surgery and Research 12 (2017): 26. 28178999 10.1186/s13018-017-0523-1 PMC5299742

[46] C. O'Neill , M. Kurgansky , J. Kaiser , and W. Lau , “Accuracy of MRI for Diagnosis of Discogenic Pain,” Pain Physician 11 (2008): 311–326. 18523502

[47] W. Brinjikji , F. E. Diehn , J. G. Jarvik , et al., “MRI Findings of Disc Degeneration Are More Prevalent in Adults With Low Back Pain Than in Asymptomatic Controls: A Systematic Review and Meta‐Analysis,” AJNR. American Journal of Neuroradiology 36 (2015): 2394–2399. 26359154 10.3174/ajnr.A4498 PMC7964277

[48] H. Manabe , T. Sakai , Y. Omichi , et al., “Role of Growth Plate (Apophyseal Ring Fracture) in Causing Modic Type Changes in Pediatric Low Back Pain Patients,” European Spine Journal 30 (2021): 2565–2569. 34037865 10.1007/s00586-021-06885-2

[49] N. Bogduk , Practice Guidelines for Spinal Diagnostic and Treatment Procedures (International Spine Intervention Society, 2013).

[50] L. Manchikanti , S. E. Glaser , L. Wolfer , R. Derby , and S. P. Cohen , “Systematic Review of Lumbar Discography as a Diagnostic Test for Chronic Low Back Pain,” Pain Physician 12 (2009): 541–559. 19461822

[51] L. Manchikanti , A. Soin , R. M. Benyamin , et al., “An Update of the Systematic Appraisal of the Accuracy and Utility of Discography in Chronic Spinal Pain,” Pain Physician 21 (2018): 91–110. 29565943

[52] T. P. Maus and C. N. Aprill , “Lumbar Diskogenic Pain, Provocation Diskography, and Imaging Correlates,” Radiologic Clinics of North America 50 (2012): 681–704. 22643391 10.1016/j.rcl.2012.04.013

[53] G. B. Moneta , T. Videman , K. Kaivanto , et al., “Reported Pain During Lumbar Discography as a Function of Anular Ruptures and Disc Degeneration. A Re‐Analysis of 833 Discograms,” Spine 19 (1994): 1968–1974. 7997931 10.1097/00007632-199409000-00018

[54] B. G. Peng , X. D. Pang , D. M. Li , et al., “Natural History and Prognosis of Discogenic Low Back Pain,” Zhonghua Yi Xue Za Zhi 89 (2009): 2171–2174. 20058592

[55] E. J. Carragee , T. F. Alamin , and J. M. Carragee , “Low‐Pressure Positive Discography in Subjects Asymptomatic of Significant Low Back Pain Illness,” Spine 31 (2006): 505–509. 16508542 10.1097/01.brs.0000201242.85984.76

[56] E. J. Carragee , T. F. Alamin , J. Miller , and M. Grafe , “Provocative Discography in Volunteer Subjects With Mild Persistent Low Back Pain,” Spine Journal 2 (2002): 25–34. 10.1016/s1529-9430(01)00152-8 14588285

[57] E. J. Carragee , T. Lincoln , V. S. Parmar , and T. Alamin , “A Gold Standard Evaluation of the ‘Discogenic Pain’ Diagnosis as Determined by Provocative Discography,” Spine 31 (2006): 2115–2123. 16915099 10.1097/01.brs.0000231436.30262.dd

[58] E. J. Carragee , C. M. Tanner , B. Yang , J. L. Brito , and T. Truong , “False‐Positive Findings on Lumbar Discography. Reliability of Subjective Concordance Assessment During Provocative Disc Injection,” Spine 24 (1999): 2542–2547. 10626318 10.1097/00007632-199912010-00017

[59] L. R. Wolfer , R. Derby , J. E. Lee , and S. H. Lee , “Systematic Review of Lumbar Provocation Discography in Asymptomatic Subjects With a Meta‐Analysis of False‐Positive Rates,” Pain Physician 11 (2008): 513–538. 18690280

[60] J. W. Kallewaard , J. W. Geurts , M. Terheggen , et al., “No Transfer of Pressure to Adjacent Discs During Human Low‐Pressure Controlled Discography: A Prospective Clinical Study,” Pain Medicine 19 (2018): 29–39. 28379575 10.1093/pm/pnx039

[61] H. Hebelka , A. Nilsson , and T. Hansson , “Pressure Increase in Adjacent Discs During Clinical Discography Questions the Methods Validity,” Spine 39 (2014): 893–899. 24365908 10.1097/BRS.0000000000000166

[62] H. Hebelka , A. Gaulitz , A. Nilsson , S. Holm , and T. Hansson , “The Transfer of Disc Pressure to Adjacent Discs in Discography: A Specificity Problem?,” Spine 35 (2010): E1025–E1029. 20802394 10.1097/BRS.0b013e3181dc9e0f

[63] R. D. Guyer , R. Collier , W. J. Stith , et al., “Discitis After Discography,” Spine 13 (1988): 1352–1354. 3212569 10.1097/00007632-198812000-00004

[64] R. D. Guyer and D. D. Ohnmeiss , “Lumbar Discography,” Spine Journal 3 (2003): 11s–27s. 10.1016/s1529-9430(02)00563-6 14589214

[65] P. C. Willems , W. Jacobs , E. S. Duinkerke , and M. De Kleuver , “Lumbar Discography: Should We Use Prophylactic Antibiotics? A Study of 435 Consecutive Discograms and a Systematic Review of the Literature,” Journal of Spinal Disorders & Techniques 17 (2004): 243–247. 15167342 10.1097/00024720-200406000-00013

[66] S. K. Sharma , J. O. Jones , P. P. Zeballos , S. A. Irwin , and T. W. Martin , “The Prevention of Discitis During Discography,” Spine Journal 9 (2009): 936–943. 10.1016/j.spinee.2009.06.001 19643677

[67] C. Cunha , S. Lamas , R. M. Gonçalves , and M. A. Barbosa , “Joint Analysis of IVD Herniation and Degeneration by Rat Caudal Needle Puncture Model,” Journal of Orthopaedic Research 35 (2017): 258–268. 26610284 10.1002/jor.23114

[68] G. Keorochana , J. S. Johnson , C. E. Taghavi , et al., “The Effect of Needle Size Inducing Degeneration in the Rat Caudal Disc: Evaluation Using Radiograph, Magnetic Resonance Imaging, Histology, and Immunohistochemistry,” Spine Journal 10 (2010): 1014–1023. 10.1016/j.spinee.2010.08.013 20970740

[69] K. Masuda , Y. Aota , C. Muehleman , et al., “A Novel Rabbit Model of Mild, Reproducible Disc Degeneration by an Anulus Needle Puncture: Correlation Between the Degree of Disc Injury and Radiological and Histological Appearances of Disc Degeneration,” Spine 30 (2005): 5–14. 15626974 10.1097/01.brs.0000148152.04401.20

[70] S. Sobajima , J. F. Kompel , J. S. Kim , et al., “A Slowly Progressive and Reproducible Animal Model of Intervertebral Disc Degeneration Characterized by MRI, X‐Ray, and Histology,” Spine 30 (2005): 15–24. 15626975 10.1097/01.brs.0000148048.15348.9b

[71] K. Ura , H. Sudo , K. Iwasaki , T. Tsujimoto , D. Ukeba , and N. Iwasaki , “Effects of Intradiscal Injection of Local Anesthetics on Intervertebral Disc Degeneration in Rabbit Degenerated Intervertebral Disc,” Journal of Orthopaedic Research 37 (2019): 1963–1971. 31106893 10.1002/jor.24347

[72] J. Y. Wang , J. C. Mansfield , S. Brasselet , C. Vergari , J. R. Meakin , and C. P. Winlove , “Micro‐Mechanical Damage of Needle Puncture on Bovine Annulus Fibrosus Fibrils Studied Using Polarization‐Resolved Second Harmonic Generation (P‐SHG) Microscopy,” Journal of the Mechanical Behavior of Biomedical Materials 118 (2021): 104458. 33761373 10.1016/j.jmbbm.2021.104458

[73] E. J. Carragee , A. S. Don , E. L. Hurwitz , J. M. Cuellar , J. A. Carrino , and R. Herzog , “2009 ISSLS Prize Winner: Does Discography Cause Accelerated Progression of Degeneration Changes in the Lumbar Disc: A Ten‐Year Matched Cohort Study,” Spine 34 (2009): 2338–2345. 19755936 10.1097/BRS.0b013e3181ab5432

[74] J.‐W. Hur , “Long‐Term Follow‐Up of Disc Degeneration After Discography,” Spine Journal 17 (2017): S121.

[75] M. Pinto , A. A. Mehbod , B. A. Swanberg , J. M. Dawson , and K. Schellhas , “Provocative Discography: Diagnostic Efficacy and Safety in Symptomatic Degenerative Disk Disease,” Clinical Spine Surgery 35 (2022): E571–e575. 35894509 10.1097/BSD.0000000000001329

[76] A. H. Hsieh , D. Hwang , D. A. Ryan , A. K. Freeman , and H. Kim , “Degenerative Anular Changes Induced by Puncture Are Associated With Insufficiency of Disc Biomechanical Function,” Spine 34 (2009): 998–1005. 19404174 10.1097/BRS.0b013e31819c09c4

[77] X. Huang , W. Wang , Q. Meng , et al., “Effect of Needle Diameter, Type and Volume of Contrast Agent on Intervertebral Disc Degeneration in Rats With Discography,” European Spine Journal 28 (2019): 1014–1022. 30864063 10.1007/s00586-019-05927-0

[78] V. M. van Heeswijk , A. Thambyah , P. A. Robertson , and N. D. Broom , “Does an Annular Puncture Influence the Herniation Path?: An In Vitro Mechanical and Structural Investigation,” Spine 43 (2018): 467–476. 28719550 10.1097/BRS.0000000000002336

[79] Z. L. McCormick , V. T. Lehman , C. T. Plastaras , et al., “Low‐Pressure Lumbar Provocation Discography According to Spine Intervention Society/International Association for the Study of Pain Standards Does Not Cause Acceleration of Disc Degeneration in Patients With Symptomatic Low Back Pain: A 7‐Year Matched Cohort Study,” Spine 44 (2019): e1161–e1168. 31261283 10.1097/BRS.0000000000003085

[80] J. M. Cuellar , M. P. Stauff , R. J. Herzog , J. A. Carrino , G. A. Baker , and E. J. Carragee , “Does Provocative Discography Cause Clinically Important Injury to the Lumbar Intervertebral Disc? A 10‐Year Matched Cohort Study,” Spine Journal 16 (2016): 273–280. 10.1016/j.spinee.2015.06.051 26133255

[81] S. M. Iencean , “Lumbar Intervertebral Disc Herniation Following Experimental Intradiscal Pressure Increase,” Acta Neurochirurgica 142 (2000): 669–676. 10949442 10.1007/s007010070111

[82] S. G. Kapoor , J. Huff , and S. P. Cohen , “Systematic Review of the Incidence of Discitis After Cervical Discography,” Spine Journal 10 (2010): 739–745. 10.1016/j.spinee.2009.12.022 20171935

[83] C. O'Neill and M. Kurgansky , “Subgroups of Positive Discs on Discography,” Spine 29 (2004): 2134–2139. 15454704 10.1097/01.brs.0000141169.15283.78

[84] K. S. Seo , R. Derby , E. S. Date , S. H. Lee , B. J. Kim , and C. H. Lee , “In Vitro Measurement of Pressure Differences Using Manometry at Various Injection Speeds During Discography,” Spine Journal 7 (2007): 68–73. 10.1016/j.spinee.2006.05.013 17197335

[85] D. A. Shin , S. H. Kim , I. B. Han , S. C. Rhim , and H. I. Kim , “Factors Influencing Manometric Pressure During Pressure‐Controlled Discography,” Spine 34 (2009): E790–E793. 19829241 10.1097/BRS.0b013e3181ba2a8d

[86] B. L. Sachs , H. Vanharanta , M. A. Spivey , et al., “Dallas Discogram Description. A New Classification of CT/Discography in Low‐Back Disorders,” Spine 12 (1987): 287–294. 2954226 10.1097/00007632-198704000-00018

[87] M. F. Stretanski and L. Vu , “Fluoroscopy Discography Assessment, Protocols, and Interpretation,” in StatPearls. StatPearls Publishing Copyright 2024 (StatPearls Publishing LLC, 2024). 34283485

[88] B. Peng , X. Fu , X. Pang , et al., “Prospective Clinical Study on Natural History of Discogenic Low Back Pain at 4 Years of Follow‐Up,” Pain Physician 15 (2012): 525–532. 23159971

[89] A. M. Jamjoom , R. J. Saeedi , and A. B. Jamjoom , “Placebo Effect of Sham Spine Procedures in Chronic Low Back Pain: A Systematic Review,” Journal of Pain Research 14 (2021): 3057–3065. 34616178 10.2147/JPR.S317697 PMC8488027

[90] J. J. Schimmel , M. de Kleuver , P. P. Horsting , M. Spruit , W. C. Jacobs , and J. van Limbeek , “No Effect of Traction in Patients With Low Back Pain: A Single Centre, Single Blind, Randomized Controlled Trial of Intervertebral Differential Dynamics Therapy,” European Spine Journal 18 (2009): 1843–1850. 19484433 10.1007/s00586-009-1044-3 PMC2899427

[91] A. Macario and J. V. Pergolizzi , “Systematic Literature Review of Spinal Decompression via Motorized Traction for Chronic Discogenic Low Back Pain,” Pain Practice 6 (2006): 171–178. 17147594 10.1111/j.1533-2500.2006.00082.x

[92] J. A. Hayden , J. Ellis , R. Ogilvie , A. Malmivaara , and M. W. van Tulder , “Exercise Therapy for Chronic Low Back Pain,” Cochrane Database of Systematic Reviews 9 (2021): Cd009790. 34580864 10.1002/14651858.CD009790.pub2 PMC8477273

[93] B. T. Saragiotto , C. G. Maher , T. P. Yamato , et al., “Motor Control Exercise for Chronic Non‐Specific Low‐Back Pain,” Cochrane Database of Systematic Reviews 2016 (2016): Cd012004. 26742533 10.1002/14651858.CD012004 PMC8761501

[94] C. Zhang , Y. Li , Y. Zhong , C. Feng , Z. Zhang , and C. Wang , “Effectiveness of Motor Control Exercise on Non‐Specific Chronic Low Back Pain, Disability and Core Muscle Morphological Characteristics: A Meta‐Analysis of Randomized Controlled Trials,” European Journal of Physical and Rehabilitation Medicine 57 (2021): 793–806. 33960180 10.23736/S1973-9087.21.06555-2

[95] A. R. Aladro‐Gonzalvo , G. A. Araya‐Vargas , M. Machado‐Díaz , and W. Salazar‐Rojas , “Pilates‐Based Exercise for Persistent, Non‐Specific Low Back Pain and Associated Functional Disability: A Meta‐Analysis With Meta‐Regression,” Journal of Bodywork and Movement Therapies 17 (2013): 125–136. 23294694 10.1016/j.jbmt.2012.08.003

[96] T. P. Yamato , C. G. Maher , B. T. Saragiotto , et al., “Pilates for Low Back Pain,” Cochrane Database of Systematic Reviews 2015 (2015): Cd010265. 26133923 10.1002/14651858.CD010265.pub2 PMC8078578

[97] A. M. Alhakami , S. Davis , M. Qasheesh , A. Shaphe , and A. Chahal , “Effects of McKenzie and Stabilization Exercises in Reducing Pain Intensity and Functional Disability in Individuals With Nonspecific Chronic Low Back Pain: A Systematic Review,” Journal of Physical Therapy Science 31 (2019): 590–597. 31417227 10.1589/jpts.31.590 PMC6642883

[98] F. I. Namnaqani , A. S. Mashabi , K. M. Yaseen , and M. A. Alshehri , “The Effectiveness of McKenzie Method Compared to Manual Therapy for Treating Chronic Low Back Pain: A Systematic Review,” Journal of Musculoskeletal & Neuronal Interactions 19 (2019): 492–499. 31789300 PMC6944795

[99] L. S. Wieland , N. Skoetz , K. Pilkington , R. Vempati , C. R. D'Adamo , and B. M. Berman , “Yoga Treatment for Chronic Non‐Specific Low Back Pain,” Cochrane Database of Systematic Reviews 1 (2017): Cd010671. 28076926 10.1002/14651858.CD010671.pub2 PMC5294833

[100] F. Zhu , M. Zhang , D. Wang , Q. Hong , C. Zeng , and W. Chen , “Yoga Compared to Non‐Exercise or Physical Therapy Exercise on Pain, Disability, and Quality of Life for Patients With Chronic Low Back Pain: A Systematic Review and Meta‐Analysis of Randomized Controlled Trials,” PLoS One 15 (2020): e0238544. 32870936 10.1371/journal.pone.0238544 PMC7462307

[101] A. Büssing , T. Ostermann , R. Lüdtke , and A. Michalsen , “Effects of Yoga Interventions on Pain and Pain‐Associated Disability: A Meta‐Analysis,” Journal of Pain 13 (2012): 1–9. 22178433 10.1016/j.jpain.2011.10.001

[102] B. Brea‐Gómez , I. Torres‐Sánchez , A. Ortiz‐Rubio , et al., “Virtual Reality in the Treatment of Adults With Chronic Low Back Pain: A Systematic Review and Meta‐Analysis of Randomized Clinical Trials,” International Journal of Environmental Research and Public Health 18 (2021): 11806. 34831562 10.3390/ijerph182211806 PMC8621053

[103] I. D. Coulter , C. Crawford , E. L. Hurwitz , et al., “Manipulation and Mobilization for Treating Chronic Low Back Pain: A Systematic Review and Meta‐Analysis,” Spine Journal 18 (2018): 866–879. 10.1016/j.spinee.2018.01.013 PMC6020029 29371112

[104] S. M. Rubinstein , A. de Zoete , M. van Middelkoop , W. J. J. Assendelft , M. R. de Boer , and M. W. van Tulder , “Benefits and Harms of Spinal Manipulative Therapy for the Treatment of Chronic Low Back Pain: Systematic Review and Meta‐Analysis of Randomised Controlled Trials,” BMJ 364 (2019): l689. 30867144 10.1136/bmj.l689 PMC6396088

[105] G. Petrucci , G. F. Papalia , F. Russo , et al., “Psychological Approaches for the Integrative Care of Chronic low Back Pain: A Systematic Review and Metanalysis,” International Journal of Environmental Research and Public Health 19 (2021): 60. 35010319 10.3390/ijerph19010060 PMC8751135

[106] E. K. Ho , L. Chen , M. Simic , et al., “Psychological Interventions for Chronic, Non‐Specific Low Back Pain: Systematic Review With Network Meta‐Analysis,” BMJ 376 (2022): e067718. 35354560 10.1136/bmj-2021-067718 PMC8965745

[107] S. J. Kamper , A. T. Apeldoorn , A. Chiarotto , et al., “Multidisciplinary Biopsychosocial Rehabilitation for Chronic Low Back Pain: Cochrane Systematic Review and Meta‐Analysis,” BMJ 350 (2015): h444. 25694111 10.1136/bmj.h444 PMC4353283

[108] M. B. Casey , K. M. Smart , R. Segurado , and C. Doody , “Multidisciplinary‐Based Rehabilitation (MBR) Compared With Active Physical Interventions for Pain and Disability in Adults With Chronic Pain: A Systematic Review and Meta‐Analysis,” Clinical Journal of Pain 36 (2020): 874–886. 32773436 10.1097/AJP.0000000000000871

[109] M. van Middelkoop , S. M. Rubinstein , T. Kuijpers , et al., “A Systematic Review on the Effectiveness of Physical and Rehabilitation Interventions for Chronic Non‐Specific Low Back Pain,” European Spine Journal 20 (2011): 19–39. 20640863 10.1007/s00586-010-1518-3 PMC3036018

[110] V. Nicol , C. Verdaguer , C. Daste , et al., “Chronic Low Back Pain: A Narrative Review of Recent International Guidelines for Diagnosis and Conservative Treatment,” Journal of Clinical Medicine 12 (2023): 1685. 36836220 10.3390/jcm12041685 PMC9964474

[111] V. Skljarevski , M. Ossanna , H. Liu‐Seifert , et al., “A Double‐Blind, Randomized Trial of Duloxetine Versus Placebo in the Management of Chronic Low Back Pain,” European Journal of Neurology 16 (2009): 1041–1048. 19469829 10.1111/j.1468-1331.2009.02648.x

[112] V. Skljarevski , S. Zhang , D. Desaiah , et al., “Duloxetine Versus Placebo in Patients With Chronic Low Back Pain: A 12‐Week, Fixed‐Dose, Randomized, Double‐Blind Trial,” Journal of Pain 11 (2010): 1282–1290. 20472510 10.1016/j.jpain.2010.03.002

[113] V. Skljarevski , D. Desaiah , H. Liu‐Seifert , et al., “Efficacy and Safety of Duloxetine in Patients With Chronic Low Back Pain,” Spine 35 (2010): E578–E585. 20461028 10.1097/BRS.0b013e3181d3cef6

[114] J. Nijs , E. Kosek , A. Chiarotto , et al., “Nociceptive, Neuropathic, or Nociplastic Low Back Pain? The Low Back Pain Phenotyping (BACPAP) Consortium's International and Multidisciplinary Consensus Recommendations,” Lancet Rheumatology 6 (2024): e178–e188. 38310923 10.1016/S2665-9913(23)00324-7

[115] H. B. Albert , J. S. Sorensen , B. S. Christensen , and C. Manniche , “Antibiotic Treatment in Patients With Chronic Low Back Pain and Vertebral Bone Edema (Modic Type 1 Changes): A Double‐Blind Randomized Clinical Controlled Trial of Efficacy,” European Spine Journal 22 (2013): 697–707. 23404353 10.1007/s00586-013-2675-y PMC3631045

[116] C. J. Gilligan , S. P. Cohen , V. A. Fischetti , J. A. Hirsch , and L. G. Czaplewski , “Chronic Low Back Pain, Bacterial Infection and Treatment With Antibiotics,” Spine Journal 21 (2021): 903–914. 10.1016/j.spinee.2021.02.013 33610802

[117] L. C. H. Bråten , M. P. Rolfsen , A. Espeland , et al., “Efficacy of Antibiotic Treatment in Patients With Chronic Low Back Pain and Modic Changes (The AIM Study): Double Blind, Randomised, Placebo Controlled, Multicentre Trial,” BMJ 367 (2019): l5654. 31619437 10.1136/bmj.l5654 PMC6812614

[118] T. Farmer , S. C. Morris , R. Quigley , N. H. Amin , M. D. Wongworawat , and H. M. Syed , “Chondrotoxicity of Local Anesthetics: Liposomal Bupivacaine Is Less Chondrotoxic Than Standard Bupivacaine,” Advances in Pharmacology and Pharmaceutical Sciences 2020 (2020): 5794187. 10.1155/2020/5794187 PMC7199567 32399520

[119] J. L. Dragoo , C. M. Danial , H. J. Braun , M. A. Pouliot , and H. J. Kim , “The Chondrotoxicity of Single‐Dose Corticosteroids,” Knee Surgery, Sports Traumatology, Arthroscopy 20 (2012): 1809–1814. 10.1007/s00167-011-1820-6 22186921

[120] J. W. Simmons , J. N. McMillin , S. F. Emery , and S. J. Kimmich , “Intradiscal Steroids. A Prospective Double‐Blind Clinical Trial,” Spine 17 (1992): S172–S175. 1385902

[121] A. Khot , M. Bowditch , J. Powell , and D. Sharp , “The Use of Intradiscal Steroid Therapy for Lumbar Spinal Discogenic Pain: A Randomized Controlled Trial,” Spine 29 (2004): 833–836; discussion 837. 15082979 10.1097/00007632-200404150-00002

[122] P. Cao , L. Jiang , C. Zhuang , et al., “Intradiscal Injection Therapy for Degenerative Chronic Discogenic Low Back Pain With End Plate Modic Changes,” Spine Journal 11 (2011): 100–106. 10.1016/j.spinee.2010.07.001 20850390

[123] C. Nguyen , I. Boutron , G. Baron , et al., “Intradiscal Glucocorticoid Injection for Patients With Chronic Low Back Pain Associated With Active Discopathy: A Randomized Trial,” Annals of Internal Medicine 166 (2017): 547–556. 28319997 10.7326/M16-1700

[124] I. Tavares , E. Thomas , C. Cyteval , et al., “Intradiscal Glucocorticoids Injection in Chronic Low Back Pain With Active Discopathy: A Randomized Controlled Study,” Annals of Physical and Rehabilitation Medicine 64 (2021): 101396. 32461125 10.1016/j.rehab.2020.05.003

[125] J. W. Li , R. L. Wang , J. Xu , et al., “Methylene Blue Prevents Osteoarthritis Progression and Relieves Pain in Rats via Upregulation of Nrf2/PRDX1,” Acta Pharmacologica Sinica 43 (2022): 417–428. 33833406 10.1038/s41401-021-00646-z PMC8792025

[126] S. P. Cohen , K. A. Williams , C. Kurihara , et al., “Multicenter, Randomized, Comparative Cost‐Effectiveness Study Comparing 0, 1, and 2 Diagnostic Medial Branch (Facet Joint Nerve) Block Treatment Paradigms Before Lumbar Facet Radiofrequency Denervation,” Anesthesiology 113 (2010): 395–405. 20613471 10.1097/ALN.0b013e3181e33ae5

[127] S. W. Lee and H. C. Han , “Methylene Blue Application to Lessen Pain: Its Analgesic Effect and Mechanism,” Frontiers in Neuroscience 15 (2021): 663650. 34079436 10.3389/fnins.2021.663650 PMC8165385

[128] G. Gupta , M. Radhakrishna , J. Chankowsky , and J. F. Asenjo , “Methylene Blue in the Treatment of Discogenic Low Back Pain,” Pain Physician 15 (2012): 333–338. 22828687

[129] X. Zhang , J. Hao , Z. Hu , and H. Yang , “Clinical Evaluation and Magnetic Resonance Imaging Assessment of Intradiscal Methylene Blue Injection for the Treatment of Discogenic Low Back Pain,” Pain Physician 19 (2016): E1189–E1195. 27906950

[130] J. W. Kallewaard , V. M. Wintraecken , J. W. Geurts , et al., “A Multicenter Randomized Controlled Trial on the Efficacy of Intradiscal Methylene Blue Injection for Chronic Discogenic Low Back Pain: The IMBI Study,” Pain 160 (2019): 945–953. 30730862 10.1097/j.pain.0000000000001475

[131] B. Peng , X. Pang , Y. Wu , C. Zhao , and X. Song , “A Randomized Placebo‐Controlled Trial of Intradiscal Methylene Blue Injection for the Treatment of Chronic Discogenic Low Back Pain,” Pain 149 (2010): 124–129. 20167430 10.1016/j.pain.2010.01.021

[132] B. Peng , Y. Zhang , S. Hou , W. Wu , and X. Fu , “Intradiscal Methylene Blue Injection for the Treatment of Chronic Discogenic Low Back Pain,” European Spine Journal 16 (2007): 33–38. 16496191 10.1007/s00586-006-0076-1 PMC2198898

[133] D. S. Levi , S. Horn , and E. Walko , “Intradiskal Methylene Blue Treatment for Diskogenic Low Back Pain,” PM & R: The Journal of Injury, Function, and Rehabilitation 6 (2014): 1030–1037. 10.1016/j.pmrj.2014.04.008 24780850

[134] M. Deng , H. Huang , Y. G. Ma , Y. Zhou , Q. Chen , and P. Xie , “Intradiskal Injection of Methylene Blue for Discogenic Back Pain: A Meta‐Analysis of Randomized Controlled Trials,” Journal of Neurological Surgery. Part A, Central European Neurosurgery 82 (2021): 161–165. 33477188 10.1055/s-0040-1721015

[135] X. Guo , W. Ding , L. Liu , and S. Yang , “Intradiscal Methylene Blue Injection for Discogenic Low Back Pain: A Meta‐Analysis,” Pain Practice 19 (2019): 118–129. 30039642 10.1111/papr.12725

[136] L. Zhao , L. Manchikanti , A. D. Kaye , and A. Abd‐Elsayed , “Treatment of Discogenic Low Back Pain: Current Treatment Strategies and Future Options—A Literature Review,” Current Pain and Headache Reports 23 (2019): 86. 31707499 10.1007/s11916-019-0821-x

[137] L. Zhang , Y. Liu , Z. Huang , et al., “Toxicity Effects of Methylene Blue on Rat Intervertebral Disc Annulus Fibrosus Cells,” Pain Physician 22 (2019): 155–164. 30921981

[138] R. R. Andrade , O. B. Oliveira‐Neto , L. T. Barbosa , I. O. Santos , C. F. Sousa‐Rodrigues , and F. T. Barbosa , “Effectiveness of Ozone Therapy Compared to Other Therapies for Low Back Pain: A Systematic Review With Meta‐Analysis of Randomized Clinical Trials,” Brazilian Journal of Anesthesiology (English Edition) 69 (2019): 493–501. 10.1016/j.bjane.2019.06.007 PMC9391853 31521383

[139] T. Costa , D. Linhares , M. Ribeiro da Silva , and N. Neves , “Ozone Therapy for Low Back Pain. A Systematic Review,” Acta Reumatológica Portuguesa 43 (2018): 172–181. 30414366

[140] T. Ercalik and M. Kilic , “Efficacy of Intradiscal Ozone Therapy With or Without Periforaminal Steroid Injection on Lumbar Disc Herniation: A Double‐Blinded Controlled Study,” Pain Physician 23 (2020): 477–484. 32967390

[141] F. N. Magalhaes , L. Dotta , A. Sasse , M. J. Teixera , and E. T. Fonoff , “Ozone Therapy as a Treatment for Low Back Pain Secondary to Herniated Disc: A Systematic Review and Meta‐Analysis of Randomized Controlled Trials,” Pain Physician 15 (2012): E115–E129. 22430658

[142] J. Steppan , T. Meaders , M. Muto , and K. J. Murphy , “A Metaanalysis of the Effectiveness and Safety of Ozone Treatments for Herniated Lumbar Discs,” Journal of Vascular and Interventional Radiology 21 (2010): 534–548. 20188591 10.1016/j.jvir.2009.12.393

[143] L. Canovas , J. Oduña , A. Huete , L. Alonso , M. Couñago , and S. Rojas , “Radiofrecuencia pulsada (Rf) y ozono intradiscal en el alivio del dolor discogénico: Experiencia en 51 casos,” Revista de la Sociedad Española del Dolor 22 (2015): 27–31.

[144] J. Ma , T. Wang , M. Lang , W. Wang , D. Xu , and H. Long , “Clinical Study of Treatment Discogenic Low Back Pain With CT Guided Ozone Injection,” Journal of Shandong University (Health Sciences) 48 (2010): 108–112.

[145] B. Zhao , G. H. Shao , Y. Yu , Y. F. Zhou , B. Zhong , and C. He , “Preliminary Report for Treatment of Discogenic Low Back Pain With Combined Percutaneous Laser and O_2_‐O_3_ Mixture,” Zhongguo Gu Shang 21 (2008): 391–392. 19108478

[146] D. Papadopoulos , C. Batistaki , and G. Kostopanagiotou , “Comparison of the Efficacy Between Intradiscal Gelified Ethanol (Discogel) Injection and Intradiscal Combination of Pulsed Radiofrequency and Gelified Ethanol (Discogel) Injection for Chronic Discogenic Low Back Pain Treatment. A Randomized Double‐Blind Clinical Study,” Pain Medicine 21 (2020): 2713–2718. 32196110 10.1093/pm/pnaa025

[147] K. Latka , K. Kozlowska , M. Waligora , et al., “Efficacy of DiscoGel in Treatment of Degenerative Disc Disease: A Prospective 1‐Year Observation of 67 Patients,” Brain Sciences 11 (2021): 1434. 34827432 10.3390/brainsci11111434 PMC8615618

[148] Y. Choi , M. H. Park , and K. Lee , “Tissue Engineering Strategies for Intervertebral Disc Treatment Using Functional Polymers,” Polymers (Basel) 11 (2019): 872. 31086085 10.3390/polym11050872 PMC6572548

[149] J. Alsousou , A. Ali , K. Willett , and P. Harrison , “The Role of Platelet‐Rich Plasma in Tissue Regeneration,” Platelets 24 (2013): 173–182. 22647081 10.3109/09537104.2012.684730

[150] K. Akeda , H. S. An , R. Pichika , et al., “Platelet‐Rich Plasma (PRP) Stimulates the Extracellular Matrix Metabolism of Porcine Nucleus Pulposus and Anulus Fibrosus Cells Cultured in Alginate Beads,” Spine 31 (2006): 959–966. 16641770 10.1097/01.brs.0000214942.78119.24

[151] E. Anitua , I. Andia , B. Ardanza , P. Nurden , and A. T. Nurden , “Autologous Platelets as a Source of Proteins for Healing and Tissue Regeneration,” Thrombosis and Haemostasis 91 (2004): 4–15. 14691563 10.1160/TH03-07-0440

[152] Y. A. Tuakli‐Wosornu , A. Terry , K. Boachie‐Adjei , et al., “Lumbar Intradiskal Platelet‐Rich Plasma (PRP) Injections: A Prospective, Double‐Blind, Randomized Controlled Study,” PM & R: The Journal of Injury, Function, and Rehabilitation 8 (2016): 1–10; quiz 10. 10.1016/j.pmrj.2015.08.010 26314234

[153] R. Alves and R. Grimalt , “A Review of Platelet‐Rich Plasma: History, Biology, Mechanism of Action, and Classification,” Skin Appendage Disorders 4 (2018): 18–24. 29457008 10.1159/000477353 PMC5806188

[154] Y. Zhang , A. Chee , E. J. Thonar , and H. S. An , “Intervertebral Disk Repair by Protein, Gene, or Cell Injection: A Framework for Rehabilitation‐Focused Biologics in the Spine,” PM & R: The Journal of Injury, Function, and Rehabilitation 3 (2011): S88–S94. 10.1016/j.pmrj.2011.04.020 21703587

[155] A. Navani , L. Manchikanti , S. L. Albers , et al., “Responsible, Safe, and Effective Use of Biologics in the Management of low Back Pain: American Society of Interventional Pain Physicians (ASIPP) Guidelines,” Pain Physician 22 (2019): S1–s74. 30717500

[156] J. W. Lee , O. H. Kwon , T. K. Kim , et al., “Platelet‐Rich Plasma: Quantitative Assessment of Growth Factor Levels and Comparative Analysis of Activated and Inactivated Groups,” Archives of Plastic Surgery 40 (2013): 530–535. 24086805 10.5999/aps.2013.40.5.530 PMC3785585

[157] R. E. Marx , “Platelet‐Rich Plasma (PRP): What Is PRP and What Is Not PRP?,” Implant Dentistry 10 (2001): 225–228. 11813662 10.1097/00008505-200110000-00002

[158] P. Bendinelli , E. Matteucci , G. Dogliotti , et al., “Molecular Basis of Anti‐Inflammatory Action of Platelet‐Rich Plasma on Human Chondrocytes: Mechanisms of NF‐κB Inhibition via HGF,” Journal of Cellular Physiology 225 (2010): 757–766. 20568106 10.1002/jcp.22274

[159] G. M. van Buul , W. L. Koevoet , N. Kops , et al., “Platelet‐Rich Plasma Releasate Inhibits Inflammatory Processes in Osteoarthritic Chondrocytes,” American Journal of Sports Medicine 39 (2011): 2362–2370. 21856929 10.1177/0363546511419278

[160] A. Kabiri , E. Esfandiari , A. Esmaeili , B. Hashemibeni , A. Pourazar , and M. Mardani , “Platelet‐Rich Plasma Application in Chondrogenesis,” Advanced Biomedical Research 3 (2014): 138. 25161985 10.4103/2277-9175.135156 PMC4139981

[161] T. E. Foster , B. L. Puskas , B. R. Mandelbaum , M. B. Gerhardt , and S. A. Rodeo , “Platelet‐Rich Plasma: From Basic Science to Clinical Applications,” American Journal of Sports Medicine 37 (2009): 2259–2272. 19875361 10.1177/0363546509349921

[162] K. Masuda , “Biological Repair of the Degenerated Intervertebral Disc by the Injection of Growth Factors,” European Spine Journal 17 Suppl 4 (2008): 441–451. 19005698 10.1007/s00586-008-0749-z PMC2587664

[163] S. Z. Wang , Q. Chang , J. Lu , and C. Wang , “Growth Factors and Platelet‐Rich Plasma: Promising Biological Strategies for Early Intervertebral Disc Degeneration,” International Orthopaedics 39 (2015): 927–934. 25653173 10.1007/s00264-014-2664-8

[164] U. G. Longo , N. Papapietro , S. Petrillo , E. Franceschetti , N. Maffulli , and V. Denaro , “Mesenchymal Stem Cell for Prevention and Management of Intervertebral Disc Degeneration,” Stem Cells International 2012 (2012): 921053. 22550520 10.1155/2012/921053 PMC3328194

[165] J. Tavakoli , A. D. Diwan , and J. L. Tipper , “Advanced Strategies for the Regeneration of Lumbar Disc Annulus Fibrosus,” International Journal of Molecular Sciences 21 (2020): 4889. 32664453 10.3390/ijms21144889 PMC7402314

[166] A. F. Steinert , L. Rackwitz , F. Gilbert , U. Nöth , and R. S. Tuan , “Concise Review: The Clinical Application of Mesenchymal Stem Cells for Musculoskeletal Regeneration: Current Status and Perspectives,” Stem Cells Translational Medicine 1 (2012): 237–247. 23197783 10.5966/sctm.2011-0036 PMC3659848

[167] M. F. Pittenger , A. M. Mackay , S. C. Beck , et al., “Multilineage Potential of Adult Human Mesenchymal Stem Cells,” Science 284 (1999): 143–147. 10102814 10.1126/science.284.5411.143

[168] J. M. Gimble , A. J. Katz , and B. A. Bunnell , “Adipose‐Derived Stem Cells for Regenerative Medicine,” Circulation Research 100 (2007): 1249–1260. 17495232 10.1161/01.RES.0000265074.83288.09 PMC5679280

[169] S. Wakao , Y. Kuroda , F. Ogura , T. Shigemoto , and M. Dezawa , “Regenerative Effects of Mesenchymal Stem Cells: Contribution of Muse Cells, a Novel Pluripotent Stem Cell Type That Resides in Mesenchymal Cells,” Cells 1 (2012): 1045–1060. 24710542 10.3390/cells1041045 PMC3901150

[170] A. I. Caplan , “All MSCs Are Pericytes?,” Cell Stem Cell 3 (2008): 229–230. 18786406 10.1016/j.stem.2008.08.008

[171] S. M. Richardson , G. Kalamegam , P. N. Pushparaj , et al., “Mesenchymal Stem Cells in Regenerative Medicine: Focus on Articular Cartilage and Intervertebral Disc Regeneration,” Methods 99 (2016): 69–80. 26384579 10.1016/j.ymeth.2015.09.015

[172] K. D. Kim , “Stem Cells and Discogenic Low Back Pain,” Spine 41 Suppl 7 (2016): S11–S12. 27015058 10.1097/BRS.0000000000001424

[173] G. Vadalà , L. Ambrosio , F. Russo , R. Papalia , and V. Denaro , “Stem Cells and Intervertebral Disc Regeneration Overview‐What They Can and Can't Do,” International Journal of Spine Surgery 15 (2021): 40–53. 34376495 10.14444/8054 PMC8092931

[174] J. Wang , Y. Tao , X. Zhou , et al., “The Potential of Chondrogenic Pre‐Differentiation of Adipose‐Derived Mesenchymal Stem Cells for Regeneration in Harsh Nucleus Pulposus Microenvironment,” Experimental Biology and Medicine (Maywood, N.J.) 241 (2016): 2104–2111. 10.1177/1535370216662362 PMC5102140 27488396

[175] E. Kubrova , R. S. D'Souza , C. L. Hunt , Q. Wang , A. J. van Wijnen , and W. Qu , “Injectable Biologics: What Is the Evidence?,” American Journal of Physical Medicine & Rehabilitation 99 (2020): 950–960. 32209835 10.1097/PHM.0000000000001407

[176] D. A. Frauchiger , S. R. Heeb , R. D. May , M. Wöltje , L. M. Benneker , and B. Gantenbein , “Differentiation of MSC and Annulus Fibrosus Cells on Genetically Engineered Silk Fleece‐Membrane‐Composites Enriched for GDF‐6 or TGF‐β3,” Journal of Orthopaedic Research 36 (2018): 1324–1333. 29058815 10.1002/jor.23778

[177] J. Stergar , L. Gradisnik , T. Velnar , and U. Maver , “Intervertebral Disc Tissue Engineering: A Brief Review,” Bosnian Journal of Basic Medical Sciences 19 (2019): 130–137. 30726701 10.17305/bjbms.2019.3778 PMC6535390

[178] L. E. Clarke , J. C. McConnell , M. J. Sherratt , B. Derby , S. M. Richardson , and J. A. Hoyland , “Growth Differentiation Factor 6 and Transforming Growth Factor‐Beta Differentially Mediate Mesenchymal Stem Cell Differentiation, Composition, and Micromechanical Properties of Nucleus Pulposus Constructs,” Arthritis Research & Therapy 16 (2014): R67. 24618041 10.1186/ar4505 PMC4060243

[179] J. V. Stoyanov , B. Gantenbein‐Ritter , A. Bertolo , et al., “Role of Hypoxia and Growth and Differentiation Factor‐5 on Differentiation of Human Mesenchymal Stem Cells Towards Intervertebral Nucleus Pulposus‐Like Cells,” European Cells and Materials 21 (2011): 533–547. 21710444 10.22203/ecm.v021a40

[180] J. Clouet , C. Vinatier , C. Merceron , et al., “The Intervertebral Disc: From Pathophysiology to Tissue Engineering,” Joint, Bone, Spine 76 (2009): 614–618. 19819178 10.1016/j.jbspin.2009.07.002

[181] S. H. Peck , J. R. Bendigo , J. W. Tobias , et al., “Hypoxic Preconditioning Enhances Bone Marrow‐Derived Mesenchymal Stem Cell Survival in a Low Oxygen and Nutrient‐Limited 3D Microenvironment,” Cartilage 12 (2021): 512–525. 30971109 10.1177/1947603519841675 PMC8461160

[182] S. B. G. Blanquer , A. W. H. Gebraad , S. Miettinen , A. A. Poot , D. W. Grijpma , and S. P. Haimi , “Differentiation of Adipose Stem Cells Seeded Towards Annulus Fibrosus Cells on a Designed Poly (Trimethylene Carbonate) Scaffold Prepared by Stereolithography,” Journal of Tissue Engineering and Regenerative Medicine 11 (2017): 2752–2762. 27375236 10.1002/term.2170

[183] J. C. Liao , “Cell Therapy Using Bone Marrow‐Derived Stem Cell Overexpressing BMP‐7 for Degenerative Discs in a Rat Tail Disc Model,” International Journal of Molecular Sciences 17 (2016): 147. 26805824 10.3390/ijms17020147 PMC4783881

[184] B. Peng , B. Xu , W. Wu , L. Du , T. Zhang , and J. Zhang , “Efficacy of Intradiscal Injection of Platelet‐Rich Plasma in the Treatment of Discogenic Low Back Pain: A Single‐Arm Meta‐Analysis,” Medicine (Baltimore) 102 (2023): e33112. 36897725 10.1097/MD.0000000000033112 PMC9997826

[185] M. C. Chang and D. Park , “The Effect of Intradiscal Platelet‐Rich Plasma Injection for Management of Discogenic Lower Back Pain: A Meta‐Analysis,” Journal of Pain Research 14 (2021): 505–512. 33642874 10.2147/JPR.S292335 PMC7903948

[186] J. Sanapati , L. Manchikanti , S. Atluri , et al., “Do Regenerative Medicine Therapies Provide Long‐Term Relief in Chronic Low Back Pain: A Systematic Review and Metaanalysis,” Pain Physician 21 (2018): 515–540. 30508983

[187] M. Basso , L. Cavagnaro , A. Zanirato , et al., “What Is the Clinical Evidence on Regenerative Medicine in Intervertebral Disc Degeneration?,” Musculoskeletal Surgery 101 (2017): 93–104. 28191592 10.1007/s12306-017-0462-3

[188] S. Muthu , M. Jeyaraman , G. Chellamuthu , N. Jeyaraman , R. Jain , and M. Khanna , “Does the Intradiscal Injection of Platelet Rich Plasma Have Any Beneficial Role in the Management of Lumbar Disc Disease?,” Global Spine Journal 12 (2022): 503–514. 33840260 10.1177/2192568221998367 PMC9121148

[189] T. Hirase , R. A. JackIi , K. R. Sochacki , J. D. Harris , and B. K. Weiner , “Systemic Review: Is an Intradiscal Injection of Platelet‐Rich Plasma for Lumbar Disc Degeneration Effective?,” Cureus 12 (2020): e8831. 32607308 10.7759/cureus.8831 PMC7320640

[190] B. J. Schneider , C. Hunt , A. Conger , et al., “The Effectiveness of Intradiscal Biologic Treatments for Discogenic Low Back Pain: A Systematic Review,” Spine Journal 22 (2022): 226–237. 10.1016/j.spinee.2021.07.015 34352363

[191] K. Amirdelfan , H. Bae , T. McJunkin , et al., “Allogeneic Mesenchymal Precursor Cells Treatment for Chronic Low Back Pain Associated With Degenerative Disc Disease: A Prospective Randomized, Placebo‐Controlled 36‐Month Study of Safety and Efficacy,” Spine Journal 21 (2021): 212–230. 10.1016/j.spinee.2020.10.004 33045417

[192] D. C. Noriega , F. Ardura , R. Hernández‐Ramajo , et al., “Intervertebral Disc Repair by Allogeneic Mesenchymal Bone Marrow Cells: A Randomized Controlled Trial,” Transplantation 101 (2017): 1945–1951. 27661661 10.1097/TP.0000000000001484

[193] M. Monfett , J. Harrison , K. Boachie‐Adjei , and G. Lutz , “Intradiscal Platelet‐Rich Plasma (PRP) Injections for Discogenic Low Back Pain: An Update,” International Orthopaedics 40 (2016): 1321–1328. 27073034 10.1007/s00264-016-3178-3

[194] X. Pang , H. Yang , and B. Peng , “Human Umbilical Cord Mesenchymal Stem Cell Transplantation for the Treatment of Chronic Discogenic Low Back Pain,” Pain Physician 17 (2014): E525–E530. 25054402

[195] C. Elabd , C. J. Centeno , J. R. Schultz , G. Lutz , T. Ichim , and F. J. Silva , “Intra‐Discal Injection of Autologous, Hypoxic Cultured Bone Marrow‐Derived Mesenchymal Stem Cells in Five Patients With Chronic Lower Back Pain: A Long‐Term Safety and Feasibility Study,” Journal of Translational Medicine 14 (2016): 253. 27585696 10.1186/s12967-016-1015-5 PMC5009698

[196] H. Kumar , D. H. Ha , E. J. Lee , et al., “Safety and Tolerability of Intradiscal Implantation of Combined Autologous Adipose‐Derived Mesenchymal Stem Cells and Hyaluronic Acid in Patients With Chronic Discogenic Low Back Pain: 1‐Year Follow‐Up of a Phase I Study,” Stem Cell Research & Therapy 8 (2017): 262. 29141662 10.1186/s13287-017-0710-3 PMC5688755

[197] L. Orozco , R. Soler , C. Morera , M. Alberca , A. Sánchez , and J. García‐Sancho , “Intervertebral Disc Repair by Autologous Mesenchymal Bone Marrow Cells: A Pilot Study,” Transplantation 92 (2011): 822–828. 21792091 10.1097/TP.0b013e3182298a15

[198] M. A. Zielinski , N. E. Evans , H. Bae , et al., “Safety and Efficacy of Platelet Rich Plasma for Treatment of Lumbar Discogenic Pain: A Prospective, Multicenter, Randomized, Double‐Blind Study,” Pain Physician 25 (2022): 29–34. 35051141

[199] A. Navani , M. Ambach , R. Navani , and J. Wei , “Biologics for Lumbar Discogenic Pain: 18 Month Follow‐Up for Safety and Efficacy,” IPM Reports 2 (2018): 111–118.

[200] D. Jain , T. Goyal , N. Verma , A. K. Paswan , and R. K. Dubey , “Intradiscal Platelet‐Rich Plasma Injection for Discogenic Low Back Pain and Correlation With Platelet Concentration: A Prospective Clinical Trial,” Pain Medicine 21 (2020): 2719–2725. 32869064 10.1093/pm/pnaa254

[201] J. Cheng , K. A. Santiago , J. T. Nguyen , J. L. Solomon , and G. E. Lutz , “Treatment of Symptomatic Degenerative Intervertebral Discs With Autologous Platelet‐Rich Plasma: Follow‐Up at 5–9 Years,” Regenerative Medicine 14 (2019): 831–840. 31464577 10.2217/rme-2019-0040 PMC6770415

[202] D. Levi , S. Horn , S. Tyszko , J. Levin , C. Hecht‐Leavitt , and E. Walko , “Intradiscal Platelet‐Rich Plasma Injection for Chronic Discogenic Low Back Pain: Preliminary Results From a Prospective Trial,” Pain Medicine 17 (2016): 1010–1022. 26814283 10.1093/pm/pnv053

[203] K. Akeda , K. Ohishi , K. Masuda , et al., “Intradiscal Injection of Autologous Platelet‐Rich Plasma Releasate to Treat Discogenic Low Back Pain: A Preliminary Clinical Trial,” Asian Spine Journal 11 (2017): 380–389. 28670405 10.4184/asj.2017.11.3.380 PMC5481592

[204] A. Hiyama , R. Skubutyte , D. Markova , et al., “Hypoxia Activates the Notch Signaling Pathway in Cells of the Intervertebral Disc: Implications in Degenerative Disc Disease,” Arthritis and Rheumatism 63 (2011): 1355–1364. 21305512 10.1002/art.30246 PMC3613279

[205] T. K. C. Vo , Y. Tanaka , and K. Kawamura , “Ovarian Rejuvenation Using Autologous Platelet‐Rich Plasma,” Endocrine 2 (2021): 15–27.

[206] R. E. B. Fitzsimmons , M. S. Mazurek , A. Soos , and C. A. Simmons , “Mesenchymal Stromal/Stem Cells in Regenerative Medicine and Tissue Engineering,” Stem Cells International 2018 (2018): 8031718. 30210552 10.1155/2018/8031718 PMC6120267

[207] G. Vadalà , G. Sowa , M. Hubert , L. G. Gilbertson , V. Denaro , and J. D. Kang , “Mesenchymal Stem Cells Injection in Degenerated Intervertebral Disc: Cell Leakage May Induce Osteophyte Formation,” Journal of Tissue Engineering and Regenerative Medicine 6 (2012): 348–355. 21671407 10.1002/term.433

[208] R. Derby , B. Eek , S. H. Lee , K. S. Seo , and B. J. Kim , “Comparison of Intradiscal Restorative Injections and Intradiscal Electrothermal Treatment (IDET) in the Treatment of Low Back Pain,” Pain Physician 7 (2004): 63–66. 16868613

[209] M. R. Miller , R. S. Mathews , and K. D. Reeves , “Treatment of Painful Advanced Internal Lumbar Disc Derangement With Intradiscal Injection of Hypertonic Dextrose,” Pain Physician 9 (2006): 115–121. 16703971

[210] P. Chen , L. Ning , P. Qiu , et al., “Photo‐Crosslinked Gelatin‐Hyaluronic Acid Methacrylate Hydrogel‐Committed Nucleus Pulposus‐Like Differentiation of Adipose Stromal Cells for Intervertebral Disc Repair,” Journal of Tissue Engineering and Regenerative Medicine 13 (2019): 682–693. 30808066 10.1002/term.2841

[211] Z. Zhou , M. Gao , F. Wei , et al., “Shock Absorbing Function Study on Denucleated Intervertebral Disc With or Without Hydrogel Injection Through Static and Dynamic Biomechanical Tests In Vitro,” BioMed Research International 2014 (2014): 461724. 25045680 10.1155/2014/461724 PMC4090528

[212] Y. Moriguchi , M. Alimi , T. Khair , et al., “Biological Treatment Approaches for Degenerative Disk Disease: A Literature Review of In Vivo Animal and Clinical Data,” Global Spine Journal 6 (2016): 497–518. 27433434 10.1055/s-0036-1571955 PMC4947401

[213] D. R. Pereira , J. Silva‐Correia , J. M. Oliveira , and R. L. Reis , “Hydrogels in Acellular and Cellular Strategies for Intervertebral Disc Regeneration,” Journal of Tissue Engineering and Regenerative Medicine 7 (2013): 85–98. 22072398 10.1002/term.500

[214] W. Yin , K. Pauza , W. J. Olan , J. F. Doerzbacher , and K. J. Thorne , “Intradiscal Injection of Fibrin Sealant for the Treatment of Symptomatic Lumbar Internal Disc Disruption: Results of a Prospective Multicenter Pilot Study With 24‐Month Follow‐Up,” Pain Medicine 15 (2014): 16–31. 24152079 10.1111/pme.12249

[215] A. Ceylan , I. Aşik , G. E. Özgencil , and B. Erken , “Clinical Results of Intradiscal Hydrogel Administration (GelStix) in Lumbar Degenerative Disc Disease,” Turkish Journal of Medical Sciences 49 (2019): 1634–1639. 31655507 10.3906/sag-1901-1 PMC7518664

[216] S. P. Cohen , D. Wenzell , R. W. Hurley , et al., “A Double‐Blind, Placebo‐Controlled, Dose‐Response Pilot Study Evaluating Intradiscal Etanercept in Patients With Chronic Discogenic Low Back Pain or Lumbosacral Radiculopathy,” Anesthesiology 107 (2007): 99–105. 17585221 10.1097/01.anes.0000267518.20363.0d

[217] T. Sainoh , S. Orita , M. Miyagi , et al., “Single Intradiscal Administration of the Tumor Necrosis Factor‐Alpha Inhibitor, Etanercept, for Patients With Discogenic Low Back Pain,” Pain Medicine 17 (2016): 40–45. 26243249 10.1111/pme.12892

[218] T. Sainoh , S. Orita , M. Miyagi , et al., “Single Intradiscal Injection of the Interleukin‐6 Receptor Antibody Tocilizumab Provides Short‐Term Relief of Discogenic Low Back Pain; Prospective Comparative Cohort Study,” Journal of Orthopaedic Science 21 (2016): 2–6. 26755382 10.1016/j.jos.2015.10.005

[219] J. A. Saal and J. S. Saal , “Intradiscal Electrothermal Treatment for Chronic Discogenic Low Back Pain: A Prospective Outcome Study With Minimum 1‐Year Follow‐Up,” Spine 25 (2000): 2622–2627. 11034647 10.1097/00007632-200010150-00013

[220] M. Karasek and N. Bogduk , “Intradiscal Electrothermal Annuloplasty: Percutaneous Treatment of Chronic Discogenic Low Back Pain,” Techniques in Regional Anesthesia and Pain Management 5 (2001): 130–135.

[221] C. M. Bono , K. Iki , A. Jalota , K. Dawson , and S. R. Garfin , “Temperatures Within the Lumbar Disc and Endplates During Intradiscal Electrothermal Therapy: Formulation of a Predictive Temperature Map in Relation to Distance From the Catheter,” Spine 29 (2004): 1124–1129; discussion 1130–1121. 15131441 10.1097/00007632-200405150-00014

[222] R. V. Shah , G. E. Lutz , J. Lee , S. B. Doty , and S. Rodeo , “Intradiskal Electrothermal Therapy: A Preliminary Histologic Study,” Archives of Physical Medicine and Rehabilitation 82 (2001): 1230–1237. 11552196 10.1053/apmr.2001.23897

[223] R. Derby , R. M. Baker , C. H. Lee , and P. A. Anderson , “Evidence‐Informed Management of Chronic Low Back Pain With Intradiscal Electrothermal Therapy,” Spine Journal 8 (2008): 80–95. 10.1016/j.spinee.2007.10.018 18164457

[224] D. S. Kloth , D. S. Fenton , G. B. Andersson , and J. E. Block , “Intradiscal Electrothermal Therapy (IDET) for the Treatment of Discogenic Low Back Pain: Patient Selection and Indications for Use,” Pain Physician 11 (2008): 659–668. 18850030

[225] L. Kapural , N. Mekhail , Z. Korunda , and A. Basali , “Intradiscal Thermal Annuloplasty for the Treatment of Lumbar Discogenic Pain in Patients With Multilevel Degenerative Disc Disease,” Anesthesia and Analgesia 99 (2004): 472–476, table of contents. 15271727 10.1213/01.ANE.0000133245.67491.90

[226] R. Ravikanth , “A Review of Discogenic Pain Management by Interventional Techniques,” Journal of Craniovertebral Junction and Spine 11 (2020): 4–8. 32549705 10.4103/jcvjs.JCVJS_19_20 PMC7274356

[227] T. S. Eckel and A. O. Ortiz , “Intradiscal Electrothermal Therapy in the Treatment of Discogenic Low Back Pain,” Techniques in Vascular and Interventional Radiology 5 (2002): 217–222. 12599173 10.1053/tvir.2002.36430

[228] S. P. Cohen , T. Larkin , S. Abdi , A. Chang , and M. Stojanovic , “Risk Factors for Failure and Complications of Intradiscal Electrothermal Therapy: A Pilot Study,” Spine 28 (2003): 1142–1147. 12782982 10.1097/01.BRS.0000067269.31377.6A

[229] M. Estefan and V. Estefan , “Intradiscal Electrothermal Therapy,” in StatPearls. StatPearls Publishing Copyright 2024 (StatPearls Publishing LLC, 2024). 31747212

[230] K. J. Pauza , S. Howell , P. Dreyfuss , J. H. Peloza , K. Dawson , and N. Bogduk , “A Randomized, Placebo‐Controlled Trial of Intradiscal Electrothermal Therapy for the Treatment of Discogenic Low Back Pain,” Spine Journal 4 (2004): 27–35. 10.1016/j.spinee.2003.07.001 14749191

[231] B. J. Freeman , R. D. Fraser , C. M. Cain , D. J. Hall , and D. C. Chapple , “A Randomized, Double‐Blind, Controlled Trial: Intradiscal Electrothermal Therapy Versus Placebo for the Treatment of Chronic Discogenic Low Back Pain,” Spine 30 (2005): 2369–2377; discussion 2378. 16261111 10.1097/01.brs.0000186587.43373.f2

[232] D. Appleby , G. Andersson , and M. Totta , “Meta‐Analysis of the Efficacy and Safety of Intradiscal Electrothermal Therapy (IDET),” Pain Medicine 7 (2006): 308–316. 16898940 10.1111/j.1526-4637.2006.00172.x

[233] B. J. Freeman , “IDET: A Critical Appraisal of the Evidence,” European Spine Journal 15 Suppl 3 (2006): S448–S457. 16868786 10.1007/s00586-006-0156-2 PMC2335390

[234] S. Helm Ii , T. T. Simopoulos , M. Stojanovic , S. Abdi , and M. A. El Terany , “Effectiveness of Thermal Annular Procedures in Treating Discogenic Low Back Pain,” Pain Physician 20 (2017): 447–470. 28934777

[235] J. Simon , M. McAuliffe , F. Shamim , N. Vuong , and A. Tahaei , “Discogenic Low Back Pain,” Physical Medicine and Rehabilitation Clinics of North America 25 (2014): 305–317. 24787335 10.1016/j.pmr.2014.01.006

[236] L. Kapural and N. Mekhail , “Novel Intradiscal Biacuplasty (IDB) for the Treatment of Lumbar Discogenic Pain,” Pain Practice 7 (2007): 130–134. 17559482 10.1111/j.1533-2500.2007.00120.x

[237] L. Kapural , B. Vrooman , S. Sarwar , et al., “A Randomized, Placebo‐Controlled Trial of Transdiscal Radiofrequency, Biacuplasty for Treatment of Discogenic Lower Back Pain,” Pain Medicine 14 (2013): 362–373. 23279658 10.1111/pme.12023

[238] M. C. Battié , T. Videman , K. Gill , et al., “1991 Volvo Award in Clinical Sciences. Smoking and Lumbar Intervertebral Disc Degeneration: An MRI Study of Identical Twins,” Spine 16 (1991): 1015–1021. 1948392

[239] J. Takatalo , J. Karppinen , S. Taimela , et al., “Body Mass Index Is Associated With Lumbar Disc Degeneration in Young Finnish Males: Subsample of Northern Finland Birth Cohort Study 1986,” BMC Musculoskeletal Disorders 14 (2013): 87. 23497297 10.1186/1471-2474-14-87 PMC3599904

[240] J. Takatalo , J. Karppinen , S. Taimela , et al., “Association of Abdominal Obesity With Lumbar Disc Degeneration—A Magnetic Resonance Imaging Study,” PLoS One 8 (2013): e56244. 23418543 10.1371/journal.pone.0056244 PMC3571955

[241] S. P. Cohen , T. Larkin , and D. W. Polly, Jr. , “A Giant Herniated Disc Following Intradiscal Electrothermal Therapy,” Journal of Spinal Disorders & Techniques 15 (2002): 537–541. 12468986 10.1097/00024720-200212000-00020

[242] L. Kapural , B. Vrooman , S. Sarwar , et al., “Radiofrequency Intradiscal Biacuplasty for Treatment of Discogenic Lower Back Pain: A 12‐Month Follow‐Up,” Pain Medicine 16 (2015): 425–431. 25339501 10.1111/pme.12595

[243] M. J. Desai , L. Kapural , J. D. Petersohn , et al., “A Prospective, Randomized, Multicenter, Open‐Label Clinical Trial Comparing Intradiscal Biacuplasty to Conventional Medical Management for Discogenic Lumbar Back Pain,” Spine 41 (2016): 1065–1074. 26689579 10.1097/BRS.0000000000001412

[244] M. J. Desai , L. Kapural , J. D. Petersohn , et al., “Twelve‐Month Follow‐Up of a Randomized Clinical Trial Comparing Intradiscal Biacuplasty to Conventional Medical Management for Discogenic Lumbar Back Pain,” Pain Medicine 18 (2017): 751–763. 27570246 10.1093/pm/pnw184

[245] M. M. Elawady , H. G. E. Nassar , T. M. Elgammal , A. A. Abdel Hafez , and A. A. Bessar , “Discogenic Chronic Low Back Pain Management: Intradiscal Radiofrequency Biacuplasty Versus Conservative Treatment,” Egyptian Journal of Radiology and Nuclear Medicine 55 (2024): 198.

[246] H. Karaman , A. Tüfek , G. Kavak , et al., “6‐Month Results of TransDiscal Biacuplasty on Patients With Discogenic Low Back Pain: Preliminary Findings,” International Journal of Medical Sciences 8 (2010): 1–8. 21197258 10.7150/ijms.8.1 PMC3005544

[247] P. M. Finch , L. M. Price , and P. D. Drummond , “Radiofrequency Heating of Painful Annular Disruptions: One‐Year Outcomes,” Journal of Spinal Disorders & Techniques 18 (2005): 6–13. 15687845 10.1097/01.bsd.0000143312.08303.5d

[248] G. Kvarstein , L. Måwe , A. Indahl , et al., “A Randomized Double‐Blind Controlled Trial of Intra‐Annular Radiofrequency Thermal Disc Therapy—A 12‐Month Follow‐Up,” Pain 145 (2009): 279–286. 19647940 10.1016/j.pain.2009.05.001

[249] S. Gautam , V. Rastogi , A. Jain , and A. P. Singh , “Comparative Evaluation of Oxygen‐Ozone Therapy and Combined Use of Oxygen‐Ozone Therapy With Percutaneous Intradiscal Radiofrequency Thermocoagulation for the Treatment of Lumbar Disc Herniation,” Pain Practice 11 (2011): 160–166. 20642485 10.1111/j.1533-2500.2010.00409.x

[250] G. A. Barendse , S. G. van Den Berg , A. H. Kessels , W. E. Weber , and M. van Kleef , “Randomized Controlled Trial of Percutaneous Intradiscal Radiofrequency Thermocoagulation for Chronic Discogenic Back Pain: Lack of Effect From a 90‐Second 70 C Lesion,” Spine 26 (2001): 287–292. 11224865 10.1097/00007632-200102010-00014

[251] D. Sun , Q. Li , Y. Tang , et al., “Comparison of Coblation Annuloplasty and Radiofrequency Thermocoagulation for Treatment of Lumbar Discogenic Pain,” Medicine (Baltimore) 96 (2017): e8538. 29381927 10.1097/MD.0000000000008538 PMC5708926

[252] O. Erçelen , E. Bulutçu , T. Oktenoglu , et al., “Radiofrequency Lesioning Using Two Different Time Modalities for the Treatment of Lumbar Discogenic Pain: A Randomized Trial,” Spine 28 (2003): 1922–1927. 12973135 10.1097/01.BRS.0000083326.39944.73

[253] L. Zhang , X. L. Ding , X. L. Zhao , J. N. Wang , Y. P. Li , and M. Tian , “Fluoroscopy‐Guided Bipolar Radiofrequency Thermocoagulation Treatment for Discogenic Low Back Pain,” Chinese Medical Journal 129 (2016): 2313–2318. 27647190 10.4103/0366-6999.190682 PMC5040017

[254] A. Teixeira and M. E. Sluijter , “Intradiscal High‐Voltage, Long‐Duration Pulsed Radiofrequency for Discogenic Pain: A Preliminary Report,” Pain Medicine 7 (2006): 424–428. 17014601 10.1111/j.1526-4637.2006.00138.x

[255] S. Fukui , K. Nitta , N. Iwashita , H. Tomie , S. Nosaka , and O. Rohof , “Results of Intradiscal Pulsed Radiofrequency for Lumbar Discogenic Pain: Comparison With Intradiscal Electrothermal Therapy,” Korean Journal of Pain 25 (2012): 155–160. 22787545 10.3344/kjp.2012.25.3.155 PMC3389319

[256] O. Rohof , “Intradiscal Pulsed Radiofrequency Application Following Provocative Discography for the Management of Degenerative Disc Disease and Concordant Pain: A Pilot Study,” Pain Practice 12 (2012): 342–349. 22008239 10.1111/j.1533-2500.2011.00512.x

[257] Y. J. Jung , D. G. Lee , Y. W. Cho , and S. H. Ahn , “Effect of Intradiscal Monopolar Pulsed Radiofrequency on Chronic Discogenic Back Pain Diagnosed by Pressure‐Controlled Provocative Discography: A One Year Prospective Study,” Annals of Rehabilitation Medicine 36 (2012): 648–656. 23185729 10.5535/arm.2012.36.5.648 PMC3503940

[258] D. Papadopoulos , G. Kostopanagiotou , A. Lemonis , and C. Batistaki , “Intradiscal Combination of Pulsed Radiofrequency and Gelified Ethanol for the Treatment of Chronic Discogenic Low Back Pain,” Pain Medicine 15 (2014): 881–883. 24506167 10.1111/pme.12346

[259] F. J. Gerges , S. R. Lipsitz , and S. S. Nedeljkovic , “A Systematic Review on the Effectiveness of the Nucleoplasty Procedure for Discogenic Pain,” Pain Physician 13 (2010): 117–132. 20309378

[260] P. M. Eichen , N. Achilles , V. Konig , et al., “Nucleoplasty, a Minimally Invasive Procedure for Disc Decompression: A Systematic Review and Meta‐Analysis of Published Clinical Studies,” Pain Physician 17 (2014): E149–E173. 24658486

[261] V. Singh , C. Piryani , and K. Liao , “Role of Percutaneous Disc Decompression Using Coblation in Managing Chronic Discogenic Low Back Pain: A Prospective, Observational Study,” Pain Physician 7 (2004): 419–425. 16858482

[262] L. He , X. Hu , Y. Tang , X. Li , S. Zheng , and J. Ni , “Efficacy of Coblation Annuloplasty in Discogenic Low Back Pain: A Prospective Observational Study,” Medicine (Baltimore) 94 (2015): e846. 25984672 10.1097/MD.0000000000000846 PMC4602569

[263] N. S. Kumar , S. M. Shah , B. W. Tan , S. Juned , and K. Yao , “Discogenic Axial Back Pain: Is There a Role for Nucleoplasty?,” Asian Spine Journal 7 (2013): 314–321. 24353849 10.4184/asj.2013.7.4.314 PMC3863658

[264] F. Al‐Zain , J. Lemcke , T. Killeen , U. Meier , and A. Eisenschenk , “Minimally Invasive Spinal Surgery Using Nucleoplasty: A 1‐Year Follow‐Up Study,” Acta Neurochirurgica 150 (2008): 1257–1262. 19023515 10.1007/s00701-008-0150-z

[265] S. P. Cohen , S. Williams , C. Kurihara , S. Griffith , and T. M. Larkin , “Nucleoplasty With or Without Intradiscal Electrothermal Therapy (IDET) as a Treatment for Lumbar Herniated Disc,” Journal of Spinal Disorders & Techniques 18 Suppl (2005): S119–S124. 15699797 10.1097/01.bsd.0000127823.54485.3f

[266] C. Slipman , M. Frey , A. Lee , J. Richards , Z. Isaac , and M. Nirschl , “Preliminary Data: Side Effects and Complications of Lumbar Nucleoplasty,” NASS Spine Across the Sea, 2003.

[267] D. Sayed , R. K. Naidu , K. V. Patel , et al., “Best Practice Guidelines on the Diagnosis and Treatment of Vertebrogenic Pain With Basivertebral Nerve Ablation From the American Society of Pain and Neuroscience,” Journal of Pain Research 15 (2022): 2801–2819. 36128549 10.2147/JPR.S378544 PMC9482788

[268] J. G. Khalil , M. Smuck , T. Koreckij , et al., “A Prospective, Randomized, Multicenter Study of Intraosseous Basivertebral Nerve Ablation for the Treatment of Chronic Low Back Pain,” Spine Journal 19 (2019): 1620–1632. 10.1016/j.spinee.2019.05.598 31229663

[269] H. S. Kim , P. H. Wu , and I. T. Jang , “Lumbar Degenerative Disease Part 1: Anatomy and Pathophysiology of Intervertebral Discogenic Pain and Radiofrequency Ablation of Basivertebral and Sinuvertebral Nerve Treatment for Chronic Discogenic Back Pain: A Prospective Case Series and Review of Literature,” International Journal of Molecular Sciences 21 (2020): 1483. 32098249 10.3390/ijms21041483 PMC7073116

[270] H. S. Kim , P. H. Wu , and I. T. Jang , “Narrative Review of Pathophysiology and Endoscopic Management of Basivertebral and Sinuvertebral Neuropathy for Chronic Back Pain,” Journal of Korean Neurosurgical Association 66 (2023): 344–355. 10.3340/jkns.2022.0140 PMC10323272 36444421

[271] M. H. Heggeness and B. J. Doherty , “Discography Causes End Plate Deflection,” Spine 18 (1993): 1050–1053. 8367772 10.1097/00007632-199306150-00015

[272] A. Conger , T. R. Burnham , T. Clark , M. Teramoto , and Z. L. McCormick , “The Effectiveness of Intraosseous Basivertebral Nerve Radiofrequency Ablation for the Treatment of Vertebrogenic Low Back Pain: An Updated Systematic Review With Single‐Arm Meta‐Analysis,” Pain Medicine 23 (2022): S50–S62. 35856331 10.1093/pm/pnac070 PMC9297160

[273] V. Tieppo Francio , D. Sherwood , E. Twohey , et al., “Developments in Minimally Invasive Surgical Options for Vertebral Pain: Basivertebral Nerve Ablation—A Narrative Review,” Journal of Pain Research 14 (2021): 1887–1907. 34188535 10.2147/JPR.S287275 PMC8236249

[274] R. Vallejo , A. Gupta , D. L. Cedeno , et al., “Clinical Effectiveness and Mechanism of Action of Spinal Cord Stimulation for Treating Chronic Low Back and Lower Extremity Pain: A Systematic Review,” Current Pain and Headache Reports 24 (2020): 70. 32997170 10.1007/s11916-020-00907-2

[275] B. A. Meyerson and B. Linderoth , “Mechanisms of Spinal Cord Stimulation in Neuropathic Pain,” Neurological Research 22 (2000): 285–292. 10769822 10.1080/01616412.2000.11740672

[276] T. Deer , K. V. Slavin , K. Amirdelfan , et al., “Success Using Neuromodulation With BURST (SUNBURST) Study: Results From a Prospective, Randomized Controlled Trial Using a Novel Burst Waveform,” Neuromodulation 21 (2018): 56–66. 28961366 10.1111/ner.12698

[277] A. Al‐Kaisy , S. Palmisani , D. Pang , et al., “Prospective, Randomized, Sham‐Control, Double Blind, Crossover Trial of Subthreshold Spinal Cord Stimulation at Various Kilohertz Frequencies in Subjects Suffering From Failed Back Surgery Syndrome (SCS Frequency Study),” Neuromodulation 21 (2018): 457–465. 29608229 10.1111/ner.12771

[278] L. Kapural , C. Yu , M. W. Doust , et al., “Comparison of 10‐kHz High‐Frequency and Traditional Low‐Frequency Spinal Cord Stimulation for the Treatment of Chronic Back and Leg Pain: 24‐Month Results From a Multicenter, Randomized, Controlled Pivotal Trial,” Neurosurgery 79 (2016): 667–677. 27584814 10.1227/NEU.0000000000001418 PMC5058646

[279] T. R. Deer , J. S. Grider , T. J. Lamer , et al., “A Systematic Literature Review of Spine Neurostimulation Therapies for the Treatment of Pain,” Pain Medicine 21 (2020): 1421–1432. 32034422 10.1093/pm/pnz353

[280] S. Falowski and A. Sharan , “A Review on Spinal Cord Stimulation,” Journal of Neurosurgical Sciences 56 (2012): 287–298. 23111289

[281] G. Baranidharan , R. Feltbower , B. Bretherton , et al., “One‐Year Results of Prospective Research Study Using 10 kHz Spinal Cord Stimulation in Persistent Nonoperated Low Back Pain of Neuropathic Origin: Maiden Back Study,” Neuromodulation 24 (2021): 479–487. 33351230 10.1111/ner.13345

[282] L. Kapural , J. Jameson , C. Johnson , et al., “Treatment of Nonsurgical Refractory Back Pain With High‐Frequency Spinal Cord Stimulation at 10 kHz: 12‐Month Results of a Pragmatic, Multicenter, Randomized Controlled Trial,” Journal of Neurosurgery. Spine 37 (2022): 1–12. 35148512 10.3171/2021.12.SPINE211301

[283] N. Christelis , B. Simpson , M. Russo , et al., “Persistent Spinal Pain Syndrome: A Proposal for Failed Back Surgery Syndrome and ICD‐11,” Pain Medicine 22 (2021): 807–818. 33779730 10.1093/pm/pnab015 PMC8058770

[284] R. Vallejo , L. M. Zevallos , J. Lowe , and R. Benyamin , “Is Spinal Cord Stimulation an Effective Treatment Option for Discogenic Pain?,” Pain Practice 12 (2012): 194–201. 21797964 10.1111/j.1533-2500.2011.00489.x

[285] M. R. Mons , K. B. Chapman , C. Terwiel , E. A. Joosten , and J. W. Kallewaard , “A Prospective Study of BurstDR Spinal Cord Stimulation for Non‐Operated Discogenic Low Back Pain,” Pain Practice 23 (2023): 234–241. 36373868 10.1111/papr.13181

[286] R. L. Rauck , E. Loudermilk , S. J. Thomson , et al., “Long‐Term Safety of Spinal Cord Stimulation Systems in a Prospective, Global Registry of Patients With Chronic Pain,” Pain Management 13 (2023): 115–127. 36691862 10.2217/pmt-2022-0091

[287] K. A. Williams , M. Gonzalez‐Fernandez , S. Hamzehzadeh , et al., “A Multi‐Center Analysis Evaluating Factors Associated With Spinal Cord Stimulation Outcome in Chronic Pain Patients,” Pain Medicine 12 (2011): 1142–1153. 21749636 10.1111/j.1526-4637.2011.01184.x

[288] I. Dones and V. Levi , “Spinal Cord Stimulation for Neuropathic Pain: Current Trends and Future Applications,” Brain Sciences 8 (2018): 138. 30042314 10.3390/brainsci8080138 PMC6119923

[289] F. Huygen , J. W. Kallewaard , H. Nijhuis , et al., “Effectiveness and Safety of Dorsal Root Ganglion Stimulation for the Treatment of Chronic Pain: A Pooled Analysis,” Neuromodulation 23 (2020): 213–221. 31730273 10.1111/ner.13074 PMC7079258

[290] A. S. Koopmeiners , S. Mueller , J. Kramer , and Q. H. Hogan , “Effect of Electrical Field Stimulation on Dorsal Root Ganglion Neuronal Function,” Neuromodulation 16 (2013): 304–311; discussion 310–301. 23421796 10.1111/ner.12028

[291] K. Suseki , Y. Takahashi , K. Takahashi , T. Chiba , M. Yamagata , and H. Moriya , “Sensory Nerve Fibres From Lumbar Intervertebral Discs Pass Through Rami Communicantes. A Possible Pathway for Discogenic Low Back Pain,” Journal of Bone and Joint Surgery (British Volume) 80 (1998): 737–742. 9699846 10.1302/0301-620x.80b4.8239

[292] J. W. Kallewaard , C. Edelbroek , M. Terheggen , A. Raza , and J. W. Geurts , “A Prospective Study of Dorsal Root Ganglion Stimulation for Non‐Operated Discogenic Low Back Pain,” Neuromodulation 23 (2020): 196–202. 30821901 10.1111/ner.12937

[293] F. Huygen , L. Liem , W. Cusack , and J. Kramer , “Stimulation of the L2‐L3 Dorsal Root Ganglia Induces Effective Pain Relief in the Low Back,” Pain Practice 18 (2018): 205–213. 28486758 10.1111/papr.12591

[294] M. R. Mons , K. B. Chapman , C. Terwiel , E. A. Joosten , and J. W. Kallewaard , “Burst Spinal Cord Stimulation as Compared With L2 Dorsal Root Ganglion Stimulation in Pain Relief for Nonoperated Discogenic Low Back Pain: Analysis of Two Prospective Studies,” Neuromodulation 27 (2024): 172–177. 37191612 10.1016/j.neurom.2023.04.464

[295] T. R. Deer , R. M. Levy , J. Kramer , et al., “Dorsal Root Ganglion Stimulation Yielded Higher Treatment Success Rate for Complex Regional Pain Syndrome and Causalgia at 3 and 12 Months: A Randomized Comparative Trial,” Pain 158 (2017): 669–681. 28030470 10.1097/j.pain.0000000000000814 PMC5359787

[296] M. Kretzschmar , M. Reining , and M. A. Schwarz , “Three‐Year Outcomes After Dorsal Root Ganglion Stimulation in the Treatment of Neuropathic Pain After Peripheral Nerve Injury of Upper and Lower Extremities,” Neuromodulation 24 (2021): 700–707. 32573868 10.1111/ner.13222

[297] R. S. D'Souza , B. Langford , M. Dombovy‐Johnson , and A. Abd‐Elsayed , “Neuromodulation Interventions for the Treatment of Painful Diabetic Neuropathy: A Systematic Review,” Current Pain and Headache Reports 26 (2022): 365–377. 35226258 10.1007/s11916-022-01035-9

[298] J. H. Ghorayeb , A. Chitneni , A. Rupp , A. Parkash , and A. Abd‐Elsayed , “Dorsal Root Ganglion Stimulation for the Treatment of Chronic Pelvic Pain: A Systematic Review,” Pain Practice 23 (2023): 838–846. 37246484 10.1111/papr.13255

[299] R. L. Weiner , A. Yeung , C. Montes Garcia , L. Tyler Perryman , and B. Speck , “Treatment of FBSS Low Back Pain With a Novel Percutaneous DRG Wireless Stimulator: Pilot and Feasibility Study,” Pain Medicine 17 (2016): 1911–1916. 27125284 10.1093/pm/pnw075

[300] Y. F. Han and X. Cong , “Comparison of the Efficacy of Spinal Cord Stimulation and Dorsal Root Ganglion Stimulation in the Treatment of Painful Diabetic Peripheral Neuropathy: A Prospective, Cohort‐Controlled Study,” Frontiers in Neurology 15 (2024): 1366796. 38660091 10.3389/fneur.2024.1366796 PMC11039825

[301] T. Deer , J. Pope , C. Hunter , et al., “Safety Analysis of Dorsal Root Ganglion Stimulation in the Treatment of Chronic Pain,” Neuromodulation 23 (2020): 239–244. 30861617 10.1111/ner.12941 PMC7065079

